# Hofbauer Cells Spread Listeria monocytogenes among Placental Cells and Undergo Pro-Inflammatory Reprogramming while Retaining Production of Tolerogenic Factors

**DOI:** 10.1128/mBio.01849-21

**Published:** 2021-08-17

**Authors:** Siavash Azari, Lauren J. Johnson, Amy Webb, Sophia M. Kozlowski, Xiaoli Zhang, Kara Rood, Amal Amer, Stephanie Seveau

**Affiliations:** a Department of Microbiology, The Ohio State University, Columbus, Ohio, USA; b Department of Microbial Infection and Immunity, The Ohio State University, Columbus, Ohio, USA; c Department of Biomedical Informatics, The Ohio State University, Columbus, Ohio, USA; d Department of Obstetrics and Gynecology, Division of Maternal Fetal Medicine, The Ohio State University, Columbus, Ohio, USA; e Infectious Disease Institute, The Ohio State University, Columbus, Ohio, USA; University of Edinburgh

**Keywords:** Hofbauer cells, *Listeria monocytogenes*, RNA-seq, fetal tolerance, infection, inflammation, macrophage polarization, placenta, transcriptome

## Abstract

Pregnant women are highly susceptible to infection by the bacterial pathogen Listeria monocytogenes, leading to miscarriage, premature birth, and neonatal infection. L. monocytogenes is thought to breach the placental barrier by infecting trophoblasts at the maternal/fetal interface. However, the fate of L. monocytogenes within chorionic villi and how infection reaches the fetus are unsettled. Hofbauer cells (HBCs) are fetal placental macrophages and the only leukocytes residing in healthy chorionic villi, forming a last immune barrier protecting fetal blood from infection. Little is known about the HBCs’ antimicrobial responses to pathogens. Here, we studied L. monocytogenes interaction with human primary HBCs. Remarkably, despite their M2 anti-inflammatory phenotype at basal state, HBCs phagocytose and kill non-pathogenic bacteria like Listeria innocua and display low susceptibility to infection by L. monocytogenes. However, L. monocytogenes can exploit HBCs to spread to surrounding placental cells. Transcriptomic analyses by RNA sequencing revealed that HBCs undergo pro-inflammatory reprogramming upon L. monocytogenes infection, similarly to macrophages stimulated by the potent M1-polarizing agents lipopolysaccharide (LPS)/interferon gamma (IFN-γ). Infected HBCs also express pro-inflammatory chemokines known to promote placental infiltration by maternal leukocytes. However, HBCs maintain the expression of a collection of tolerogenic genes and secretion of tolerogenic cytokines, consistent with their tissue homeostatic role in prevention of fetal rejection. In conclusion, we propose a previously unrecognized model in which HBCs promote the spreading of L. monocytogenes among placental cells and transition to a pro-inflammatory state likely to favor innate immune responses, while maintaining the expression of tolerogenic factors known to prevent maternal anti-fetal adaptive immunity.

## INTRODUCTION

The immunological defense of the fetus is crucial for a successful pregnancy. The placenta plays key roles in protecting the semi-allogeneic fetus against rejection by the maternal immune system, while it ensures efficient immune defense against most pathogens (1, 2). The mechanisms that orchestrate the placental immune functions are still poorly understood. In particular, the role of placental macrophages of fetal origin, called Hofbauer cells (HBCs), is still elusive (3). Pathogens like the Gram-positive bacterium Listeria monocytogenes have evolved virulence mechanisms to breach the maternal/fetal barrier, penetrate chorionic villi where HBCs are located, and reach the fetus, causing miscarriage, preterm birth, and severe infections of the neonate with long-term sequelae (1, 4–7). In this work, we studied the interplay between the model pathogen L. monocytogenes and human HBCs, isolated from healthy term placentas.

**FIG 1:**
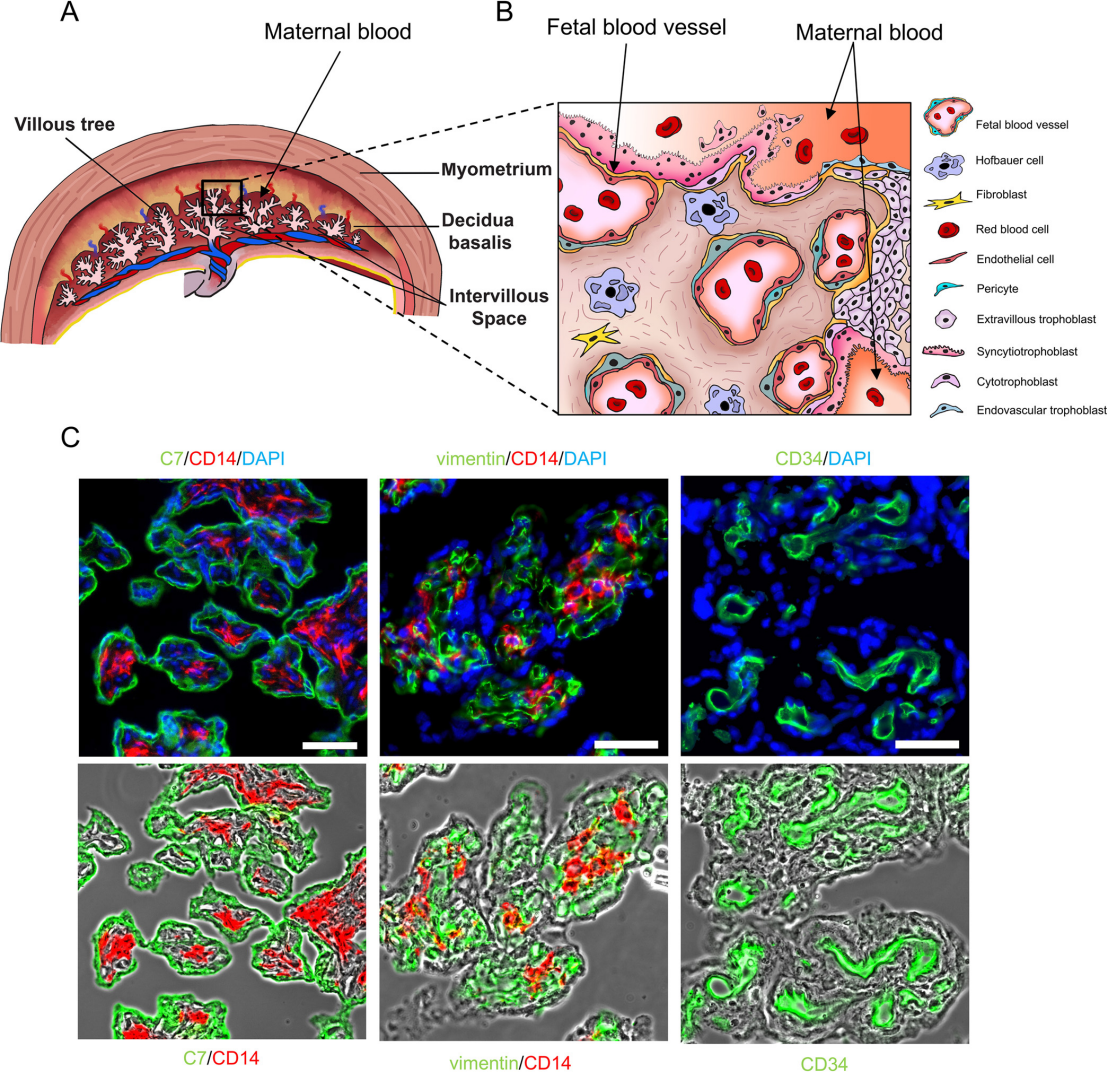
The structure of the human term placenta. (A) Schematic representation of the human term placenta. (B) Representation of a villous tree anchored to the decidua by extravillous trophoblasts. The syncytiotrophoblast, a syncytium that covers chorionic villi, is in direct contact with maternal blood to mediate nutritional and waste exchanges between the mother and fetus. Cytotrophoblasts are mononucleated cells that fuse to form the syncytiotrophoblast. Chorionic villus stroma includes HBCs, fibroblasts, and fetal blood vessels. (C) Labeling of human term chorionic villi to visualize the syncytiotrophoblast and trophoblasts (cytokeratin 7 [C7] labeling). HBCs (CD14), fibroblasts (vimentin), and fetal endothelial cells (CD34) are located in the intravillous space. For each panel, the red- and green-labeled stromal or endothelial cells were overlayed with the corresponding phase-contrast image of the villi to better show the location of each cell type. Corresponding labels are shown under each phase-contrast panel. Scale bars represent 30 μm (left) and 45 μm (middle and right).

The placenta is composed of chorionic villi organized in a tree-like structure covered by an epithelial syncytium, the syncytiotrophoblast. The chorionic villi expose a surface area of 12 to 14 m^2^ and are bathed in maternal blood to capture oxygen and nutrients while releasing fetal waste (Fig. 1A and B) (8, 9). Chorionic villi are anchored into the maternal uterine wall by columns of extravillous trophoblasts (Fig. 1A and B). Previous studies proposed that L. monocytogenes enters the placenta by infecting extravillous trophoblasts or the syncytiotrophoblast (Fig. 1) (10, 11). L. monocytogenes has been observed within chorionic villi of placentas recovered from human listeriosis cases and from animal infection models (11–13). HBCs are abundant fetal macrophages and the only leukocytes present in healthy chorionic villi throughout pregnancy (14–16). However, to date, no studies have characterized the interplay between L. monocytogenes and HBCs. HBCs are tissue-resident macrophages that play crucial roles in placental development and homeostasis, such as promoting angiogenesis, transferring nutrients and maternal antibodies toward fetal blood, and producing tolerogenic factors that protect the placental/fetal unit from rejection by the maternal immune system (3, 17, 18). These macrophages are motile and located between two cell barriers that protect the fetus from infectious agents: trophoblasts at the maternal interface and fetal endothelial cells at the fetal interface (Fig. 1B and C) (14, 16). Therefore, it is critical to uncover whether HBCs are able to control L. monocytogenes infection or if they provide an intracellular replicative niche. Macrophages control infection by removing microbes from tissues via phagocytosis, followed by killing or containment of phagocytosed microbes to prevent their replication. This antimicrobial control primarily occurs via oxidative burst and phagolysosomal fusion, and macrophages use additional mechanisms to limit the growth of cytosolic bacteria (19, 20). L. monocytogenes is a pathogen that can disrupt the phagosomal membrane to escape into the cytosol of infected cells, where it replicates (21). Macrophages have been categorized into two major groups based on their mode of activation: classically activated (M1, pro-inflammatory) and alternatively activated (M2, mostly anti-inflammatory and ensuring tissue homeostatic functions) (22). M1-polarized macrophages are more potent at containing L. monocytogenes infection than M2-polarized macrophages (23). However, the M1/M2 classification is an over-simplification, as macrophage phenotypes exist as a more complex continuum between these two opposite states, which reflects their functional diversity and tissue-specific activities (22). In healthy placentas, HBCs display an M2 anti-inflammatory phenotype characterized by the expression of surface markers like CD163, CD209, and CD206 and the production of anti-inflammatory and tolerogenic cytokines, including interleukin-10 (IL-10) and transforming growth factor beta (TGF-β) (3, 24–26). Therefore, based on their fetal origin and M2-polarized phenotype, HBCs are expected to be highly permissive to L. monocytogenes infection. It is unknown whether infection can repolarize HBCs toward an M1-like pro-inflammatory and antimicrobial phenotype. The anti-inflammatory environment of the placenta is critical during the second and third trimesters of pregnancy, and tilting the balance toward an excessive inflammatory state can lead to adverse pregnancy outcomes (5). Whether HBCs are involved in detrimental placental inflammation by an M1 repolarization, which could contribute to villitis observed in listeriosis cases, is unsettled (27, 28). HBCs were proposed to maintain an M2 phenotype despite pro-inflammatory environmental cues, such as stimulation with the M1-polarizing agents interferon gamma (IFN-γ)/lipopolysaccharide (LPS) (24–26, 29–32). In this work, we measured the HBCs’ susceptibility to L. monocytogenes infection and studied the HBCs’ transcriptome, cytokine production, and expression of surface markers to gain detailed information about their responses to L. monocytogenes. As controls, we exposed HBCs to the potent M1-polarizing agents (IFN-γ/LPS) and used the well-characterized human macrophage model THP-1 cells (M0 [untreated] and M1 [IFN-γ/LPS-polarized]) (33–36). We found that HBCs are able to kill non-pathogenic Listeria innocua and display low susceptibility to L. monocytogenes infection. However, L. monocytogenes cells still replicate in the HBCs and can exploit these cells to spread to other placental/fetal cells. HBCs are plastic; upon infection, they undergo an M1-like repolarization accompanied by the production of numerous chemokines, with the potential to elicit maternal leukocyte infiltration of chorionic villi. However, remarkably, the production of tolerogenic factors remained sustained.

## RESULTS

### Hofbauer cell isolation and surface marker characterization.

HBCs were isolated from healthy term placentas, and their purity was assessed by fluorescence microscopy after 72 h of culture, corresponding to the time point of all infections performed in this article. The pan-leukocyte CD45, M2 macrophage CD163, fetal fibroblast CD90, and trophoblast cytokeratin 7 markers were labeled with fluorescent antibodies (Fig. S1A in the supplemental material) (26, 37). We observed that CD45^+^ cells were highly vacuolated, which is a characteristic of HBCs (Fig. S1B) (37). As expected, 98% of CD45^+^ cells were also CD163^+^, since a minor proportion of HBCs is known to be CD163 low or negative (Fig. S1C) (26, 37). HBC purity, measured as the percentage of CD45^+^ cells, was 92.58% ± 1.28% (mean ± standard error of the mean [SEM]) (*n* = 7 placentas), and at least 90 × 10^6^ HBCs were recovered per placenta, similar to previous reports (26, 37). Trophoblasts and fibroblasts were the only cell contaminants, as the sum of the three cell populations (CD45^+^, CD90^+^, and C7^+^) reached 100% (Fig. S1C). Among these cells, only fibroblasts proliferated, as confirmed by co-labeling cell markers and the Ki67 marker (data not shown), with a doubling time of 17 h. Back calculation indicated that HBC purity was >97% at the time of their isolation, consistent with the purity achieved when negative selection was used (37). Labeling of HBCs with M1-specific (CD80) and M2-specific (CD163, CD14, CD206, and CD209) markers confirmed their M2 phenotype, as more than 90% of HBCs were CD80^−^ CD163^+^ CD14^+^ CD206^+^ (Fig. S1D).

10.1128/mBio.01849-21.1 FIG S1 Analysis of HBC purity and surface marker expression. (A) HBCs were labeled with anti-CD45 (leukocytes), anti-CD163 (macrophages), anti-CD90 (fibroblasts), and anti-cytokeratin 7 (C7, trophoblasts) Abs and with secondary fluorescent Abs. Nuclei were labeled with DAPI. Scale bar represents 30 μm. (B) Phase-contrast image shows typical highly vacuolated HBCs. Scale bar is 15 μm. (C) Fifteen fields were acquired randomly (∼700 HBCs per experimental condition). The percentage of marker-positive cells was calculated as (*N_m_*/*N_n_*) × 100 ± SEM (*n* = 7 placentas), where *N_n_* is the total cell number and *N_m_* the number of cells positive for each marker. Data are from 7 HBC isolations (7 placentas). (D) Cells were labeled with M1- and M2-specific Abs, and the average percentages (±SEM) of HBCs positive for each marker are shown (*n* = 3 placentas). Download FIG S1, PDF file, 2.4 MB. Copyright © 2021 Azari et al. 2021 Azari et al. https://creativecommons.org/licenses/by/4.0/ This content is distributed under the terms of the Creative Commons Attribution 4.0 International license.

### Hofbauer cells control the non-pathogenic bacterial species L. innocua and display low susceptibility to L. monocytogenes infection.

**FIG 2:**
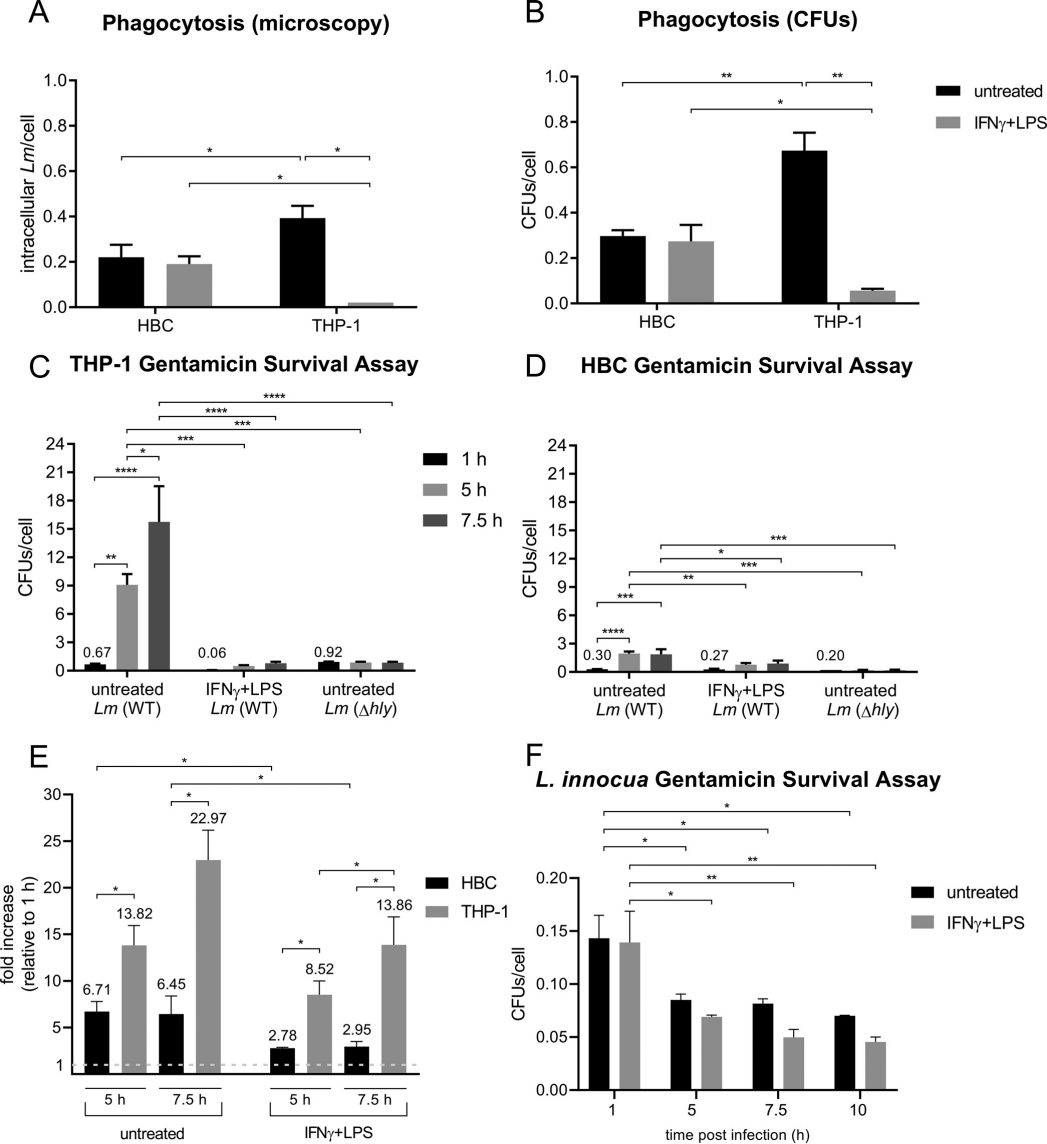
HBCs display low susceptibility to L. monocytogenes infection but kill non-pathogenic L. innocua. HBCs and PMA-differentiated THP-1 cells were stimulated with IFN-γ/LPS or not for 24 h prior to infection with WT or listeriolysin O (LLO)-deficient (Δ*hly*) L. monocytogenes, in duplicate, for the indicated times on the figure. (A and B) Phagocytosis was measured by fluorescence microscopy (A) and by CFU counts (B) 30 min post-infection. For fluorescence microscopy, cells were labeled with DAPI and Abs against CD45 (*N*_CD45_), extracellular bacteria (*N_e_*), and total bacteria (*N_t_*). Fifteen random fields of view (∼700 HBCs per condition) were acquired. Phagocytosis was calculated as the number of intracellular L. monocytogenes per cell with the formula (*N_t_* − *N_e_*)/*N*_CD45_ ± SEM. *n* = 3 placentas: *, *P* < 0.05. For CFU counts, *n* = 3 placentas: *, *P* < 0.05; **, *P* ≤ 0.0043. All *P* values for A and B were calculated by two-way analysis of variance and Sidak’s multiple comparisons tests. (C and D) CFU counts from THP-1 cells (C) or HBCs (D) at the indicated time points on the figure. Data are expressed as the number of CFUs per cell ± SEM. *n* = 3 for each experiment whose results are shown in panels A to D, data obtained from a total of 5 placentas: *, *P* < 0.02; **, *P* < 0.006; ***, *P* < 0.0009; ****, *P* < 0.0001 (two-way analysis of variance and Tukey’s multiple-comparison tests). (E) The fold increases of CFUs/cell from the experiments whose results are shown in panels C and D were calculated relative to the CFUs/cell ± SEM (*n* = 3) at 1 h of infection for HBCs and THP-1 cells. *, *P* ≤ 0.041 (ratio paired Student’s *t* test). (F) Stimulated or untreated HBCs were infected with L. innocua for the indicated times on the figure. Data are expressed as the number of CFUs per cell ± SEM. *n* = 3, different placentas from those used in the experiments whose results are shown in panels A to D: *, *P* < 0.0438; **, *P* ≤ 0.0026 (two-way analysis of variance and Tukey’s multiple-comparison tests).

We analyzed whether HBCs could phagocytose L. monocytogenes and control infection in their basal M2 state, and whether their antimicrobial activity could be enhanced upon exposure to potent M1-polarizing agents, as shown using non-placental macrophages (23). HBCs were stimulated for 24 h with the prototypical M1-polarizing cocktail IFN-γ/LPS (stimulated) or not (untreated). For comparison with a well-characterized human macrophage model, we used phorbol myristate acetate (PMA)-differentiated THP-1 cells, either untreated (M0 phenotype [M0-THP-1 cells]) or stimulated by IFN-γ/LPS for 24 h (M1 phenotype [M1-THP-1 cells]) (33, 34, 38, 39). HBCs and THP-1 cells were infected with wild-type (WT) L. monocytogenes and an isogenic listeriolysin O (LLO)-deficient mutant (Δ*hly*) that fails to escape from the phagosome and replicate in macrophages (40–42). The phagocytic efficiencies were measured by enumeration of fluorescently labeled intracellular bacteria (microscopy) and viable intracellular bacteria (CFU) (Fig. 2A and B). Untreated HBCs were 2-fold less phagocytic than M0-THP-1 cells (Fig. 2A and B). However, stimulated HBCs maintained their phagocytic efficiency, unlike M1-THP-1 cells, whose phagocytic efficiency dropped significantly, by 10-fold (Fig. 2A and B). We next measured the number of viable intracellular bacteria (CFU) at different times post-infection. As previously reported, M1-THP-1 cells controlled L. monocytogenes better than M0-THP-1 cells (Fig. 2C) (34–36). Similarly, stimulated HBCs controlled infection better than untreated HBCs, and remarkably, there was no net increase in viable intracellular bacteria 5 to 7.5 h post-infection in untreated or stimulated HBCs (Fig. 2D). To compare HBCs’ and THP-1 cells’ ability to control phagocytosed L. monocytogenes, the fold changes in viable intracellular bacteria over time were calculated relative to the counts at 1 h of infection to correct for differences in phagocytosis (Fig. 2E). We found that untreated and stimulated HBCs were significantly less susceptible to L. monocytogenes infection than THP-1 cells.

We next analyzed whether HBCs were able to kill the nonpathogenic species Listeria innocua. We found that HBCs were able to kill ∼40% and ∼50% of phagocytosed L. innocua cells 5 h and 10 h post-phagocytosis, respectively (Fig. 2F).

**FIG 3:**
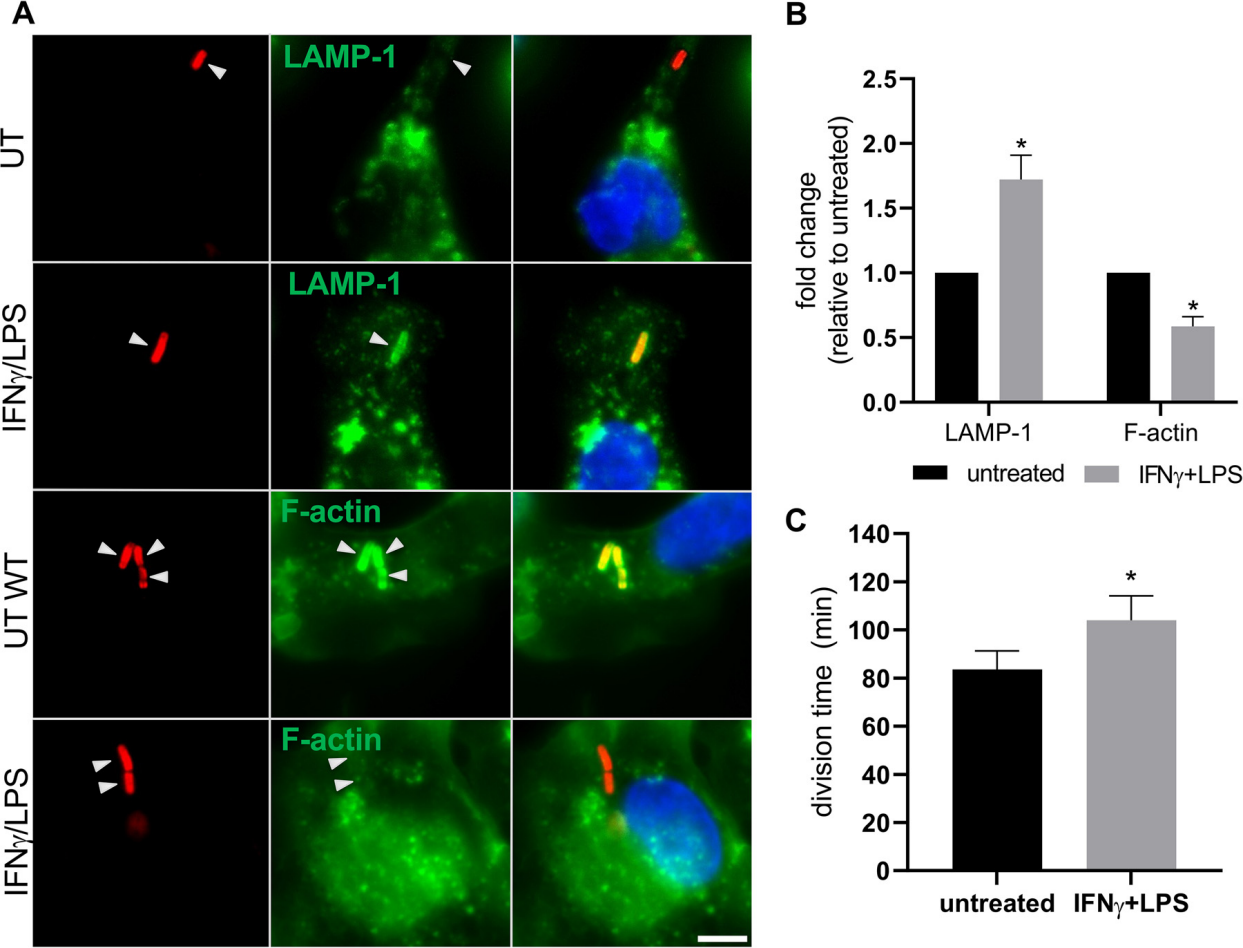
Measurement of LAMP-1 and F-actin recruitment by intracellular L. monocytogenes and cytosolic doubling times. HBCs were stimulated with IFN-γ/LPS or left untreated for 24 h prior to infection with WT or p*actA*-RFP L. monocytogenes. (A) WT L. monocytogenes-infected cells were fixed with PFA 2 h post-infection and labeled with anti-L. monocytogenes fluorescent Abs to label total and extracellular L. monocytogenes. Intracellular L. monocytogenes cells were color-coded in red. LAMP-1 was labeled with anti-human LAMP-1 Abs and secondary Alexa Fluor 488-conjugated Abs (green), and in a second set of samples, F-actin was labeled with Alexa Fluor 488-conjugated phalloidin (green). The levels of LAMP-1-positive (LAMP-1^+^) bacteria were 11.3% and 20% in untreated and stimulated HBCs, respectively. Scale bar represents 5 μm. (B) The percentages of LAMP-1^+^ and F-actin^+^ intracellular bacteria were counted from at least 19 planes (corresponding to a minimum of 500 bacteria) per experimental condition and were expressed as fold change ± SEM in comparison to the results for untreated HBCs. *n* = 3 placentas: *, *P* ≤ 0.0368 (paired Student’s *t* test) (C) Bacterial division time was calculated based on the number of RFP^+^ L. monocytogenes cells 4 to 6 h post-infection. *n* = 3 placentas, different from those used in the experiment whose results are shown in panels A and B: *, *P* = 0.0280 (paired Student’s *t* test).

To determine if stimulated HBCs, similar to stimulated non-placental macrophages, could confine L. monocytogenes in the phagosome better than their non-stimulated counterparts, we measured the percentage of intracellular L. monocytogenes present in the endolysosomal compartments (LAMP-1^+^) 2 h post-infection by fluorescence microscopy (Fig. 3A). As a complementary approach, we measured the percentage of intracellular L. monocytogenes cells that recruited F-actin once they reached the cytosol (Fig. 3A). Consistent with increased L. monocytogenes phagosomal containment in stimulated HBCs, the proportion of LAMP-1^+^ intracellular bacteria increased significantly, whereas the F-actin^+^ intracellular bacteria decreased significantly in a similar proportion (Fig. 3B).

**FIG 4:**
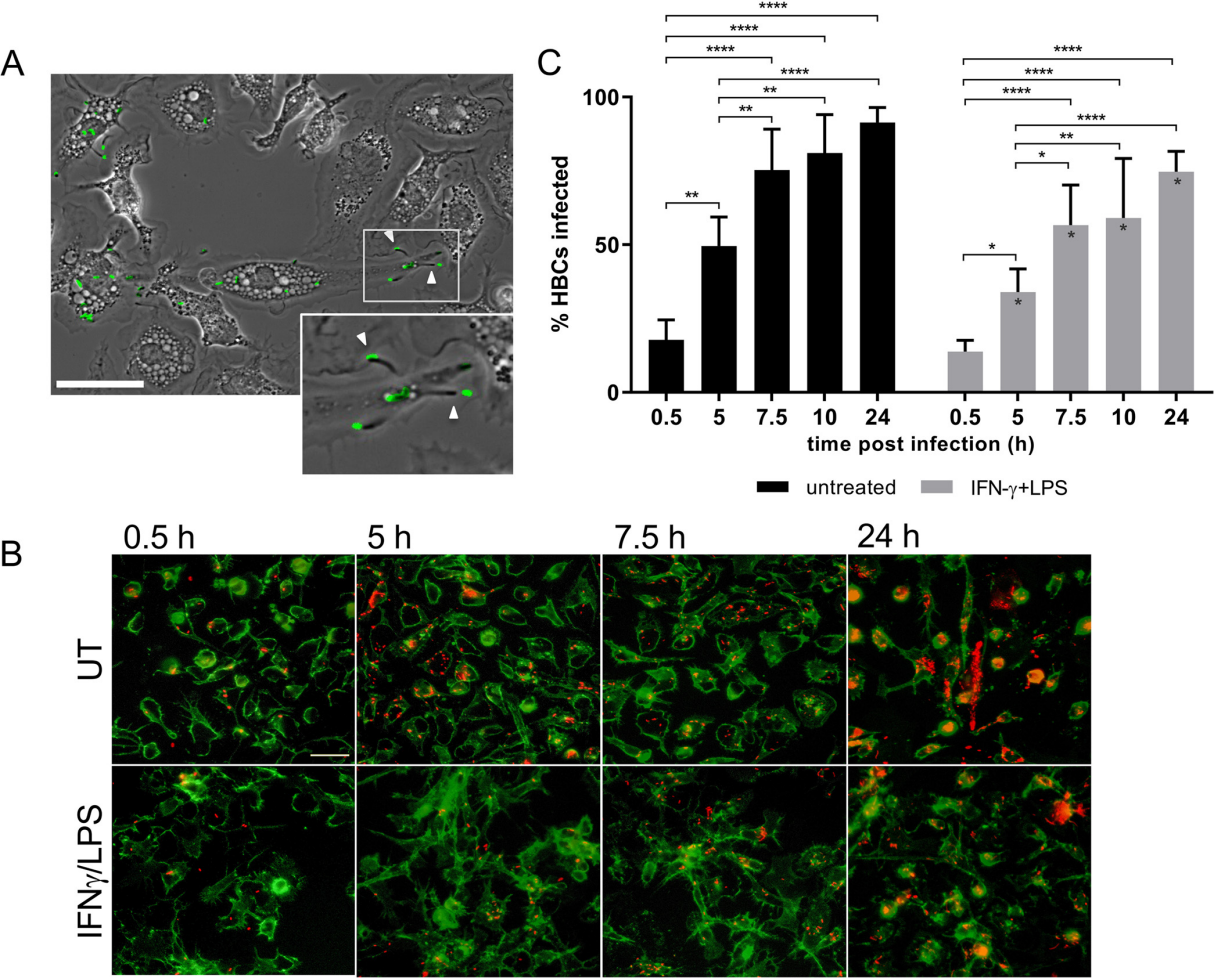
Inter-HBC spreading of L. monocytogenes. HBCs were stimulated with IFN-γ/LPS or left untreated for 24 h prior to infection with WT L. monocytogenes. Cells were infected for up to 24 h in the presence of gentamicin. At the indicated time points on the figure, cells were fixed with PFA and labeled using Abs directed against CD45 (green), against extracellular L. monocytogenes, and against total bacteria following cell permeabilization and nuclei were labeled with DAPI. (A) HBCs were infected with p*actA*-RFP L. monocytogenes for 4 h. Cells were placed on the atmosphere-controlled microscope, and images were recorded for 2 h. The representative picture was taken 5 h post-infection (from Movie S1) and is an overlay of phase-contrast and green color-coded fluorescent L. monocytogenes images. Arrows indicate L. monocytogenes cells with comet tails. Scale bar represents 30 μm. (B) Representative images of infected untreated (UT) and stimulated HBCs in which intracellular L. monocytogenes cells are color-coded in red (selected from 3 independent experiments corresponding to 3 placentas). Scale bar represents 30 μm. (C) Fluorescence images were randomly acquired (∼700 cells per condition), and the average percentages of infected HBCs (with at least 1 intracellular L. monocytogenes) ± SEM were counted over time. *n* = 3, the same placentas as used in the experiment whose results are shown in panel B: *, *P* < 0.0395; **, *P* ≤ 0.0085; ****, *P* < 0.0001. The asterisks on the gray bars (stimulated cells) indicate significant differences in comparison to the corresponding time points in untreated HBCs. *, *P* < 0096. Mixed-effect model where the main effect and interaction of time and treatment were the independent variables, and Tukey’s multiple-comparison test was used for all *P* values generated for the data in panel C.

To determine whether HBC stimulation, in addition to improving the containment of L. monocytogenes in LAMP-1^+^ vacuoles, could also affect the bacterial cytosolic doubling time, cells were infected with p*actA*-RFP L. monocytogenes cells, which express red fluorescent protein (RFP) once they reach the host cell cytosol (Fig. 3C and 4A) (10). The cytosolic doubling time was calculated based on the enumeration of RFP-positive (RFP^+^) bacteria by live-cell fluorescence microscopy 4 to 6 h post-infection (Fig. 3C). The doubling time in untreated HBCs increased significantly, from 83.59 ± 7.70 min to 104.06 ± 10.08 min (mean ± SEM) in IFN-γ-/LPS-stimulated cells (Fig. 3C; Movies S1 and S2).

10.1128/mBio.01849-21.5 MOVIE S1 L. monocytogenes division and motility in untreated HBCs. Untreated HBCs were infected with p*actA*-RFP L. monocytogenes (pseudo-colored in green) at an MOI of 5 for 30 min, followed by incubation with gentamicin-containing medium for 4 h. Phase-contrast and DsRed live-cell imaging was performed in Z series 4 to 6 h post-infection. The time (min) is stamped on the movie. The movie is an overlay of the phase-contrast and RFP channels. Scale bar shows 40 μm. Download Movie S1, MOV file, 18.3 MB. Copyright © 2021 Azari et al. 2021 Azari et al. https://creativecommons.org/licenses/by/4.0/ This content is distributed under the terms of the Creative Commons Attribution 4.0 International license.

10.1128/mBio.01849-21.6 MOVIE S2 L. monocytogenes motility and division in stimulated HBCs. IFN-γ-/LPS-stimulated HBCs were infected with p*actA*-RFP L. monocytogenes (pseudo-colored in green) at an MOI of 5 for 30 min, followed by incubation in gentamicin-containing medium for 4 h. Phase-contrast and DsRed live-cell imaging was performed in Z series 4 to 6 h post-infection. The time (min) is stamped on the movie. The movie is an overlay of the phase-contrast and RFP channels. Scale bar shows 40 μm. Download Movie S2, MOV file, 17.6 MB. Copyright © 2021 Azari et al. 2021 Azari et al. https://creativecommons.org/licenses/by/4.0/ This content is distributed under the terms of the Creative Commons Attribution 4.0 International license.

Collectively, the data show that in their basal state, HBCs can exert antimicrobial activities and display low susceptibility to L. monocytogenes infection compared to an established macrophage model, yet they fail to fully control L. monocytogenes cytosolic growth. Furthermore, HBCs display functional plasticity when exposed to prototypical M1-polarizing agents, as evidenced by significantly increased phagosomal containment and increased L. monocytogenes cytosolic doubling time.

### Hofbauer cells can spread L. monocytogenes to other placental and fetal cells.

**FIG 5:**
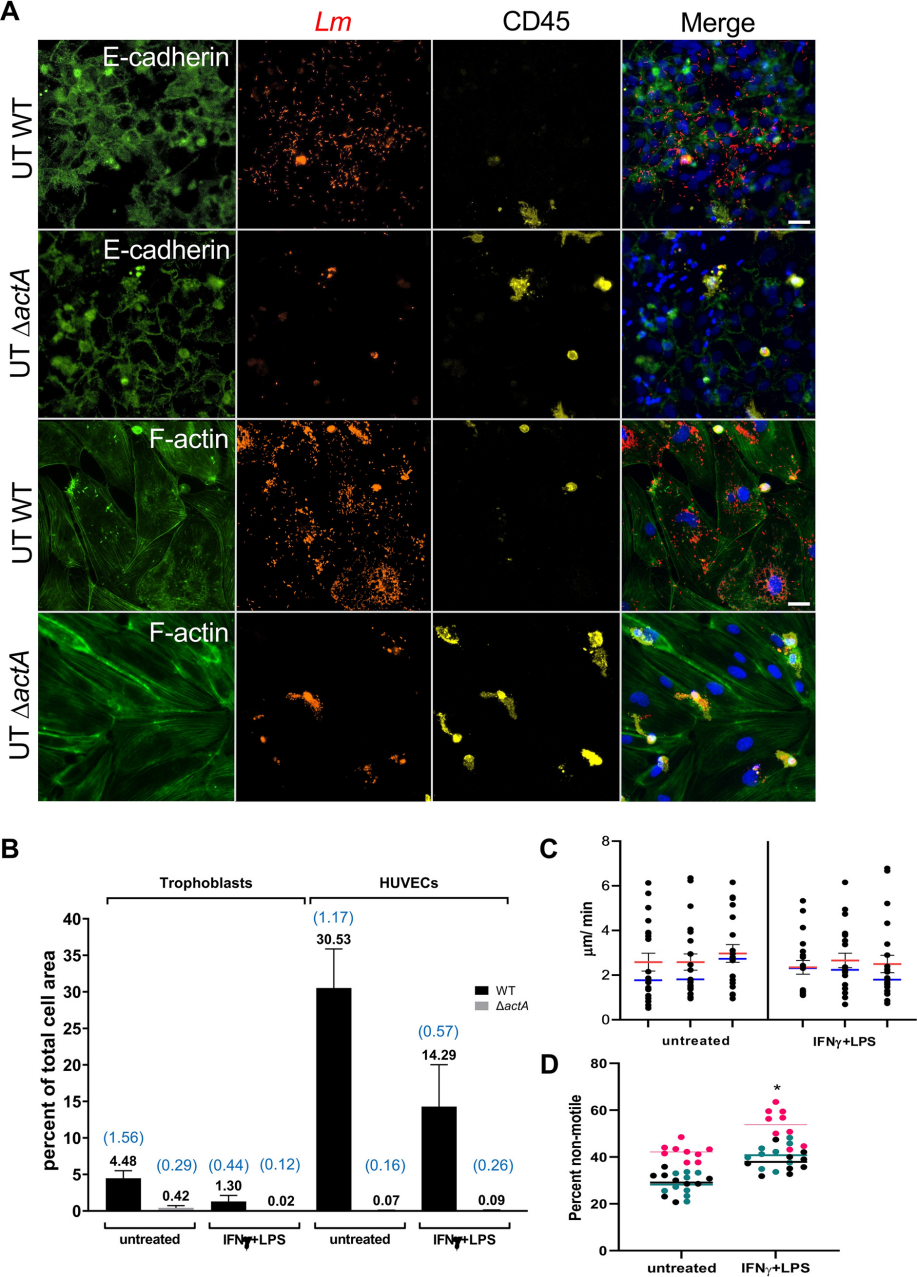
HBCs mediate L. monocytogenes spreading to placental and fetal cells. (A) Untreated HBCs were infected with WT or Δ*actA* L. monocytogenes (MOI of 10) for 5 h. HBCs were detached and transferred onto confluent PHT or HUVEC cultures in duplicate for 18 h in the presence of gentamicin. HBCs and PHTs were isolated from the same placenta, and HUVECs were commercially purchased. Cells were fixed with PFA, permeabilized, and labeled with fluorescent Abs against L. monocytogenes (*Lm*, red), E-cadherin (PHTs, green), and CD45 (HBCs, yellow) and with phalloidin (F-actin in HUVECs, green) and DAPI (nuclei, blue). Scale bar represents 30 μm. Images of the corresponding experiment involving stimulated HBCs are shown in Fig. S2 (the same placenta). (B) Images were randomly acquired (corresponding to at least 14 fields of view, ∼20,000 PHTs and ∼800 HUVECs per experimental condition). The total cell areas of PHTs and HUVECs, the cell areas of infectious foci, and the number of infectious foci were measured in each plane. The data were expressed as the average percentage (numerical value in black on the top of each bar) of cell area covered by infectious foci ± SEM. The numbers of foci per plane are shown in blue on top of each bar. (C, D) Untreated and IFN-γ-/LPS-stimulated HBCs were infected with p*actA*-RFP L. monocytogenes at an MOI of 5. (C) The speed of at least 20 L. monocytogenes cells was measured in μm/min from 3 independent experiments, and individual bacterial speeds for each experiment are represented by dots. Average (red) and median (blue) speeds ± SEM (*n* = 3 placentas: *P* = 0.3110 (paired Student’s *t* test). (D) Percentages of non-motile RFP^+^ L. monocytogenes cells (which remained in a perimeter of 2 μm) relative to the total number of RFP^+^ L. monocytogenes cells. About 140 bacteria were counted in each movie segment. The horizontal lines indicate the mean value for each experiment; the data for the different experiments were color coded. *n* = 3 placentas, the same placentas as used in the experiment whose results are shown in panel C: *, *P* = 0.0102 (paired Student’s *t* test).

The virulence factor ActA, which promotes L. monocytogenes cell-to-cell spread via F-actin-based intracellular motility, is required for placental/fetal infection (12, 43–45). We tested whether HBCs could act as transmission vessels to other placental cells in an ActA-dependent fashion. Live-cell imaging of HBCs infected with p*actA*-RFP L. monocytogenes revealed that cytosolic bacteria were motile and formed extracellular protrusions, which are the structures supporting cell-to-cell spread (Fig. 4A; Movies S1 and S2). We then measured the percentages of L. monocytogenes-infected HBCs by fluorescence microscopy 0.5 to 24 h post-infection (Fig. 4B). Infected cells were defined as cells with one or more intracellular L. monocytogenes. There was a significant increase in the percentages of infected (stimulated and untreated) HBCs over time, which was a manifestation of L. monocytogenes intercellular spreading, because gentamicin in the culture medium killed extracellular bacteria (Fig. 4C). Consistent with better control of L. monocytogenes by stimulated HBCs (Fig. 2), the results in Fig. 4C show that at time points 5, 7.5, 10, and 24 h, the percentages of infected IFN-γ-/LPS-stimulated HBCs were significantly decreased compared to the percentages of infected untreated HBCs. Next, we assessed whether infected HBCs could spread L. monocytogenes to other placental and fetal cells. HBCs were infected with WT or isogenic Δ*actA* L. monocytogenes and were detached and co-cultured overnight on a confluent monolayer of primary human trophoblasts (PHTs; isolated from the same placenta) or primary human umbilical vein endothelial cells (HUVECs; commercially available) in the presence of gentamicin. The data showed that WT L. monocytogenes, but not the Δ*actA* mutant, could spread from infected HBCs to PHTs and HUVECs, regardless of the HBCs’ stimulation state (Fig. 5A and B; Fig. S2). The numbers of infectious foci per cell surface area were similar in PHTs and HUVECs (Fig. 5B). However, the average size of the infectious foci was larger in HUVECs than in PHTs. Also, untreated HBCs appeared to spread infection more efficiently than stimulated HBCs, as characterized by both decreased numbers and sizes of foci in HUVECs and PHTs. This is likely due to the higher intracellular bacterial load in untreated HBCs than in stimulated HBCs (Fig. 2). We tested whether this could also result from more efficient L. monocytogenes motility in untreated HBCs, but the speed of p*actA*-RFP L. monocytogenes cells, measured by live-cell imaging, showed that this was not the case (Fig. 5C). However, there was a significantly higher percentage of non-motile bacteria in stimulated HBCs than in untreated HBCs, consistent with less efficient bacterial spread by stimulated HBCs (Fig. 5D).

10.1128/mBio.01849-21.2 FIG S2 IFN-γ-/LPS-stimulated HBCs mediate L. monocytogenes spreading to placental and fetal cells. IFN-γ-/LPS-stimulated HBCs were infected with WT or Δ*actA* L. monocytogenes at an MOI of 10 for 5 h. HBCs were detached, transferred onto PHTs or HUVECs, and incubated for 18 h in the presence of gentamicin. The cells were PFA fixed and fluorescently labeled for F-actin (phalloidin, HUVECs, green) or with antibodies directed against E-cadherin (PHTs, green), total bacteria (L. monocytogenes, red), and CD45 (HBCs, yellow). Nuclei were stained with DAPI (blue). Scale bar shows 30 μm. Quantifications are shown in Fig. 5B. Download FIG S2, PDF file, 2.3 MB. Copyright © 2021 Azari et al. 2021 Azari et al. https://creativecommons.org/licenses/by/4.0/ This content is distributed under the terms of the Creative Commons Attribution 4.0 International license.

### L. monocytogenes-infected Hofbauer cells undergo pro-inflammatory reprogramming, including the upregulation of cytokines and chemokines known to recruit leukocytes into chorionic villi.

**FIG 6:**
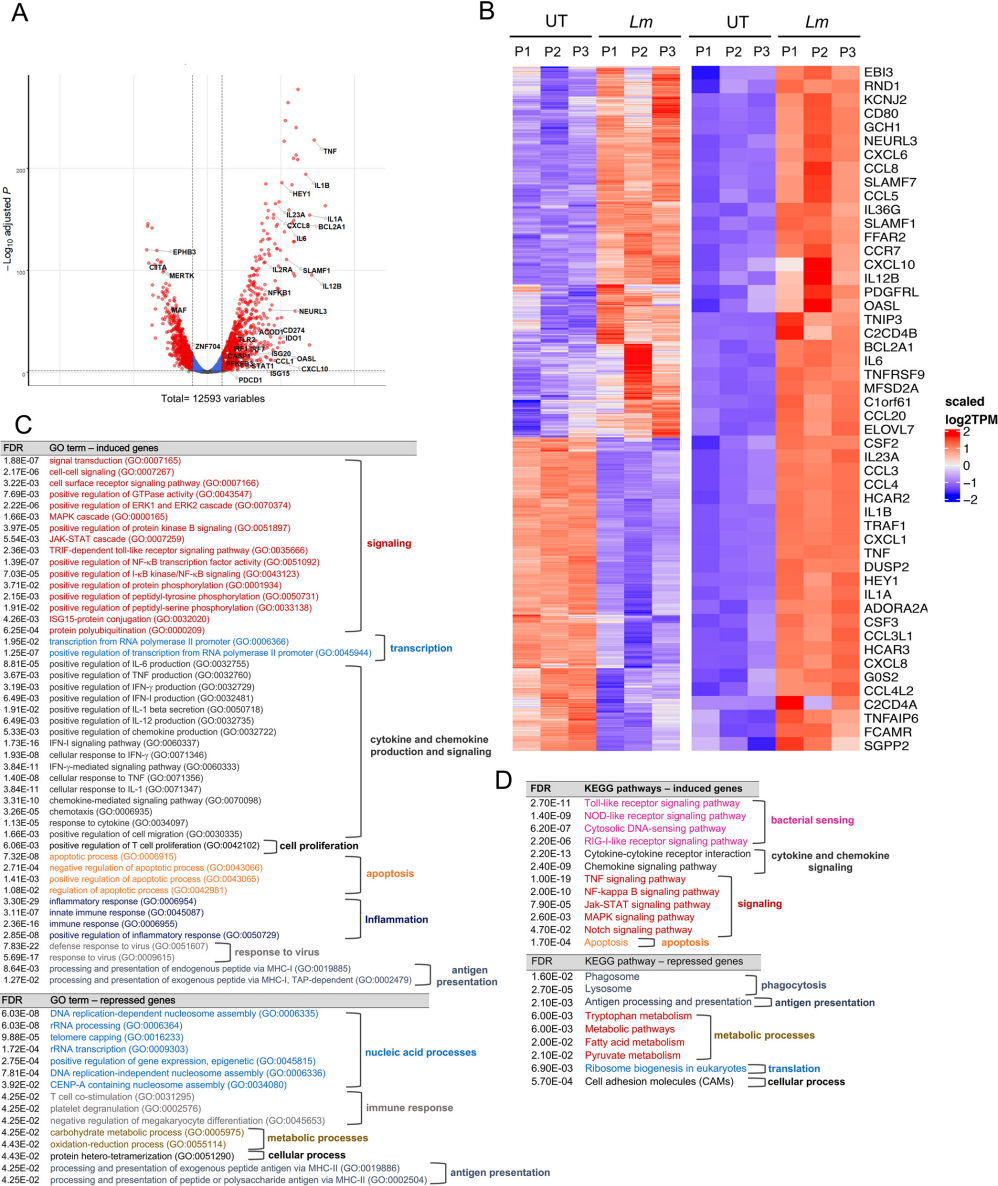
L. monocytogenes-infected Hofbauer cells undergo transcriptional pro-inflammatory reprogramming. Untreated HBCs were washed and infected or not infected with WT L. monocytogenes. After 5 h, RNA was collected, sequenced, and analyzed. (A) Volcano plot of −log_10_ statistical significance (FDR [false discovery rate]-adjusted *P* value) against Log_2_-fold change in L. monocytogenes-infected versus uninfected HBCs. Red dots correspond to differentially expressed (DE) genes with significant and greater than 2-fold changes (FDR-adjusted *P* < 0.05, Log_2_FC > 1 or < −1). Cutoffs indicated by dashed lines. A few genes of interest were annotated on the figure. (B) The heatmaps of non-infected (UT) and L. monocytogenes-infected (*Lm*) HBCs from the 3 placentas are shown. Left, heatmap of all DE genes based on scaled Log_2_ transcripts per million (TPM); right, heatmap of the top 50 protein-coding genes with the highest absolute Log_2_FC values in L. monocytogenes-infected versus non-infected HBCs. Raw counts were normalized with edgeR and converted to TPM. The genes were clustered based on the CPM (counts per million); the same order is used in the TPM, and the samples are in a set order. Red and blue indicate upregulated and downregulated genes in infected cells, respectively. (C) Gene Ontology (GO) analysis of all DE genes using DAVID 6.8, color coded and classified into functional categories (with an EASE score of 0.05 and FDR of <0.05). (D) Kyoto Encyclopedia of Genes and Genomes (KEGG) pathway analysis of all the DE genes, using DAVID 6.8 with an EASE score of 0.05 and FDR of <0.05. The functional categories are color-coded. The list of all significant pathways is available in Table S2.

**FIG 7:**
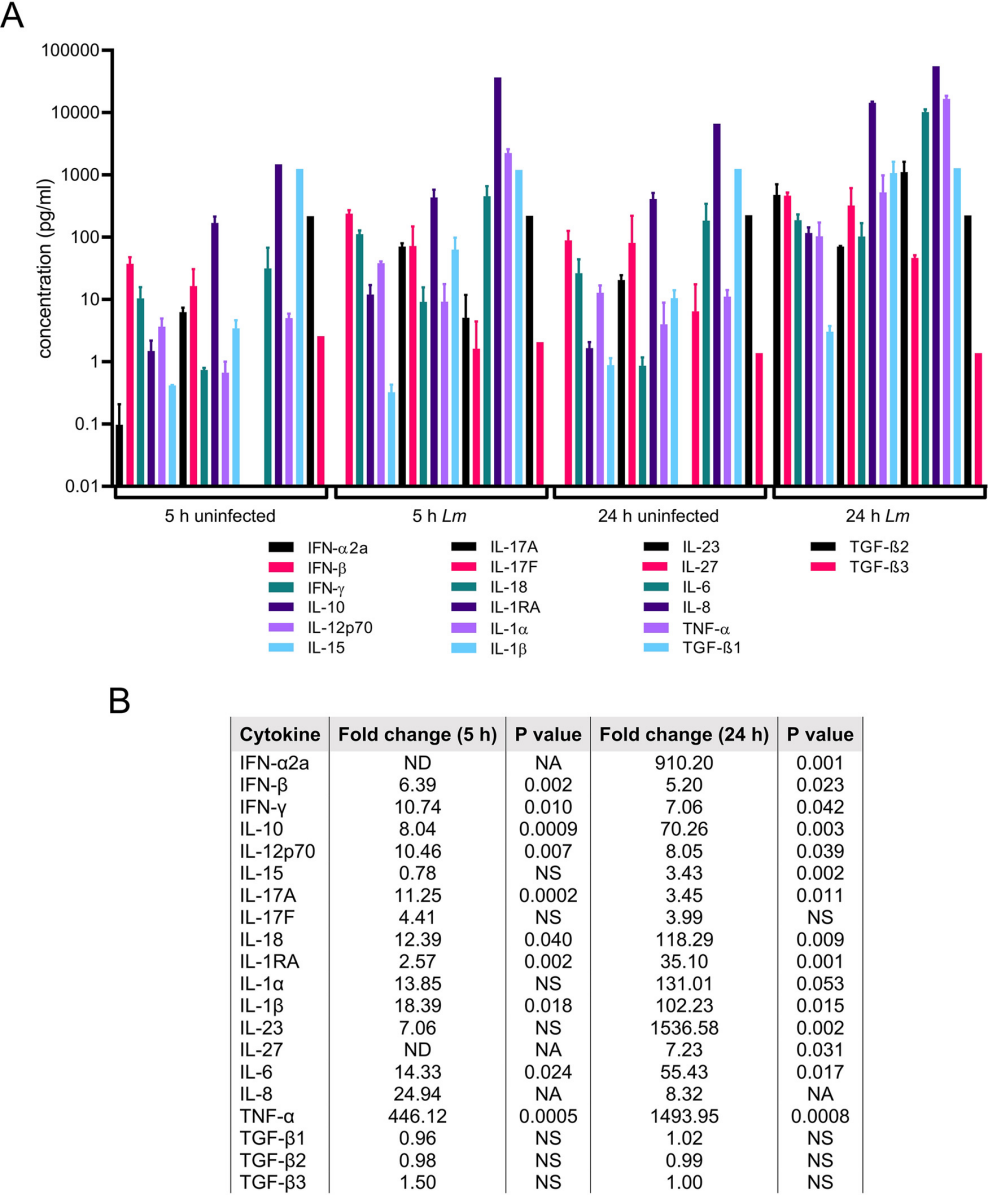
Cytokine array of L. monocytogenes-infected HBCs. Untreated HBCs were washed and infected or not infected with WT L. monocytogenes for 5 and 24 h. The cell culture supernatants were collected and analyzed for the presence of 20 cytokines by using the Meso Scale Discovery (MSD) multiplex cytokine array. (A) Culture supernatants were analyzed in duplicate for each cytokine and experimental condition, and data are the average values for 20 measured cytokines (pg/ml ± SEM). *n* = 3, the same placentas as used in the experiment whose results are shown in Fig. 6. For IL-8 and TGF-β only, samples of the 3 experiments were pooled before measurements. (B) The fold change of each cytokine produced by infected HBCs after 5 h and 24 h of incubation in comparison to expression in the corresponding untreated condition. Ratio paired Student’s *t* test was used to generate the *P* values. ND, not detected (below detection limit); NS, not significant; NA, not applicable (for the pooled samples).

To gain information about HBCs’ plasticity and responses to L. monocytogenes infection, we performed RNA sequencing (RNA-seq) and measured an array of 20 cytokines released by infected HBCs. RNA-seq analysis of HBCs infected for 5 h by WT L. monocytogenes in comparison to control non-infected cells revealed that among the 12,593 genes (expression levels above 2 counts per million [CPM] in at least half of the samples), 1,913 (15.2%) were significantly differentially expressed (DE), including 1,037 (8.2%) that were upregulated and 876 (7.0%) that were downregulated, as illustrated by the volcano plot and heatmaps in Fig. 6A and B. The top 50 DE genes in 5-h-infected HBCs predominantly included genes encoding cytokines, chemokines, and their receptors and regulators (26 out of 50 genes) (Fig. 6B). Gene Ontology (GO) and Kyoto Encyclopedia of Genes and Genomes (KEGG) pathway analyses (DAVID 6.8) of the DE genes revealed that infection led to the upregulation of numerous pro-inflammatory pathways (Fig. 6C and D). These included signaling through pattern recognition receptors (Toll-like receptor 2 [TLR2]/MyD88, RIG-I/cGAS, NOD1/RIPK2, and NLRP3/caspase-1) that converged toward IFN regulatory factor 7 (IRF7) and NF-κB activation (among other transcription factors) and the downstream production of numerous pro-inflammatory mediators, consistent with sensing and responding to extracellular and intracellular Gram-positive bacteria. In accordance with this finding, there was a significant upregulation of immune signaling pathways, including phosphoinositide 3-kinase (PI3K), extracellular signal-regulated kinases 1 and 2 (ERK1/2), mitogen-activated protein kinase (MAPK), Notch, JAK-STAT, and NF-κB, the biosynthetic pathways for a set of cytokines (IFN-α/β, IL-1 α/β, IFN-γ, IL-6, tumor necrosis factor alpha [TNF-α], IL-12, and IL-8), and multiple chemokines, as well as the autocrine responses to some of these mediators (Fig. 6C and D). Importantly, the pro-inflammatory remodeling observed at the transcriptional level was confirmed by significant production of the corresponding cytokines (IFN- α/β, IL-1 α/β, IFN-γ, IL-6, TNF-α, IL-12, and IL-8) by L. monocytogenes-infected HBCs (Fig. 7). The production of IL-18 and IL-1β reflected that L. monocytogenes infection activated caspase-1, in accordance with the significant upregulation of NLRP3 and CASP1 transcripts and associated pathways (Fig. 6) (46). Interestingly, genes coding for T-cell chemokines (CXCL9, CXCL10, CXCL11, CCL3L1, CCL5, CCL1, CCL22, and CCL24) and neutrophil and monocyte/macrophage chemokines (CXCL8 [IL-8], CXCL1, CXCL2, CXCL6, CCL3, CCL4, CCL2, CCL7, and CCL8) were all upregulated (Table S1) (47), as were antimicrobial response pathways and antigen presentation by major histocompatibility complex class I (MHC-I) (Fig. 6). RNA-seq analysis comparing infected HBCs at 5 and 24 h post-infection showed that there were only minor variations in the upregulated pathways 24 h post-infection (Fig. S3 and Table S2). Surprisingly, although many genes were downregulated 5 h post-infection, there was no significant GO or KEGG pathway associated with these genes. Downregulated pathways reached statistical significance (EASE score [a modified Fisher exact *P* value] = 0.05 and FDR < 0.05) 24 h post-infection (Fig. 6C and D; Table S2). These pathways included phagosome formation and maturation, antigen presentation by MHC-II, metabolism (tryptophan, fatty acid, and pyruvate metabolism), transcription regulation, including epigenetic control, and translation (ribosome biogenesis).

10.1128/mBio.01849-21.3 FIG S3 L. monocytogenes-infected Hofbauer cells undergo transcriptional pro-inflammatory reprogramming. HBCs were washed and infected with WT L. monocytogenes for 5 and 24 h. At the indicated times shown on the figure, RNA was collected, sequenced, and analyzed. (A) Volcano plot of −log_10_ statistical significance (FDR-adjusted *P* value) against Log_2_-fold change in 24-h L. monocytogenes-infected versus 5-h L. monocytogenes-infected HBCs. Red dots correspond to differentially expressed (DE) genes, with significant and greater than 2-fold changes (FDR-adjusted *P* < 0.05, Log_2_FC > 1 or < −1). Cutoffs indicated by dashed lines. A few genes of interest were annotated on the figure. (B) The heatmaps of 5-h and 24-h L. monocytogenes-infected HBCs from the same 3 placentas used in the experiment whose results are shown in Fig. 6. Left, heatmap of all DE genes based on scaled Log_2_ transcripts per million (TPM); right, heatmap of the top 50 protein-coding genes with the highest absolute Log_2_FC values in 24-h L. monocytogenes-infected versus 5-h L. monocytogenes-infected HBCs. Raw counts were normalized with edgeR and converted to TPM. The genes were clustered based on the CPM (counts per million); the same order is used in the TPM (transcripts per million), and the samples are in a set order. Red and blue indicate upregulated and downregulated genes in 24-h-infected cells, respectively. Download FIG S3, PDF file, 0.5 MB. Copyright © 2021 Azari et al. 2021 Azari et al. https://creativecommons.org/licenses/by/4.0/ This content is distributed under the terms of the Creative Commons Attribution 4.0 International license.

10.1128/mBio.01849-21.7 TABLE S1 Infection by L. monocytogenes upregulates the expression of chemokine-coding genes. HBCs were stimulated or not stimulated with IFN-γ/LPS for 24 h prior to L. monocytogenes infection. Control untreated HBCs (UT), HBCs treated with IFN-γ/LPS, and untreated L. monocytogenes-infected HBCs (*Lm*) were incubated for the indicated time points shown on the table (h). Data show the fold changes in expression of chemokine-coding genes (from RNA-seq). Upregulated (Log_2_FC > 1) and downregulated (Log_2_FC < −1) genes are highlighted in green and yellow, respectively, and significant FDR values are highlighted in pink. FC, fold change; FDR, false discovery rate. Download Table S1, DOCX file, 0.02 MB. Copyright © 2021 Azari et al. 2021 Azari et al. https://creativecommons.org/licenses/by/4.0/ This content is distributed under the terms of the Creative Commons Attribution 4.0 International license.

10.1128/mBio.01849-21.8 TABLE S2 Complete list of the Gene Ontology (GO) terms for the biological processes (BP) based on GOTERM_BP_DIRECT analysis using DAVID 6.8. The EASE score was set to 0.05, and the processes with an FDR of <0.05 were selected. The terms in bold were used in Fig. 6. Download Table S2, DOCX file, 0.03 MB. Copyright © 2021 Azari et al. 2021 Azari et al. https://creativecommons.org/licenses/by/4.0/ This content is distributed under the terms of the Creative Commons Attribution 4.0 International license.

**FIG 8:**
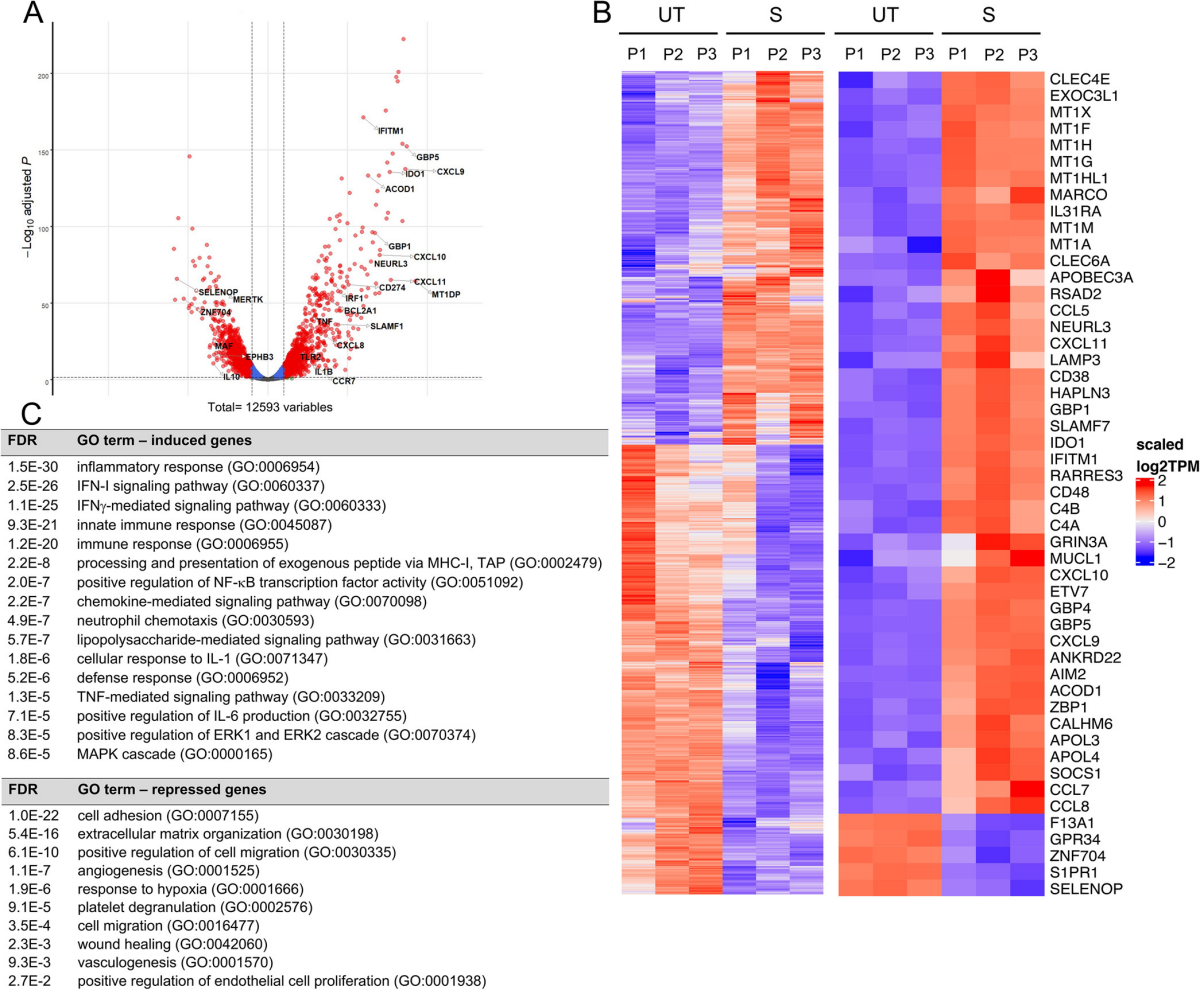
IFN-γ-/LPS-stimulated Hofbauer cells undergo proinflammatory transcriptional reprogramming. HBCs were either untreated or stimulated for 24 h with IFN-γ/LPS. Cells were washed and incubated for an additional 5 h. Cell culture supernatants were collected and stored. The total RNA was collected, sequenced, and analyzed (*n* = 3 placentas, P1 to P3). (A) Volcano plot of −log_10_ statistical significance (FDR [false discovery rate] adjusted *P* value) against Log_2_-fold change in IFN-γ-/LPS-stimulated versus untreated HBCs. Red dots correspond to differentially expressed (DE) genes with significant and greater than 2-fold changes (FDR-adjusted *P* < 0.05, Log_2_FC > 1 or <−1). Cutoffs are indicated by dashed lines. A few genes of interest were annotated on the figure. (B) Heatmaps of untreated (UT) and stimulated (S) HBCs from the same placentas as used in the experiment whose results are shown in Fig. 6. Left, heatmap of all DE genes based on scaled Log_2_ transcripts per million (TPM); right, heatmap of the top 50 protein-coding genes with the highest absolute Log_2_FC values in IFN-γ-/LPS-stimulated versus untreated HBCs. Raw counts were normalized with edgeR and converted to TPM. The genes were clustered based on the CPM (counts per million); the same order is used in the TPM, and the samples are in a set order. Red and blue indicate upregulated and downregulated genes, respectively, in stimulated cells. (C) Gene Ontology (GO) analysis of all DE genes using DAVID 6.8, with an EASE score of 0.05 and FDR of <0.05.

**FIG 9:**
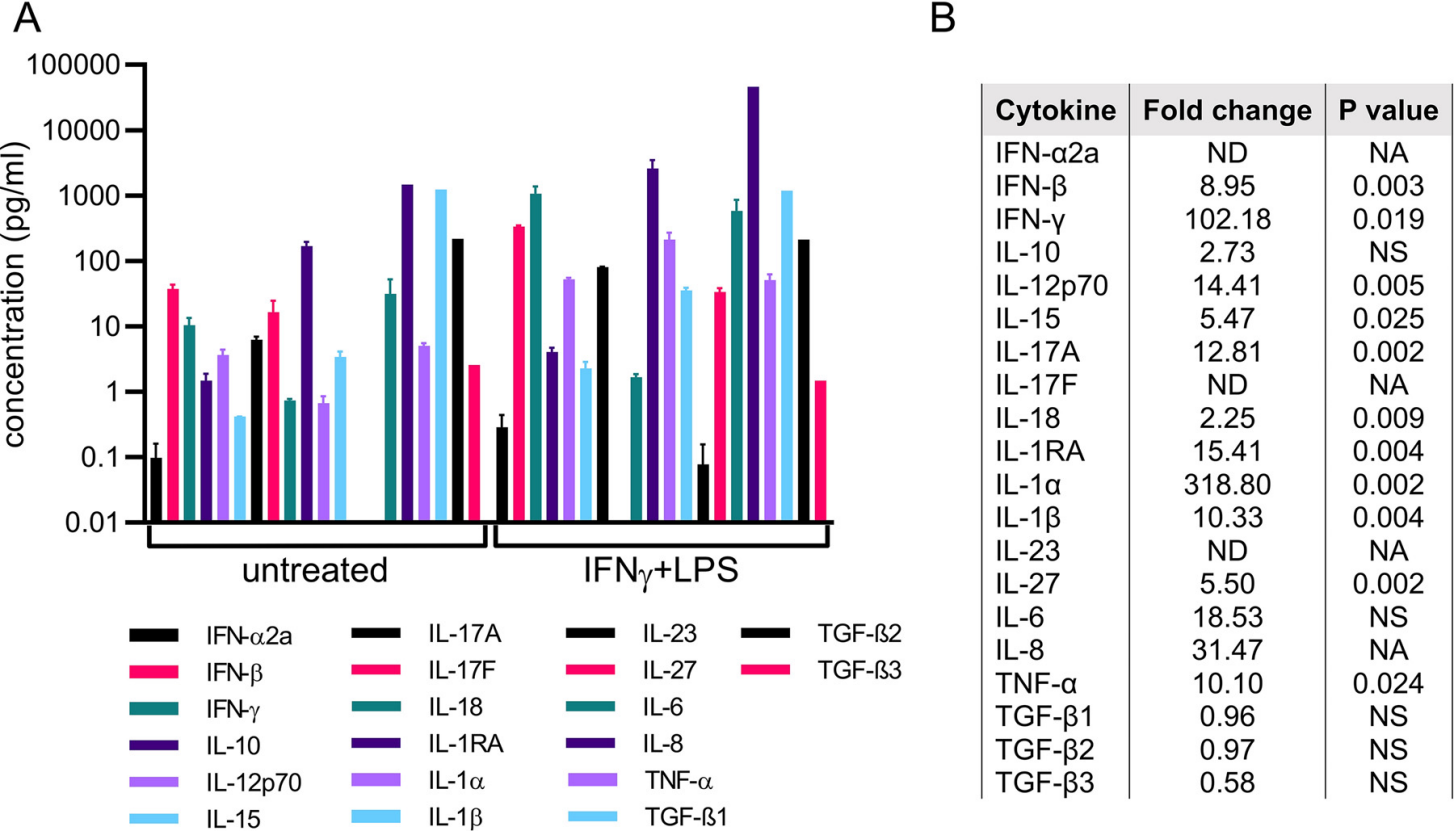
IFN-γ-/LPS-stimulated Hofbauer cells produce pro-inflammatory cytokines. HBCs were either untreated or stimulated for 24 h with IFN-γ/LPS. Cells were washed and incubated for an additional 5 h. The cell culture supernatants were collected and analyzed by using the Meso Scale Discovery (MSD) multiplex cytokine array. (A) Culture supernatants were analyzed in duplicate for each cytokine and experimental condition, and data are the average values for 20 measured cytokines (pg/ml ± SEM). *n* = 3, the same placentas as used in the experiment whose results are shown in Fig. 6. For IL-8 and TGF-β only, samples of the 3 experiments were pooled before measurements. (B) The fold change of each cytokine produced by stimulated HBCs in comparison to the untreated condition was expressed. Ratio paired Student’s *t* test was used to generate the *P* values. ND, not detected (below detection limit); NS, not significant; NA, not applicable (for the pooled samples).

In parallel experiments, we performed RNA-seq analysis of HBCs exposed to IFN-γ/LPS for 24 h in comparison to control untreated cells. Data showed that 983 (7.8%) genes were significantly induced and 1,183 (9.4%) were significantly repressed (Fig. 8A and B). The top 50 significantly DE protein-coding genes were largely dominated by immune genes (Fig. 8B). Gene Ontology analysis of DE genes revealed that the top upregulated pathways included IFN-γ and LPS signaling, confirming HBC responsiveness to these agents (Fig. 8C). Other upregulated pathways reflected the production of and responses to pro-inflammatory cytokines, immune cell trafficking, and immune defense pathways. These included critical immune signaling, such as MAPK and ERK1/ERK2 cascades, NF-κB, and key pro-inflammatory cytokines IFN-α/β, TNF-α, IL-1α/β, IL-6, IL-8, and IL-12. Confirming these pathways, IFN-α/β, TNF-α, IL-1α/β, IFN-γ, IL-6, IL-8, and IL-12 cytokine production were markedly increased (Fig. 9). Numerous chemokine genes were highly upregulated, including CXCL9, -10, -11, and -8 (Fig. 8A and B; Table S1). At the protein level, CXCL8 (IL-8) was the most abundantly produced cytokine at the basal level (in our array), and its production increased ∼30-fold upon stimulation. Other major upregulated pathways included antigen processing for MHC-I presentation, with upregulation of classical (HLA-A, -B, and -C) and non-classical (HLA-E, -F, and -G) HLA-I genes. Conversely, tissue-specific homeostatic pathways (mostly associated with the M2 phenotype) were downregulated, including extracellular matrix organization, angiogenesis, and wound healing (Fig. 8C).

In conclusion, HBCs can undergo pro-inflammatory reprogramming in response to L. monocytogenes infection and to exposure to potent M1-polarizing agents, as characterized by the upregulation of pro-inflammatory and antimicrobial transcriptional pathways, consistent with the increased production of pro-inflammatory cytokines and chemokines and enhanced L. monocytogenes infection control (Fig. 2 and 4).

### M1/M2 gene signature and cytokine analyses support that L. monocytogenes infection repolarizes HBCs toward an M1 phenotype.

**FIG 10:**
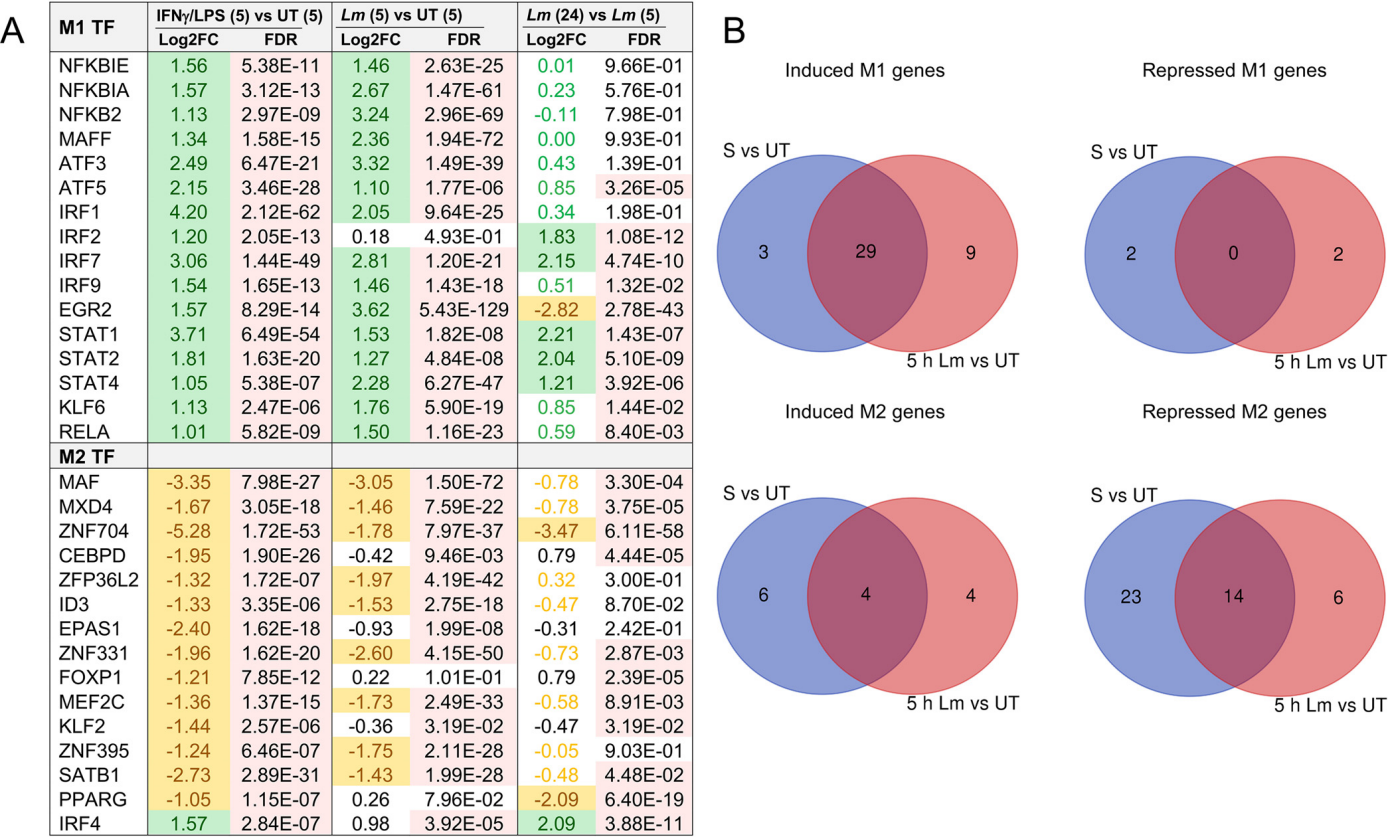
Transcriptional remodeling of M1 and M2 signature transcription factor (TF) genes in IFN-γ-/LPS-stimulated and L. monocytogenes-infected HBCs. HBCs were either untreated or stimulated for 24 h with IFN-γ/LPS. Cells (from the same placentas used in the experiment whose results are shown in Fig. 6) were washed and infected or not infected with WT L. monocytogenes. After 5 and 24 h, RNA was collected, sequenced, and analyzed. RNA-seq data of untreated (UT), IFN-γ-/LPS-stimulated (S), and L. monocytogenes-infected (*Lm*) HBCs were analyzed for expression of M1- and M2-specific genes. (A) Significantly (Log_2_FC > 1 or < −1, FDR < 0.05) induced (highlighted green) and repressed (highlighted in yellow) M1/M2 transcription regulator genes at the indicated time points on the table in panel A (h) are shown. FC, fold change; FDR, false discovery rate. (B) Venn diagrams of induced or repressed (differentially expressed [DE]) M1 and M2 genes. The corresponding complete list of M1- and M2-specific genes is available in Table S3.

The transcriptional remodeling of infected or IFN-γ-/LPS-stimulated HBCs reflected an M1 polarization. To further establish whether HBCs could repolarize, we next analyzed DE M1 and M2 signature genes 5 h and 24 h after L. monocytogenes infection and compared the results with 24-h IFN-γ-/LPS-treated HBCs. Based on the literature, we generated a list of 75 M1 and 80 M2 gene markers, which encode transcription factors, cytokines/chemokines, immune receptors, immune transducers, and antimicrobial and pro- and anti-inflammatory molecules (Table S3). HBC infection or stimulation with IFN-γ/LPS for 24 h led to the predominant upregulation of M1 genes, while M2 genes were downregulated (Table S3). Strikingly, M1- and M2-specific transcription regulators were upregulated and downregulated, respectively, in infected HBCs and IFN-γ-/LPS-stimulated HBCs, similar to a previous report of M1-polarized blood-derived human macrophages (Fig. 10A) (48). The Venn diagrams in Fig. 10B showed 38 M1 genes were upregulated 5 h post-infection, including 29 genes shared with IFN-γ-/LPS-stimulated HBCs. Twenty M2 genes were downregulated 5 h post-infection, including 14 genes shared with IFN-γ-/LPS-stimulated HBCs, and 15 additional M2 genes were downregulated 24 h post-infection (Fig. 10B; Table S3). Interestingly, a feature of HBCs infected by L. monocytogenes for 24 h but not observed in IFN-γ-/LPS-stimulated HBCs was the downregulation of 13 MHC-II genes (M1 markers), as previously reported as an L. monocytogenes virulence mechanism in non-placental macrophages (Table S3) (49, 50). Our data further support that this is due to the downregulation of the MHC-II transcriptional coactivator CIITA (Table S3) (51).

10.1128/mBio.01849-21.9 TABLE S3 Expression of M1 and M2 signature genes by HBCs. HBCs were stimulated or not stimulated with IFN-γ/LPS for 24 h prior to L. monocytogenes infection. Control untreated HBCs (UT), HBCs treated with IFN-γ/LPS, and untreated L. monocytogenes-infected HBCs (*Lm*) were incubated for the indicated times shown on the table (h). Data show the fold changes in expression of M1 and M2 signature genes (from RNA-seq). Upregulated (Log_2_FC > 1) and downregulated (Log_2_FC < −1) genes are highlighted in green and yellow, respectively, and significant FDR values are highlighted in pink. FC, fold change; FDR, false discovery rate. Download Table S3, DOCX file, 0.04 MB. Copyright © 2021 Azari et al. 2021 Azari et al. https://creativecommons.org/licenses/by/4.0/ This content is distributed under the terms of the Creative Commons Attribution 4.0 International license.

The profile of cytokine production by L. monocytogenes-infected (IFN- α/β, IL-1 α/β, IL-18, IL-6, IL-8, TNF-α, IL-12, and IL-23) and IFN-γ-/LPS-stimulated (IL-1α, IFN-γ, IL-6, IL-8, TNF-α, IL-12, and IFN-β) HBCs supported their M1 repolarization (Fig. 7 and 9). In particular, the increase in the pro-inflammatory cytokines IFN-α, TNF-α, IL-6, IL-1β, and IL-18 was markedly more robust in infected HBCs than in IFN-γ-/LPS-stimulated HBCs (Fig. 7 and 9). Also, IL-23, a proinflammatory cytokine that belongs to the IL-12 family, was highly upregulated upon infection (1,537-fold increase 24 h post-infection) but undetected following stimulation by IFN-γ/LPS (52). However, the production of the active form of IL-12 (an M1 cytokine) was significantly increased and the IL-12/IL-10 ratio (12.9) was characteristic of an M1 phenotype in stimulated HBCs (47). HBCs produce the IL-1 receptor antagonist (IL-1RA), which inhibits IL-1α/β signaling when in large excess (molar ratio of at least ∼10 to 100) (53). The IL-1RA/IL-1 ratio decreased from 28.3 in untreated and uninfected cells to 9 in 24-h-infected cells and to 10.5 in 24-h IFN-γ-/LPS-stimulated cells (54).

Another indication of macrophage repolarization is their metabolic shift, with M1 macrophages relying more on glycolysis and accumulating itaconate, whereas M2 macrophages rely on oxidative phosphorylation and have an intact tricarboxylic acid (TCA) cycle (55). Two major metabolic genes involved in M1 polarization were significantly upregulated 5 and 24 h post-infection, the PFKFB3 gene, which stimulates glycolysis, and the ACOD1 gene, involved in itaconate production (Fig. 6A; Table S4) (55, 56). In parallel, genes supporting fatty acid synthesis and oxidation, the FASN (57) and HADH (58) gene, respectively, were downregulated (Fig. 6D; Table S4). Fatty acids are required for the TCA cycle in M2 macrophages (55, 59). Other downregulated genes involved in the TCA cycle were the genes coding for pyruvate carboxylase (PC), an enzyme involved in the production of oxaloacetate from pyruvate, and lactate dehydrogenase (LDHD) and ME1, both involved in the production of pyruvate (Table S4) (60). Downregulation of these genes and pathways in L. monocytogenes-infected HBCs suggests a metabolic shift toward an M1-like phenotype by disruption of M2-specific metabolic pathways. In comparison, only ACOD1 was strongly induced in IFN-γ-/LPS-stimulated HBCs (Table S4).

10.1128/mBio.01849-21.10 TABLE S4 Effect of L. monocytogenes infection and IFN-γ/LPS stimulation on M1 and M2 metabolic genes. Control untreated HBCs (UT), HBCs treated with IFN-γ/LPS, and untreated L. monocytogenes-infected HBCs (*Lm*) were incubated for the indicated times shown on the table. Data show the fold changes in expression of a collection of metabolic genes (from RNA-seq). Differentially expressed (DE) upregulated (Log_2_FC > 1) genes are highlighted in green, downregulated genes (Log_2_FC < −1) are highlighted in yellow, and significant FDR values are highlighted in pink. FC, fold change; FDR, false discovery rate. Download Table S4, DOCX file, 0.02 MB. Copyright © 2021 Azari et al. 2021 Azari et al. https://creativecommons.org/licenses/by/4.0/ This content is distributed under the terms of the Creative Commons Attribution 4.0 International license.

As an alternative approach to assess macrophage repolarization, we measured the surface expression of canonical M1/M2 markers by quantitative fluorescence microscopy (Fig. S4). This approach was favored over flow cytometry, which would have altered surface markers while detaching these strongly adherent cells. We found similar results for infected and IFN-γ-/LPS-stimulated HBCs: CD163 (M2 marker) decreased only slightly, CD86 (M1 and M2b markers) increased slightly, and the expression of CD80 (M1 marker) and CD14/CD206/CD209 (M2 markers) remained minimally affected (Fig. S4).

10.1128/mBio.01849-21.4 FIG S4 M1/M2 marker expression by L. monocytogenes-infected and/or IFN-γ-/LPS-stimulated HBCs. HBCs were either untreated or stimulated for 24 h with IFN-γ/LPS. Cells were washed and infected or not infected with WT L. monocytogenes for 5 h and 24 h. At the indicated time points shown on the figure, cells were PFA fixed and labeled with primary Abs against M1/M2 markers (or with control isotypes), followed by Alexa Fluor 488-conjugated secondary Abs and DAPI labeling (to enumerate cells). Fluorescence images were randomly acquired, corresponding to ∼500 HBCs per experimental condition. The data are expressed as the average fluorescence intensity per cell in arbitrary units (AFI) ± SEM (calculated from a minimum of 11 planes). Presented are the data for one representative experiment of 3 independent experiments (3 placentas). Download FIG S4, PDF file, 0.03 MB. Copyright © 2021 Azari et al. 2021 Azari et al. https://creativecommons.org/licenses/by/4.0/ This content is distributed under the terms of the Creative Commons Attribution 4.0 International license.

In conclusion, both transcriptional reprogramming and cytokine profiling support an M1 repolarization of HBCs upon infection by L. monocytogenes and stimulation by IFN-γ/LPS. Surprisingly, the expression of some M2 surface markers decreased only slightly and the CD80 M1 surface marker did not increase upon either infection or IFN-γ/LPS stimulation.

### L. monocytogenes-infected HBCs display a tolerogenic signature.

The M1-specific T cell co-stimulatory molecule CD80 remained undetected on L. monocytogenes-infected HBCs, which, together with the downregulation of MHC-II expression, is unlikely to favor CD4^+^ T cell activation (Table 1; Fig. S4). Also, some of the few upregulated M2-associated genes in infected HBCs are known to promote fetal immune tolerance, including the nonclassical HLA class I molecule HLA-G (Table 1) (22, 53, 61–66). Therefore, we next focused on analyzing the expression of tolerogenic factors that could suppress T cell activation during L. monocytogenes infection (Table 1). Several upregulated transcripts encoding cell surface receptors (CD274, PDCD1, and LILRB1, -2, and -3) are known to prevent T cell activation (Table 1) (67–70). The indoleamine 2,3-dioxygenase 1 (IDO1)-coding gene, which exerts antiviral and anti-L. monocytogenes activities and immunosuppressive properties contributing to fetal tolerance, as well as T cell suppressive mediators IL-10 and vascular endothelial growth factor C (VEGFC), were upregulated in L. monocytogenes-infected HBCs (Table 1) (65, 71–77). Finally, the transcripts encoding regulatory T cell (Treg) chemokines (CCL1 and CCL22) and the retinoic acid-producing enzyme ALDH1A2, known to promote Treg development, were upregulated in L. monocytogenes-infected HBCs (Table 1) (78, 79). Additionally, the cytokine array showed that L. monocytogenes infection led to an increase of up to 70-fold in IL-10 production, while IL-12 increased only weakly, which does not favor Th1 development (Fig. 7). The production of TGF-β remained high and was unaffected at the mRNA (not shown) and protein levels post-infection (Fig. 7). Overall, HBCs appear to balance their pro-inflammatory responses to L. monocytogenes with the maintenance or induction of tolerogenic signals, likely as an attempt to maintain fetal tolerance. This property of activated HBCs is likely not limited to L. monocytogenes-infected HBCs, since several of these tolerogenic factors were similarly modulated in IFN-γ-/LPS-stimulated cells (Table 1).

**TABLE 1:**
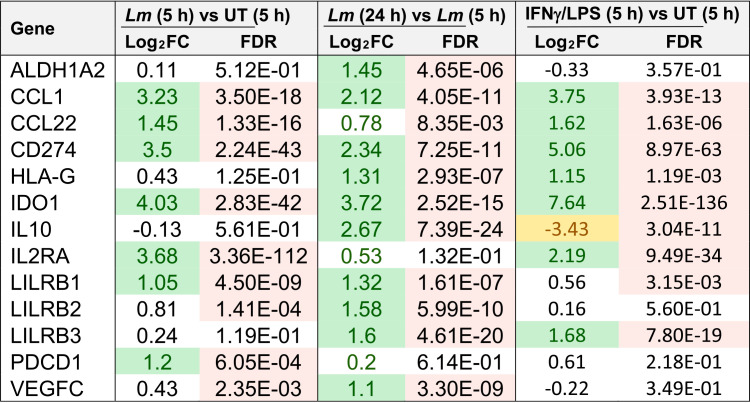
L. monocytogenes infection and IFN-γ/LPS stimulation upregulate tolerogenic genes^*a*^

a Control untreated HBCs (UT), HBCs treated with IFN-γ/LPS, and untreated L. monocytogenes-infected HBCs (*Lm*) were incubated for the indicated times shown on the table (h). Data show the fold changes in expression (from RNA-seq) of a collection of genes known to promote T cell tolerance. Differentially expressed (DE) upregulated (Log_2_FC > 1) genes are highlighted in green, DE downregulated genes (Log_2_FC < −1) are highlighted in yellow, and significant FDR values are highlighted in pink. FC, fold change; FDR, false discovery rate.

## DISCUSSION

Although previous studies have been devoted to establishing how L. monocytogenes can breach the placental barrier, no studies addressed the fate of L. monocytogenes within the placenta nor its mechanism of transmission to the fetus. In particular, whether or not the fetal, placenta-resident macrophages, Hofbauer cells, can control L. monocytogenes infection was unknown. In the present study, we showed that, at the basal state, HBCs killed a large proportion of phagocytosed non-pathogenic bacterial species, such as L. innocua, and displayed relatively low susceptibility to infection by L. monocytogenes. However, *L monocytogenes* could exploit HBCs to maintain an intracellular niche and infect surrounding placental cells. Furthermore, the data support that HBCs underwent pro-inflammatory reprograming toward an M1 phenotype upon L. monocytogenes infection, leading to the production of major pro-inflammatory cytokines. However, HBCs upregulated the expression of key tolerogenic genes and maintained the production of tolerogenic cytokines, in accordance with their role in preventing the activation of maternal anti-fetal adaptive immune responses.

HBC phenotype and plasticity are still poorly characterized despite the important roles these cells play in placental homeostasis. We found that 98% of HBCs express the M2-specific surface marker CD163, as previously described (24, 26, 29, 30). At least 75% of HBCs co-express markers that are traditionally attributed to distinct M2 subclasses, including CD206 (M2b), CD14 (M2c), and CD86 (M1 or M2b), indicating that the typical M2 classification may not apply to these cells (24, 26, 29, 47, 80–82). In their basal state, HBCs mainly produce the cytokines IL-8 and TGF-β1, followed by TGF-β2 and IL-1RA. The production of TGF-β1/β2 and IL-1RA is in accordance with HBCs’ M2 homeostatic and tolerogenic activities (26, 30, 83, 84). In agreement with our findings, third-trimester HBCs produce high levels of IL-8 (29, 30, 85, 86) and were identified, together with trophoblasts, as responsible for the constitutive production of IL-8 in the human placenta (122). Thus, under our experimental conditions, HBCs retain their *in vivo* profile of cytokine production.

Common placental complications of infectious and non-infectious etiologies, including villitis, chorioamnionitis, miscarriage, and preterm delivery, are associated with abnormal placental inflammation, and the contribution of HBCs to inflammation has been debated (3, 17, 88, 89). Some studies support that HBCs maintain an M2 anti-inflammatory profile in cases of gestational diabetes mellitus (GDM) and villitis (24, 25, 29). Other studies support a pro-inflammatory role for HBCs in GDM, villitis of unknown etiology (VUE), and during infection (30, 90–93). A recent study proposed that, despite stimulation by IFN-γ/LPS for 96 h, HBCs resisted M1 repolarization and displayed an M2b phenotype (29). Our data strongly support the plasticity of infected or IFN-γ-/LPS-stimulated HBCs, as characterized by their transcriptional reprogramming toward an M1 pro-inflammatory phenotype, accompanied by the production of pro-inflammatory cytokines and chemokines. In accordance with HBCs’ plasticity are the findings of a previous study that compared the DNA methylomes of HBCs, decidual macrophages (placental macrophages of maternal origin), and fetal and maternal blood monocytes (94). That study found that HBCs’ M1 and immune-response-related genes were hypermethylated, whereas M2 genes were hypo-methylated, consistent with the HBC’s anti-inflammatory and homeostatic functions at basal levels. Of interest, DNA methyl transferases DNMT3A and DNMT3B (which promote *de novo* methylation) were highly expressed by HBCs in comparison to the levels of maternal monocyte/macrophages, suggesting that HBCs are prone to epigenetic remodeling (94). Our data support that HBCs can undergo M1-like reprogramming. Indeed, numerous M2 transcripts, including the transcription factor (TF) MAF, which assembles enhancers for sustained M2 gene transcription, were downregulated in stimulated and in infected HBCs, similarly to M1-polarized adult blood-derived macrophages (48). Reciprocally, numerous M1 genes were upregulated, including the transcription factor IRF1, which assembles enhancers for M1 gene transcription (48). In addition, the transcriptome of L. monocytogenes-infected HBCs reflected increased glycolysis and itaconate production (an M1 signature), whereas several M2 metabolic pathways involving fatty acids and pyruvate were downregulated (Fig. 6). Alternatively, fatty acids are required for bacterial membrane synthesis (95); therefore, downregulation of fatty acid synthesis could be a strategy used by HBCs to limit bacterial proliferation. Gene Ontology and KEGG pathway analyses remarkably recapitulated the detection of extracellular and intracellular Gram-positive organisms by pattern recognition receptors that leads to transcriptional activation of numerous pro-inflammatory cytokines and chemokines and their downstream signaling pathways (Fig. 6C and D). The cytokine array of L. monocytogenes-infected HBCs confirmed the production of many of these pro-inflammatory cytokines (IL-8, TNF-α, IL-6, IL-23, IFN-α/β, IFN-γ, IL-12, IL-17A, and IL-1α), including the inflammasome-/caspase-1-dependent cytokines IL-1β and IL-18 (Fig. 7) (46).

L. monocytogenes-infected HBCs undergo unique M1-like reprogramming, which could be described as M1t (tolerogenic), for the maintenance of tolerogenic factors associated with fetal tolerance. Some tolerogenic properties were also observed in IFN-γ-/LPS-stimulated HBCs, suggesting that HBCs may be programmed to maintain fetal tolerance despite various pro-inflammatory stimulations (Table 1). HBCs maintained or increased the expression of genes encoding chemokines, surface receptors, and secreted factors that favor the recruitment and development of regulatory T cells (Table 1), and they failed to express the co-stimulatory molecule CD80 (Fig. S4). Thus, we propose that HBCs are programmed to respond to L. monocytogenes infection by promoting an environment favorable to the innate immune responses to infection, while attempting to prevent deleterious adaptive anti-fetal immunity, at least during the first 24 h of exposure to the pathogen. It should also be considered that L. monocytogenes may induce some of the tolerogenic responses as a virulence mechanism, as illustrated by the downregulation of the MHC-II gene transcriptional coactivator CIITA and numerous MHC-II-coding genes (49). The tolerogenic properties of HBCs may constitute the HBCs’ Achilles heel during infection, as they can potentially lead to antibacterial T cell response suppression and may facilitate the establishment of L. monocytogenes in the placenta and subsequent spread to the fetus.

The literature reports that different types of macrophages display a wide range of susceptibility to L. monocytogenes infection. Murine M2-polarized liver-resident Kupffer cells are highly susceptible to and rapidly die from L. monocytogenes infection (96). We found that pro-survival and apoptotic pathways were both upregulated in infected HBCs. However, pro-survival pathways likely dominated, since within 24 h of infection, there were no apoptotic nuclei (DAPI [4′,6-diamidino-2-phenylindole] labeling) and no detectable cell detachment or death of infected HBCs, unlike THP-1 cells, which detached massively 24 h post-infection (data not shown). Human blood-derived macrophages and murine peritoneal macrophages can kill L. monocytogenes, and more so after IFN-γ, TNF-α, or granulocyte-macrophage colony-stimulating factor (GM-CSF) stimulation (97, 98). Stimulation of murine bone marrow-derived macrophages by IFN-γ/LPS enhances L. monocytogenes control while decreasing phagocytic efficiency but does not induce net bacterial killing (31, 99, 100). We found that HBCs restrict L. monocytogenes infection, although not completely, as a proportion of cytosolic L. monocytogenes cells remain viable, motile, and able to divide (Fig. 3C and 5C and D). However, the net number of viable intracellular bacteria did not increase between 5 and 7.5 h post-infection (Fig. 2D), supporting that intracellular killing of L. monocytogenes compensated for its cytosolic multiplication. Consistent with the notion that HBCs display some antimicrobial properties and are able to kill phagocytosed bacteria, we showed that a significant proportion (∼40%) of phagocytosed L. innocua cells (non-pathogenic) were killed as early as 5 h post-infection (Fig. 2F), in accordance with a recent study showing that first-trimester HBCs were bactericidal against nonpathogenic bacterial species (101). We found that ∼11% of intracellular L. monocytogenes cells remained trapped in LAMP-1-positive vacuoles 2 h post-infection, which increased to 20% in stimulated HBCs (Fig. 3A and B), consistent with the notion that HBCs may successfully kill a small proportion of phagosomal L. monocytogenes. We also found that in stimulated HBCs, the bacterial cytosolic division time increased while the proportion of motile bacteria decreased 4 to 6 h post-infection (Fig. 3C and 5D). These data suggest a model in which non-motile bacteria might be targeted by autophagy, since motility is critical to escape from autophagy (102). The M1-like pro-inflammatory reprogramming of HBCs observed 5 h post-infection is consistent with the fact that the net number of viable intracellular bacteria stopped increasing between 5 and 7.5 h post-infection, indicating the activation of some antimicrobial mechanisms. Together, these data indicate that HBCs display some antimicrobial properties at the phagosomal and cytosolic levels at basal state and have the intrinsic ability to further increase their activities if properly stimulated. However, these antimicrobial functions are not sufficient to avert L. monocytogenes intracellular survival.

Macrophages have been shown to spread L. monocytogenes infection to other cell types, including endothelial cells (41, 103, 104), and L. monocytogenes cell-to-cell spread is required for infection of the placental/fetal unit (11, 12, 45). Based on their presence at the placental/fetal interface, their phagocytic ability, and their motility, HBCs are the best candidates for fetal transmission of L. monocytogenes. In support of this, we found that HBCs transfer L. monocytogenes cells to other primary placental cells, including fetal endothelial cells (Fig. 5A and B; Fig. S2). Furthermore, HBCs may play a general role in spreading infection to the fetus, as recently proposed for the Zika virus (105–108). During infection, tissue-resident macrophages typically release chemokines to attract inflammatory monocytes, neutrophils, and other leukocytes for subsequent clearance of the pathogens (96, 109). We observed that L. monocytogenes-infected HBCs upregulate numerous transcripts for chemokines that attract neutrophils, monocytes/macrophages, and T cells, which was confirmed at the protein level with IL-8. The pro-inflammatory profile of infected HBCs is in accordance with the villitis observed in animal and human listeriosis cases (11, 27, 28). Villitis can lead to infiltration of maternal leukocytes, as evidenced by trafficking of CD8^+^ cytotoxic T cells in villitis of unknown etiology (VUE) (92, 110). VUE is also accompanied by increased levels of CXCL9, -10, and -11 (CXCR3 ligands) in HBCs and can lead to fetal death and other complications (111). Similarly, we observed induction of gene expression for these chemokines in L. monocytogenes-infected HBCs. Therefore, it is likely that if infection of chorionic villi is not resolved, exacerbation of inflammation by HBCs and infiltration of maternal leukocytes may contribute to poor outcomes, such as abortion and preterm birth, observed in listeriosis cases in pregnancy (6).

## MATERIALS AND METHODS

### Bacterial strains and culture.

Wild-type (WT) L. monocytogenes strain 10403S and its isogenic Δ*hly* and Δ*actA* mutants were from Daniel Portnoy (University of California, Berkeley). The isogenic red fluorescent L. monocytogenes (p*actA*-RFP) was from Anna Bakardjiev (University of California, San Francisco) (10). L. innocua (strain BUG 499) was from Pascale Cossart (Pasteur Institute, Paris). Bacteria were grown overnight in antibiotic-free brain heart infusion (BHI) broth in a shaking incubator at 37°C until reaching an optical density at 600 nm (OD_600_) of 0.7 to 0.8, and were diluted to the indicated multiplicity of infection (MOI) in serum-/antibiotic-free cell culture medium as shown under the corresponding figures.

### HBC isolation and characterization.

Placentas (a total of 18 placentas) were obtained from healthy, singleton Cesarean section 38-week deliveries at the Ohio State University Maternity Center (IRB#2017H0478). Tissue was dissected within 15 min of placental collection, as described by Kliman et al. (112), which included complete removal of maternal membranes and the decidua. The scraped villous trees were digested, and Hofbauer cells (HBCs) were isolated using a discontinuous Percoll plus (GE Healthcare) gradient according to Tang et al. without negative immunoselection (37). The cells were incubated overnight at 0.5 × 10^6^/ml in isolation RPMI (RPMI containing 25 mM HEPES [Gibco] and 5% heat-inactivated fetal bovine serum [FBSH]) in Teflon wells (Savillex) at 37°C and 5% CO_2_. The following morning, cells were passed through a 40-μm sieve, centrifuged, and suspended at 10^6^/ml in isolation RPMI. HBCs were isolated based on their ability to attach to the cell culture plate for 30 min at 37°C, whereas non-adherent chorionic cells were removed by washes. The cells were cultured with Dulbecco’s modified Eagle’s medium/nutrient mixture F-12 (1:1) (DMEM/F-12 GlutaMax; Gibco) containing 10% FBSH, 50 U/ml penicillin, and 50 μg/ml streptomycin (Invitrogen) (complete DMEM/F-12). All HBCs were cultured for 48 h and then exposed to IFN-γ/LPS for 24 h (in parallel, untreated HBCs were cultured for 24 h in the absence of IFN-γ/LPS). Subsequently, the stimulated and untreated cells were infected, so that all infection experiments described in this article were carried out 72 h after plating of HBCs (29, 37). For purity assessment, untreated cells were fixed (72 h postplating) with 4% paraformaldehyde (PFA) and labeled with antibodies (Abs) against CD45 (clone HI30; abcam), CD163 (clone GHI/61; Thermo Fisher Scientific), CD90 (clone 5E10; Biolegend), and cytokeratin 7 (ab53123, polyclonal Ab; abcam), with secondary fluorescent Abs, and with DAPI to label nuclei. For cell purity, fluorescence images were randomly acquired with a 40× objective (at least 750 cells per experiment). The total number of cells (*N_n_*), based on DAPI labeling, and the number of cells positive for each marker (*N_m_*) were enumerated. The percent positivity for each marker was expressed as (*N_m_*/*N_n_*) × 100 ± SEM (*n* = 7 placentas). HBC integrity was assessed by incubation for 2 min at 37°C in culture medium containing 100 μM propidium iodide (PI; Sigma-Aldrich). Cells were washed with phosphate-buffered saline (PBS) and fixed with 4% PFA for 15 min at room temperature (RT), followed by DAPI labeling. Cell integrity was assessed based on PI fluorescence intensity in the nuclear regions (DAPI positive).

### Trophoblast, HUVEC, and THP-1 cell culture.

Primary human trophoblasts (PHTs) were isolated (from the same placenta as HBCs for each experiment) as previously described (112) and cultured in complete DMEM/F-12. Human umbilical vein endothelial cells (HUVECs) were from ScienCell Research Laboratories and cultured as previously described (113). THP-1 cells (ATCC TIB-202; authenticated by ATCC’s short tandem repeat profiling) were cultured in RPMI 1640 GlutaMax containing 10% FBSH, 50 U/ml penicillin, and 50 μg/ml streptomycin (complete RPMI) and differentiated into adherent cells using 160 nM phorbol myristate acetate (PMA) 48 h prior to infection (36). THP-1 cells (treated with PMA for 24 h) and HBCs (cultured for 48 h) were treated for 24 h with lipopolysaccharide (LPS from Escherichia coli O55:B5; Sigma-Aldrich) and IFN-γ (recombinant; R&D systems) at 100 ng/ml and 100 U/ml (5 ng/ml), respectively (31, 36).

### Gentamicin survival assay.

HBCs (0.6 × 10^6^ cells/well) and THP-1 cells (0.5 × 10^6^ cells/well) were plated, in duplicate, in 24-well tissue culture plates (Falcon) and infected at 1 < MOI < 2 for L. monocytogenes and L. innocua (for all infections in this study unless otherwise indicated in the figure legends). Cells were infected for 30 min, followed by washes and treatment with 15 μg/ml gentamicin for the indicated times shown on the figures. For bacterial enumeration, cells were washed with PBS and lysed in water/0.2% Triton X-100. Cell lysates were diluted and plated on BHI/agar plates. Data were expressed as the number of CFU per cell ± SEM (*n* = 3 placentas or 3 independent THP-1 cell experiments).

### Measuring macrophage infection by fluorescence microscopy.

Infected HBCs were fixed in 4% PFA at the indicated time points shown on the figures and labeled with DAPI and mouse anti-CD45 Abs and secondary Abs, and extracellular bacteria versus total bacteria were labeled as previously described (113). A minimum of 15 fluorescence images were randomly acquired in each experimental condition in duplicates (more than 700 cells per condition). To calculate phagocytosis, the numbers of total bacteria (*N_t_*), extracellular bacteria (*N_e_*), and CD45^+^ cells (*N*_CD45_) were enumerated. Entry was expressed as the number of intracellular bacteria per cell as follows: (*N_t_* − *N_e_*)/*N*_CD45_ ± SEM (*n* = 3 placentas). To calculate the percentages of infected HBCs, the number of infected CD45^+^ cells (*N*_CD45i_) and the total number of CD45-positive cells (*N_n_*) were enumerated, and the percentage of infected cells was measured at each time point as follows: (*N*_CD45i_/*N_n_*) × 100 ± SEM (*n* = 3, the same placentas as were used for entry).

### LAMP-1 and F-actin labeling.

HBCs were stimulated or not with IFN-γ/LPS for 24 h and washed and infected with WT L. monocytogenes. Two hours post-infection, cells were fixed with 4% PFA and labeled with A488-conjugated phalloidin (Molecular Probes) or anti-LAMP-1 Ab (clone H4A3; Developmental Studies Hybridoma Bank) for 1 h at RT. Extracellular and total bacteria were labeled with anti-L. monocytogenes antibodies (113). Fluorescence images were acquired, and the percentages of LAMP-1^+^ and F-actin^+^ intracellular L. monocytogenes cells were counted from at least 19 planes (corresponding to a minimum of 500 bacteria) per experimental condition and are expressed as fold change in comparison to untreated HBCs ± SEM (*n* = 3 placentas).

### Cell-to-cell-spreading assay.

HUVECs (8 × 10^3^ cells/well) and trophoblasts (7.5 × 10^5^ cells/well) were cultured in duplicate in 24-well plates to reach 90 to 100% confluence after 72 h of culture. HBCs (2 × 10^6^ cells/well) were seeded in 6-well tissue culture plates for 72 h. HBCs were infected with WT or Δ*actA* L. monocytogenes at an MOI of 10 for 1 h, washed with DMEM/F-12 GlutaMax, and incubated in infection DMEM/F-12 (DMEM/F-12 containing 15 μg/ml gentamicin). Five hours post-infection, HBCs were detached using Cellstripper reagent containing 15 μg/ml gentamicin according to the manufacturer’s protocol. Five-hundred-microliter amounts of HBCs (3 × 10^4^) in the medium of the target cell type were transferred onto each well of trophoblasts or HUVECs at similar concentrations per cell surface area to correct for the difference in the sizes of each cell type. The plates were centrifuged at 360 × *g* for 3 min at RT and were incubated at 37°C for 1 h. Cells were washed and incubated with FBSH- and gentamicin-containing medium at 37°C for 18 h. After fixation with 4% PFA, HBCs were labeled with anti-CD14 antibodies conjugated to Alexa Fluor 647 (clone 63D3; Biolegend), HUVECs with Alexa Fluor 488 phalloidin (Molecular Probes), and trophoblasts with anti-E-cadherin Ab (clone HECD-1; abcam) (secondary antibodies were conjugated to Alexa Fluor 488). Following permeabilization with 0.2% Triton X-100, bacteria were labeled with anti-L. monocytogenes antibodies and secondary anti-rabbit antibody Alexa Fluor 568. Nuclei were labeled with DAPI. A minimum of 20 phase-contrast and fluorescence images (22,550 and 840 nuclei for trophoblasts and HUVECs, respectively) were randomly acquired with the 20× objective from two wells per condition. For trophoblasts and HUVECs, the number of the infectious foci, the area of the infectious foci (*A_i_*), and the total cell surface area (*A_t_*) in each plane were measured using the Metamorph analysis software (Molecular Devices). The results were averaged, and the area covered by foci was expressed as follows: (*A_i_*/*A_t_*) × 100 ± SEM.

### Quantitative fluorescence imaging of HBC surface markers.

For quantification of fluorescence intensities, 10 sets of phase-contrast and fluorescence images were randomly acquired for each experimental condition, using the 40× objective (at least 500 cells per experimental condition). The fluorescence background was subtracted, and a threshold was applied to only measure the cell-associated fluorescence. The number of nuclei (*N_n_*) and the integrated fluorescence intensity (*I_f_*) were automatically measured using Metamorph software. Fluorescence intensity per cell was calculated using the formula (*I_f_*/*N_n_*) in each plane, and the results were averaged. Data were shown as average fluorescence intensity (AFI)/nucleus ± SEM (Fig. S4 shows a representative experiment of *n* = 3 independent experiments from 3 placentas). The antibodies were against CD80 (clone 2D10; Biolegend), CD86 (clone IT2.2; Biolegend), CD163 (clone GHI/61; Thermo Fisher Scientific), CD206 (clone 15-2; Biolegend), CD209 (clone 9E9A8; Biolegend), and CD14 (clone 63D3; Biolegend).

### Live-cell imaging.

HBCs were seeded (3 × 10^6^) in glass bottom dishes (P35G-1.5-10-C; MatTek). Cells were infected with p*actA*-RFP bacteria (MOI of 5) for 30 min, washed, and incubated in infection DMEM/F-12. Four hours post-infection, cell culture medium was replaced with DMEM/F-12 without phenol red (Gibco) containing 25 mM HEPES and 15 μg/ml gentamicin (cell-imaging medium). Three movies were acquired, each corresponding to a different placenta (*n* = 3 placentas; these 3 placentas were used for all live-cell-imaging experiments explained in this section), with an average of 100 macrophages in each plane per placenta. Z series of fluorescence images were acquired every 1 min for 120 min using a 40× oil objective. The percentages of non-motile bacteria were enumerated by counting bacteria that remained in a perimeter of 2 μm for 10 min (after flattening the images using the Z Projection function in MetaMorph). A total of nine 10-min segments from the beginning, middle, and end of the 2-h movies were analyzed, the percentage of non-motile bacteria in each segment was enumerated, and the results were averaged. To measure bacterial speed, images were flattened, and bacterial trajectories were tracked using the Multi-Line function of the MetaMorph analysis software. The speeds (*s*) of at least 20 individual bacteria per experiment were measured as *s* = *d*/*t*, where *d* is the distance traveled in μm and *t* the elapsed time in min. To calculate the bacterial division time, phase-contrast and fluorescence images were acquired from 10 different memorized positions, and images were reacquired 2 h later at the same positions (*n* = 3 individual experiments from the above-mentioned 3 placentas), 10 planes per experiment. The numbers of red-fluorescent cytosolic bacteria before (*B*) and after (*b*) and the elapsed times (*t*) were measured, and the division (generation) time (*G*) was expressed as follows: *G* = *t*/[3.3 × log(*b*/*B*)] ± SEM (114). Minimums of 129 and 576 bacteria (per experiment) were counted before and after infection, respectively.

### Explant immunofluorescence.

Placental explants were obtained and cultured as described previously (115). The villi were sectioned and labeled according to the protocol by Ganesan et al. (116), with primary antibodies (rabbit anti-C7 Ab, mouse anti-CD14 Ab, and rabbit anti-vimentin Ab [clone SP20; abcam] and mouse anti-CD34 Ab [clone QBEND/10; Thermo Fisher]) followed by incubation with secondary Alexa Fluor antibodies. The nuclei were labeled with DAPI.

### Cytokine array to determine the inflammatory profile of HBCs.

Cell culture supernatants were collected in duplicates and centrifuged for 20 min at 4°C at 14,000 rpm. Meso Scale Discovery (MSD) cytokine arrays were performed in duplicate using supernatants diluted 1:2 with the cell culture medium and using the Meso QuickPlex SQ 120 device. Each cytokine concentration was established based on standard curves obtained from the calibrators provided.

### RNA sequencing, generation of differentially expressed gene lists, and data analysis.

Cells obtained from 3 placentas (the same ones used for cytokine arrays) were lysed in TRIzol reagent (Invitrogen). The PureLink RNA extraction kit with on-column DNase treatment (Invitrogen) was used to isolate high-quality RNA (extracted separately for each of *n* = 3 experiments). Input RNA quality and quantity was assessed using the Agilent 2100 Bioanalyzer (Agilent Technologies, Santa Clara, CA) and Qubit fluorometer (Thermo Fisher Scientific), respectively. The RNA integrity number values were greater than 7 and the RNA concentration was greater than 100 ng/μl for all samples. Total RNA-seq libraries were generated with the NEBNext ultra II directional RNA library prep kit for Illumina (catalog number E7760L; NEB) and NEBNext rRNA depletion kit with sample purification beads (catalog number E6350; NEB), with an input amount of 200 ng total RNA per sample. Libraries were pooled and sequenced on an Illumina NovaSeq SP flowcell in paired-end 150-bp format (Illumina, San Diego, CA) to a read yield of between 70 and 80 million reads (equivalent to 35 to 40 million clusters). Raw fastq was aligned to human reference genome GRCh38 with hisat2 version 2.1.0 (117). Alignment QC was assessed by using RSeQC. Gene-wise counts were generated with featureCounts from the subread package version1.5.1 for genes annotated by Ensembl GRCh38.92 (118). An average of 46.6 million pairs of reads were sequenced per sample over two lanes with a 96.2% average overall alignment rate. An average of 89.0% of reads were uniquely mapped, and 76.1% of mapped reads aligned to known exonic regions (coding sequences [CDSs] and untranslated regions [UTRs]). Ribosomal contamination was 0.44%. Counts from separate sequencing lanes for the same sample were summed. Count normalization and differential expression were performed with edgeR using a negative binomial distribution with a generalized linear model (GLM) with a paired design (119). Genes were tested if at least half of the samples had an expression of 2 CPM (counts per million). Genes with an absolute Log_2_-fold change (Log_2_FC) of >1 with a false discovery rate (FDR) of <0.05 were considered significant and are referred to as differentially expressed (DE) genes. R packages ComplexHeatmap and EnhancedVolcano were used for the heatmaps and volcano plots, respectively. The Gene Ontology (GO) analyses were performed using DAVID 6.8 (120, 121). Upregulated and downregulated DE genes were uploaded separately as induced and repressed gene lists, respectively. The program uses a list of DE genes and does not use Log_2_FC or FDR. For the biological processes (BP), the GOTERM_BP_DIRECT terms were used by setting the EASE score at 0.05 and selecting the pathways with an FDR of <0.05. The same criteria were used to select for the KEGG pathways. The pathways were manually grouped and color-coded based on their functional categories. The Venn diagrams were drawn using the online tool at http://bioinformatics.psb.ugent.be/webtools/Venn/.

### Statistical analyses.

Summary statistics, i.e., mean values ± SEM, are shown in the figures. All data were the average values of at least 3 independent experiments, corresponding to at least 3 different placentas. All graphs and statistical analyses were generated using GraphPad Prism 7 and 8. For independent data, Student’s *t* tests were used for analysis of two-group comparisons. Analysis of variance (ANOVA) or a mixed-effect model was used for analysis of multiple-group comparisons. Tukey’s or Sidak’s method was used to control for multiple comparisons when needed. A *P* value of <0.05 after adjustment for multiple comparisons was considered statistically significant. *P* values and statistical methods used for each experiment are indicated in the corresponding figure legends.

### Microscopy equipment.

The motorized, atmosphere-controlled, inverted wide-field fluorescence microscope equipment was previously described, with the addition of a 40× LD Plan Neofluar (numeric aperture [NA] = 0.6) objective (Carl Zeiss) (113).

### Data availability.

The RNA-seq raw data and the Log_2_ transcripts per million (TPM) table are available under GEO accession number GSE174689.

## References

[1] Robbins JR, Bakardjiev AI . 2012. Pathogens and the placental fortress. Curr Opin Microbiol 15:36–43. doi:10.1016/j.mib.2011.11.006. 22169833 PMC3265690

[2] Erlebacher A, Vencato D, Price KA, Zhang D, Glimcher LH . 2007. Constraints in antigen presentation severely restrict T cell recognition of the allogeneic fetus. J Clin Invest 117:1399–1411. doi:10.1172/JCI28214. 17446933 PMC1849983

[3] Zulu MZ, Martinez FO, Gordon S, Gray CM . 2019. The elusive role of placental macrophages: the Hofbauer cell. J Innate Immun 11:447–456. doi:10.1159/000497416. 30970346 PMC6758944

[4] Arora N, Sadovsky Y, Dermody TS, Coyne CB . 2017. Microbial vertical transmission during human pregnancy. Cell Host Microbe 21:561–567. doi:10.1016/j.chom.2017.04.007. 28494237 PMC6148370

[5] Lamond N, Freitag N . 2018. Vertical transmission of *Listeria monocytogenes*: probing the balance between protection from pathogens and fetal tolerance. Pathogens 7:52. doi:10.3390/pathogens7020052. PMC6027155 29799503

[6] Charlier C, Disson O, Lecuit M . 2020. Maternal-neonatal listeriosis. Virulence 11:391–397. doi:10.1080/21505594.2020.1759287. 32363991 PMC7199740

[7] Vazquez-Boland JA, Krypotou E, Scortti M . 2017. Listeria placental infection. mBio 8:e00949-17. doi:10.1128/mBio.00949-17. 28655824 PMC5487735

[8] Mayhew TM, Simpson RA . 1994. Quantitative evidence for the spatial dispersal of trophoblast nuclei in human placental villi during gestation. Placenta 15:837–844. doi:10.1016/S0143-4004(05)80185-7. 7886024

[9] Wang Y, Zhao S . 2010. Vascular biology of the placenta, p 3–11. Morgan & Claypool Life Sciences, San Rafael, CA. 21452443

[10] Zeldovich VB, Robbins JR, Kapidzic M, Lauer P, Bakardjiev AI . 2011. Invasive extravillous trophoblasts restrict intracellular growth and spread of *Listeria monocytogenes*. PLoS Pathog 7:e1002005. doi:10.1371/journal.ppat.1002005. 21408203 PMC3048367

[11] Lecuit M, Nelson DM, Smith SD, Khun H, Huerre M, Vacher-Lavenu M-C, Gordon JI, Cossart P . 2004. Targeting and crossing of the human maternofetal barrier by *Listeria monocytogenes*: role of internalin interaction with trophoblast E-cadherin. Proc Natl Acad Sci U S A 101:6152–6157. doi:10.1073/pnas.0401434101. 15073336 PMC395938

[12] Bakardjiev AI, Stacy BA, Portnoy DA . 2005. Growth of *Listeria monocytogenes* in the guinea pig placenta and role of cell-to-cell spread in fetal infection. J Infect Dis 191:1889–1897. doi:10.1086/430090. 15871123

[13] Wolfe B, Wiepz GJ, Schotzko M, Bondarenko GI, Durning M, Simmons HA, Mejia A, Faith NG, Sampene E, Suresh M, Kathariou S, Czuprynski CJ, Golos TG . 2017. Acute fetal demise with first trimester maternal infection resulting from *Listeria monocytogenes* in a nonhuman primate model. mBio 8:e01938-16. doi:10.1128/mBio.01938-16. 28223455 PMC5358912

[14] Benirschke K, Burton GJ, Baergen RN . 2012. Pathology of the human placenta, p 55–100. SpringerVerlag, Berlin, Germany.

[15] Aplin JD, Lewis RM, Jones CJP . 2018. Development of the human placental villus. Reference module in biomedical sciences. Elsevier, Amsterdam, The Netherlands.

[16] Castellucci M, Zaccheo D, Pescetto G . 1980. A three-dimensional study of the normal human placental villous core. I. The Hofbauer cells. Cell Tissue Res 210:235–247. doi:10.1007/BF00237612. 7407868

[17] Reyes L, Golos TG . 2018. Hofbauer cells: their role in healthy and complicated pregnancy. Front Immunol 9:2628. doi:10.3389/fimmu.2018.02628. 30498493 PMC6249321

[18] Reyes L, Wolfe B, Golos T . 2017. Hofbauer cells: placental macrophages of fetal origin. Results Probl Cell Differ 62:45–60. doi:10.1007/978-3-319-54090-0_3. 28455705

[19] Russell DG, Huang L, VanderVen BC . 2019. Immunometabolism at the interface between macrophages and pathogens. Nat Rev Immunol 19:291–304. doi:10.1038/s41577-019-0124-9. 30679807 PMC7032560

[20] Stocks CJ, Schembri MA, Sweet MJ, Kapetanovic R . 2018. For when bacterial infections persist: Toll-like receptor-inducible direct antimicrobial pathways in macrophages. J Leukoc Biol 103:35–51. doi:10.1002/JLB.4RI0917-358R. 29345056

[21] Seveau S, Pizarro-Cerda J, Cossart P . 2007. Molecular mechanisms exploited by *Listeria monocytogenes* during host cell invasion. Microbes Infect 9:1167–1175. doi:10.1016/j.micinf.2007.05.004. 17761447

[22] Mosser DM, Edwards JP . 2008. Exploring the full spectrum of macrophage activation. Nat Rev Immunol 8:958–969. doi:10.1038/nri2448. 19029990 PMC2724991

[23] Shaughnessy LM, Swanson JA . 2007. The role of the activated macrophage in clearing *Listeria monocytogenes* infection. Front Biosci 12:2683–2692. doi:10.2741/2364. 17127272 PMC2851543

[24] Schliefsteiner C, Peinhaupt M, Kopp S, Lögl J, Lang-Olip I, Hiden U, Heinemann A, Desoye G, Wadsack C . 2017. Human placental Hofbauer cells maintain an anti-inflammatory M2 phenotype despite the presence of gestational diabetes mellitus. Front Immunol 8:888. doi:10.3389/fimmu.2017.00888. 28824621 PMC5534476

[25] Joerink M, Rindsjö E, van Riel B, Alm J, Papadogiannakis N . 2011. Placental macrophage (Hofbauer cell) polarization is independent of maternal allergen-sensitization and presence of chorioamnionitis. Placenta 32:380–385. doi:10.1016/j.placenta.2011.02.003. 21419483

[26] Loegl J, Hiden U, Nussbaumer E, Schliefsteiner C, Cvitic S, Lang I, Wadsack C, Huppertz B, Desoye G . 2016. Hofbauer cells of M2a, M2b and M2c polarization may regulate feto-placental angiogenesis. Reproduction 152:447–455. doi:10.1530/REP-16-0159. 27534571

[27] Abram M, Dorić M . 1997. Primary *Listeria monocytogenes* infection in gestating mice. Folia Microbiol (Praha) 42:65–71. doi:10.1007/BF02898648. 9161004

[28] Parkash V, Morotti RA, Joshi V, Cartun R, Rauch CA, West AB . 1998. Immunohistochemical detection of Listeria antigens in the placenta in perinatal listeriosis. Int J Gynecol Pathol 17:343–350. doi:10.1097/00004347-199810000-00008. 9785135

[29] Schliefsteiner C, Ibesich S, Wadsack C . 2020. Placental Hofbauer cell polarization resists inflammatory cues *in vitro*. Int J Mol Sci 21:736. doi:10.3390/ijms21030736. PMC7038058 31979196

[30] Young OM, Tang Z, Niven-Fairchild T, Tadesse S, Krikun G, Norwitz ER, Mor G, Abrahams VM, Guller S . 2015. Toll-like receptor-mediated responses by placental Hofbauer cells (HBCs): a potential pro-inflammatory role for fetal M2 macrophages. Am J Reprod Immunol 73:22–35. doi:10.1111/aji.12336. 25345551 PMC4268350

[31] Tsang AW, Oestergaard K, Myers JT, Swanson JA . 2000. Altered membrane trafficking in activated bone marrow-derived macrophages. J Leukoc Biol 68:487–494. 11037969

[32] Martinez FO, Sica A, Mantovani A, Locati M . 2008. Macrophage activation and polarization. Front Biosci 13:453–461. doi:10.2741/2692. 17981560

[33] Genin M, Clement F, Fattaccioli A, Raes M, Michiels C . 2015. M1 and M2 macrophages derived from THP-1 cells differentially modulate the response of cancer cells to etoposide. BMC Cancer 15:577. doi:10.1186/s12885-015-1546-9. 26253167 PMC4545815

[34] Ouadrhiri Y, Scorneaux B, Sibille Y, Tulkens PM . 1999. Mechanism of the intracellular killing and modulation of antibiotic susceptibility of *Listeria monocytogenes* in THP-1 macrophages activated by gamma interferon. Antimicrob Agents Chemother 43:1242–1251. doi:10.1128/AAC.43.5.1242. 10223943 PMC89140

[35] Conte MP, Petrone G, Di Biase AM, Longhi C, Penta M, Tinari A, Superti F, Fabozzi G, Visca P, Seganti L . 2002. Effect of acid adaptation on the fate of *Listeria monocytogenes* in THP-1 human macrophages activated by gamma interferon. Infect Immun 70:4369–4378. doi:10.1128/IAI.70.8.4369-4378.2002. 12117947 PMC128136

[36] Scorneaux B, Ouadrhiri Y, Anzalone G, Tulkens PM . 1996. Effect of recombinant human gamma interferon on intracellular activities of antibiotics against *Listeria monocytogenes* in the human macrophage cell line THP-1. Antimicrob Agents Chemother 40:1225–1230. doi:10.1128/AAC.40.5.1225. 8723471 PMC163296

[37] Tang Z, Tadesse S, Norwitz E, Mor G, Abrahams VM, Guller S . 2011. Isolation of Hofbauer cells from human term placentas with high yield and purity. Am J Reprod Immunol 66:336–348. doi:10.1111/j.1600-0897.2011.01006.x. 21545365 PMC3154981

[38] Chanput W, Mes JJ, Wichers HJ . 2014. THP-1 cell line: an *in vitro* cell model for immune modulation approach. Int Immunopharmacol 23:37–45. doi:10.1016/j.intimp.2014.08.002. 25130606

[39] Auwerx J . 1991. The human leukemia cell line, THP-1: a multifacetted model for the study of monocyte-macrophage differentiation. Experientia 47:22–31. doi:10.1007/BF02041244. 1999239

[40] Gedde MM, Higgins DE, Tilney LG, Portnoy DA . 2000. Role of listeriolysin O in cell-to-cell spread of *Listeria monocytogenes*. Infect Immun 68:999–1003. doi:10.1128/IAI.68.2.999-1003.2000. 10639481 PMC97240

[41] Portnoy DA, Auerbuch V, Glomski IJ . 2002. The cell biology of *Listeria monocytogenes* infection: the intersection of bacterial pathogenesis and cell-mediated immunity. J Cell Biol 158:409–414. doi:10.1083/jcb.200205009. 12163465 PMC2173830

[42] Vázquez-Boland JA, Kuhn M, Berche P, Chakraborty T, Domínguez-Bernal G, Goebel W, González-Zorn B, Wehland J, Kreft J . 2001. Listeria pathogenesis and molecular virulence determinants. Clin Microbiol Rev 14:584–640. doi:10.1128/CMR.14.3.584-640.2001. 11432815 PMC88991

[43] Tilney LG, Portnoy DA . 1989. Actin filaments and the growth, movement, and spread of the intracellular bacterial parasite, *Listeria monocytogenes*. J Cell Biol 109:1597–1608. doi:10.1083/jcb.109.4.1597. 2507553 PMC2115783

[44] Kocks C, Gouin E, Tabouret M, Berche P, Ohayon H, Cossart P . 1992. *L. monocytogenes*-induced actin assembly requires the actA gene product, a surface protein. Cell 68:521–531. doi:10.1016/0092-8674(92)90188-I. 1739966

[45] Le Monnier A, Autret N, Join-Lambert OF, Jaubert F, Charbit A, Berche P, Kayal S . 2007. ActA is required for crossing of the fetoplacental barrier by *Listeria monocytogenes*. Infect Immun 75:950–957. doi:10.1128/IAI.01570-06. 17118980 PMC1828513

[46] Abrahams VM, Tang Z, Mor G, Guller S . 2020. NLRP3 inflammasome function and pyroptotic cell death in human placental Hofbauer cells. J Reprod Immunol 142:103214. doi:10.1016/j.jri.2020.103214. 33152658 PMC7770077

[47] Mantovani A, Sica A, Sozzani S, Allavena P, Vecchi A, Locati M . 2004. The chemokine system in diverse forms of macrophage activation and polarization. Trends Immunol 25:677–686. doi:10.1016/j.it.2004.09.015. 15530839

[48] Kang K, Park SH, Chen J, Qiao Y, Giannopoulou E, Berg K, Hanidu A, Li J, Nabozny G, Kang K, Park-Min K-H, Ivashkiv LB . 2017. Interferon-γ represses M2 gene expression in human macrophages by disassembling enhancers bound by the transcription factor MAF. Immunity 47:235–250.e4. doi:10.1016/j.immuni.2017.07.017. 28813657 PMC5568089

[49] Schüller S, Kügler S, Goebel W . 1998. Suppression of major histocompatibility complex class I and class II gene expression in *Listeria monocytogenes*-infected murine macrophages. FEMS Immunol Med Microbiol 20:289–299. doi:10.1016/S0928-8244(98)00024-8. 9626934

[50] Webster P . 2002. Early intracellular events during internalization of *Listeria monocytogenes* by J774 cells. J Histochem Cytochem 50:503–517. doi:10.1177/002215540205000407. 11897803

[51] Masternak K, Muhlethaler-Mottet A, Villard J, Zufferey M, Steimle V, Reith W . 2000. CIITA is a transcriptional coactivator that is recruited to MHC class II promoters by multiple synergistic interactions with an enhanceosome complex. Genes Dev 14:1156–1166. 10809673 PMC316580

[52] Floss DM, Moll JM, Scheller J . 2020. IL-12 and IL-23—close relatives with structural homologies but distinct immunological functions. Cells 9:2184. doi:10.3390/cells9102184. PMC7600943 32998371

[53] Pollard JW, Bartocci A, Arceci R, Orlofsky A, Ladner MB, Stanley ER . 1987. Apparent role of the macrophage growth factor, CSF-1, in placental development. Nature 330:484–486. doi:10.1038/330484a0. 2446141

[54] Gouze J-N, Gouze E, Palmer GD, Liew VS, Pascher A, Betz OB, Thornhill TS, Evans CH, Grodzinsky AJ, Ghivizzani SC . 2003. A comparative study of the inhibitory effects of interleukin-1 receptor antagonist following administration as a recombinant protein or by gene transfer. Arthritis Res Ther 5:R301–R309. doi:10.1186/ar795. 12932294 PMC193732

[55] Viola A, Munari F, Sánchez-Rodríguez R, Scolaro T, Castegna A . 2019. The metabolic signature of macrophage responses. Front Immunol 10:1462. doi:10.3389/fimmu.2019.01462. 31333642 PMC6618143

[56] Galván-Peña S, O’Neill LAJ . 2014. Metabolic reprograming in macrophage polarization. Front Immunol 5:420. doi:10.3389/fimmu.2014.00420. 25228902 PMC4151090

[57] Wakil SJ, Stoops JK, Joshi VC . 1983. Fatty acid synthesis and its regulation. Annu Rev Biochem 52:537–579. doi:10.1146/annurev.bi.52.070183.002541. 6137188

[58] Yang SY, He XY, Schulz H . 2005. 3-Hydroxyacyl-CoA dehydrogenase and short chain 3-hydroxyacyl-CoA dehydrogenase in human health and disease. FEBS J 272:4874–4883. doi:10.1111/j.1742-4658.2005.04911.x. 16176262

[59] Orecchioni M, Ghosheh Y, Pramod AB, Ley K . 2019. Macrophage polarization: different gene signatures in M1 (LPS^+^) vs. classically and M2 (LPS–) vs. alternatively activated macrophages. Front Immunol 10:1084. doi:10.3389/fimmu.2019.01084. 31178859 PMC6543837

[60] Murai S, Ando A, Ebara S, Hirayama M, Satomi Y, Hara T . 2017. Inhibition of malic enzyme 1 disrupts cellular metabolism and leads to vulnerability in cancer cells in glucose-restricted conditions. Oncogenesis 6:e329. doi:10.1038/oncsis.2017.34. 28481367 PMC5523067

[61] Zhang Y-H, He M, Wang Y, Liao A-H . 2017. Modulators of the balance between M1 and M2 macrophages during pregnancy. Front Immunol 8:120. doi:10.3389/fimmu.2017.00120. 28232836 PMC5299000

[62] Wang D, Yang L, Yue D, Cao L, Li L, Wang D, Ping Y, Shen Z, Zheng Y, Wang L, Zhang Y . 2019. Macrophage-derived CCL22 promotes an immunosuppressive tumor microenvironment via IL-8 in malignant pleural effusion. Cancer Lett 452:244–253. doi:10.1016/j.canlet.2019.03.040. 30928379

[63] Ferreira LMR, Meissner TB, Tilburgs T, Strominger JL . 2017. HLA-G: at the interface of maternal–fetal tolerance. Trends Immunol 38:272–286. doi:10.1016/j.it.2017.01.009. 28279591

[64] Coulomb-L’Hermine A . 2007. Expression of interleukin-27 by human trophoblast cells. Placenta 28:1133–1140. doi:10.1016/j.placenta.2007.06.004. 17659773

[65] Munn DH, Zhou M, Attwood JT, Bondarev I, Conway SJ, Marshall B, Brown C, Mellor AL . 1998. Prevention of allogeneic fetal rejection by tryptophan catabolism. Science 281:1191–1193. doi:10.1126/science.281.5380.1191. 9712583

[66] Jørgensen N, Persson G, Hviid TVF . 2019. The tolerogenic function of regulatory T cells in pregnancy and cancer. Front Immunol 10:911. doi:10.3389/fimmu.2019.00911. 31134056 PMC6517506

[67] Yeboah M, Papagregoriou C, Jones DC, Chan HTC, Hu G, McPartlan JS, Schiött T, Mattson U, Mockridge CI, Tornberg U-C, Hambe B, Ljungars A, Mattsson M, Tews I, Glennie MJ, Thirdborough SM, Trowsdale J, Frendeus B, Chen J, Cragg MS, Roghanian A . 2020. LILRB3 (ILT5) is a myeloid cell checkpoint that elicits profound immunomodulation. JCI Insight 5:e141593. doi:10.1172/jci.insight.141593. PMC7526549 32870822

[68] Apps R, Gardner L, Sharkey AM, Holmes N, Moffett A . 2007. A homodimeric complex of HLA-G on normal trophoblast cells modulates antigen-presenting cells via LILRB1. Eur J Immunol 37:1924–1937. doi:10.1002/eji.200737089. 17549736 PMC2699429

[69] Ozen M, Novak C, Burd I . 2018. Placenta immune infiltrates and perinatal outcomes. Am J Reprod Immunol 79:e12850. doi:10.1111/aji.12850. 29577494

[70] Keir ME, Francisco LM, Sharpe AH . 2007. PD-1 and its ligands in T-cell immunity. Curr Opin Immunol 19:309–314. doi:10.1016/j.coi.2007.04.012. 17433872

[71] Nino-Castro A, Abdullah Z, Popov A, Thabet Y, Beyer M, Knolle P, Domann E, Chakraborty T, Schmidt SV, Schultze JL . 2014. The IDO1-induced kynurenines play a major role in the antimicrobial effect of human myeloid cells against *Listeria monocytogenes*. Innate Immunity 20:401–411. doi:10.1177/1753425913496442. 23940074

[72] Muraille E, Leo O, Moser M . 2014. TH1/TH2 paradigm extended: macrophage polarization as an unappreciated pathogen-driven escape mechanism? Front Immunol 5:603. doi:10.3389/fimmu.2014.00603. 25505468 PMC4244692

[73] Kamimura S, Eguchi K, Yonezawa M, Sekiba K . 1991. Localization and developmental change of indoleamine 2, 3-dioxygenase activity in the human placenta. Acta Med Okayama 45:135–139. doi:10.18926/AMO/32206. 1716396

[74] Terness P, Bauer TM, Röse L, Dufter C, Watzlik A, Simon H, Opelz G . 2002. Inhibition of allogeneic T cell proliferation by indoleamine 2,3-dioxygenase-expressing dendritic cells: mediation of suppression by tryptophan metabolites. J Exp Med 196:447–457. doi:10.1084/jem.20020052. 12186837 PMC2196057

[75] Fallarino F, Grohmann U, Vacca C, Bianchi R, Orabona C, Spreca A, Fioretti MC, Puccetti P . 2002. T cell apoptosis by tryptophan catabolism. Cell Death Differ 9:1069–1077. doi:10.1038/sj.cdd.4401073. 12232795

[76] Arango Duque G, Descoteaux A . 2014. Macrophage cytokines: involvement in immunity and infectious diseases. Front Immunol 5:491. doi:10.3389/fimmu.2014.00491. 25339958 PMC4188125

[77] Kalkunte SS, Mselle TF, Norris WE, Wira CR, Sentman CL, Sharma S . 2009. Vascular endothelial growth factor C facilitates immune tolerance and endovascular activity of human uterine NK cells at the maternal-fetal interface. J Immunol 182:4085–4092. doi:10.4049/jimmunol.0803769. 19299706 PMC3616376

[78] Liu Z-M, Wang K-P, Ma J, Guo Zheng S . 2015. The role of all-trans retinoic acid in the biology of Foxp3^+^ regulatory T cells. Cell Mol Immunol 12:553–557. doi:10.1038/cmi.2014.133. 25640656 PMC4579645

[79] Zaman TS, Arimochi H, Maruyama S, Ishifune C, Tsukumo S-I, Kitamura A, Yasutomo K . 2017. Notch balances Th17 and induced regulatory T cell functions in dendritic cells by regulating Aldh1a2 expression. J Immunol 199:1989–1997. doi:10.4049/jimmunol.1700645. 28779023

[80] Martinez FO, Gordon S . 2014. The M1 and M2 paradigm of macrophage activation: time for reassessment. F1000Prime Rep 6:13. doi:10.12703/P6-13. 24669294 PMC3944738

[81] Orme J, Mohan C . 2018. Macrophage subpopulations in systemic lupus erythematosus. Discov Med 13:151–158. 22369974

[82] Gensel JC, Zhang B . 2015. Macrophage activation and its role in repair and pathology after spinal cord injury. Brain Res 1619:1–11. doi:10.1016/j.brainres.2014.12.045. 25578260

[83] Akdis M, Aab A, Altunbulakli C, Azkur K, Costa RA, Crameri R, Duan S, Eiwegger T, Eljaszewicz A, Ferstl R, Frei R, Garbani M, Globinska A, Hess L, Huitema C, Kubo T, Komlosi Z, Konieczna P, Kovacs N, Kucuksezer UC, Meyer N, Morita H, Olzhausen J, O’Mahony L, Pezer M, Prati M, Rebane A, Rhyner C, Rinaldi A, Sokolowska M, Stanic B, Sugita K, Treis A, van de Veen W, Wanke K, Wawrzyniak M, Wawrzyniak P, Wirz OF, Zakzuk JS, Akdis CA . 2016. Interleukins (from IL-1 to IL-38), interferons, transforming growth factor β, and TNF-α: receptors, functions, and roles in diseases. J Allergy Clin Immunol 138:984–1010. doi:10.1016/j.jaci.2016.06.033. 27577879

[84] Svensson J, Jenmalm MC, Matussek A, Geffers R, Berg G, Ernerudh J . 2011. Macrophages at the fetal–maternal interface express markers of alternative activation and are induced by M-CSF and IL-10. J Immunol 187:3671–3682. doi:10.4049/jimmunol.1100130. 21890660

[85] Swieboda D , Johnson EL, Beaver J, Haddad L, Enninga EAL, Hathcock M, Cordes S, Jean V, Lane I, Skountzou I, Chakraborty R . 2020. Baby’s first macrophage: temporal regulation of Hofbauer cell phenotype influences ligand-mediated innate immune responses across gestation. J Immunol 204:2380–2391. doi:10.4049/jimmunol.1901185. 32213562 PMC7870092

[86] Pavlov OV, Selutin AV, Pavlova OM, Selkov SA . 2020. Two patterns of cytokine production by placental macrophages. Placenta 91:1–10. doi:10.1016/j.placenta.2020.01.005. 31941612

[87] Shimoya K, Matsuzaki N, Taniguchi T, Kameda T, Koyama M, Neki R, Saji F, Tanizawa O . 1992. Human placenta constitutively produces interleukin-8 during pregnancy and enhances its production in intrauterine infection. Biol Reprod 47:220–226. doi:10.1095/biolreprod47.2.220. 1391327

[88] Tang Z, Abrahams VM, Mor G, Guller S . 2011. Placental Hofbauer cells and complications of pregnancy. Ann N Y Acad Sci 1221:103–108. doi:10.1111/j.1749-6632.2010.05932.x. 21401637 PMC3707113

[89] Brien M-E, Baker B, Duval C, Gaudreault V, Jones RL, Girard S . 2019. Alarmins at the maternal–fetal interface: involvement of inflammation in placental dysfunction and pregnancy complications. Can J Physiol Pharmacol 97:206–212. doi:10.1139/cjpp-2018-0363. 30485131

[90] Sisino G, Bouckenooghe T, Aurientis S, Fontaine P, Storme L, Vambergue A . 2013. Diabetes during pregnancy influences Hofbauer cells, a subtype of placental macrophages, to acquire a pro-inflammatory phenotype. Biochim Biophys Acta 1832:1959–1968. doi:10.1016/j.bbadis.2013.07.009. 23872577

[91] Hendrix P, Tang Z, Silasi M, Racicot KE, Mor G, Abrahams VM, Guller S . 2020. Herpesvirus-infected Hofbauer cells activate endothelial cells through an IL-1β-dependent mechanism. Placenta 91:59–65. doi:10.1016/j.placenta.2020.01.010. 32174308 PMC7078070

[92] Kim J-S, Romero R, Kim MR, Kim YM, Friel L, Espinoza J, Kim CJ . 2008. Involvement of Hofbauer cells and maternal T cells in villitis of unknown aetiology. Histopathology 52:457–464. doi:10.1111/j.1365-2559.2008.02964.x. 18315598 PMC2896045

[93] Tamblyn JA, Lissauer DM, Powell R, Cox P, Kilby MD . 2013. The immunological basis of villitis of unknown etiology. Placenta 34:846–855. doi:10.1016/j.placenta.2013.07.002. 23891153

[94] Kim SY, Romero R, Tarca AL, Bhatti G, Kim CJ, Lee JHo, Elsey A, Than NG, Chaiworapongsa T, Hassan SS, Kang GH, Kim J-S . 2012. Methylome of fetal and maternal monocytes and macrophages at the feto-maternal interface. Am J Reprod Immunol 68:8–27. doi:10.1111/j.1600-0897.2012.01108.x. 22385097 PMC3479407

[95] Zheng CJ, Yoo J-S, Lee T-G, Cho H-Y, Kim Y-H, Kim W-G . 2005. Fatty acid synthesis is a target for antibacterial activity of unsaturated fatty acids. FEBS Lett 579:5157–5162. doi:10.1016/j.febslet.2005.08.028. 16146629

[96] Blériot C, Dupuis T, Jouvion G, Eberl G, Disson O, Lecuit M . 2015. Liver-resident macrophage necroptosis orchestrates type 1 microbicidal inflammation and type-2-mediated tissue repair during bacterial infection. Immunity 42:145–158. doi:10.1016/j.immuni.2014.12.020. 25577440

[97] Blauer F, Groscurth P, Schneemann M, Schoedon G, Schaffner A . 1995. Modulation of the antilisterial activity of human blood-derived macrophages by activating and deactivating cytokines. J Interferon Cytokine Res 15:105–114. doi:10.1089/jir.1995.15.105. 8590313

[98] Portnoy DA, Schreiber RD, Connelly P, Tilney LG . 1989. Gamma interferon limits access of *Listeria monocytogenes* to the macrophage cytoplasm. J Exp Med 170:2141–2146. doi:10.1084/jem.170.6.2141. 2511268 PMC2189551

[99] Portnoy DA, Jacks PS, Hinrichs DJ . 1988. Role of hemolysin for the intracellular growth of *Listeria monocytogenes*. J Exp Med 167:1459–1471. doi:10.1084/jem.167.4.1459. 2833557 PMC2188911

[100] Myers JT, Tsang AW, Swanson JA . 2003. Localized reactive oxygen and nitrogen intermediates inhibit escape of *Listeria monocytogenes* from vacuoles in activated macrophages. J Immunol 171:5447–5453. doi:10.4049/jimmunol.171.10.5447. 14607950 PMC2972186

[101] Thomas JR, Appios A, Zhao X, Dutkiewicz R, Donde M, Lee CYC, Naidu P, Lee C, Cerveira J, Liu B, Ginhoux F, Burton G, Hamilton RS, Moffett A, Sharkey A, McGovern N . 2021. Phenotypic and functional characterization of first-trimester human placental macrophages, Hofbauer cells. J Exp Med 218:e20200891. doi:10.1084/jem.20200891. 33075123 PMC7579740

[102] Cheng MI, Chen C, Engström P, Portnoy DA, Mitchell G . 2018. Actin-based motility allows *Listeria monocytogenes* to avoid autophagy in the macrophage cytosol. Cell Microbiol 20:e12854. doi:10.1111/cmi.12854. 29726107 PMC6113091

[103] Drevets DA, Sawyer RT, Potter TA, Campbell PA . 1995. *Listeria monocytogenes* infects human endothelial cells by two distinct mechanisms. Infect Immun 63:4268–4276. doi:10.1128/iai.63.11.4268-4276.1995. 7591057 PMC173606

[104] Dramsi S, Lévi S, Triller A, Cossart P . 1998. Entry of *Listeria monocytogenes* into neurons occurs by cell-to-cell spread: an *in vitro* study. Infect Immun 66:4461–4468. doi:10.1128/IAI.66.9.4461-4468.1998. 9712801 PMC108539

[105] Simoni MK, Jurado KA, Abrahams VM, Fikrig E, Guller S . 2017. Zika virus infection of Hofbauer cells. Am J Reprod Immunol 77:e12613. doi:10.1111/aji.12613. PMC5299062 27966815

[106] Quicke KM, Bowen JR, Johnson EL, McDonald CE, Ma H, O’Neal JT, Rajakumar A, Wrammert J, Rimawi BH, Pulendran B, Schinazi RF, Chakraborty R, Suthar MS . 2016. Zika virus infects human placental macrophages. Cell Host Microbe 20:83–90. doi:10.1016/j.chom.2016.05.015. 27247001 PMC5166429

[107] Schwartz DA . 2017. Viral infection, proliferation, and hyperplasia of Hofbauer cells and absence of inflammation characterize the placental pathology of fetuses with congenital Zika virus infection. Arch Gynecol Obstet 295:1361–1368. doi:10.1007/s00404-017-4361-5. 28396992 PMC5429341

[108] Tabata T, Petitt M, Puerta-Guardo H, Michlmayr D, Harris E, Pereira L . 2018. Zika virus replicates in proliferating cells in explants from first-trimester human placentas, potential sites for dissemination of infection. J Infect Dis 217:1202–1213. doi:10.1093/infdis/jix552. 29106643 PMC6075529

[109] Gregory SH, Sagnimeni AJ, Wing EJ . 1996. Bacteria in the bloodstream are trapped in the liver and killed by immigrating neutrophils. J Immunol 157:2514–2520. 8805652

[110] Kim CJ, Romero R, Chaemsaithong P, Kim J-S . 2015. Chronic inflammation of the placenta: definition, classification, pathogenesis, and clinical significance. Am J Obstet Gynecol 213:S53–S69. doi:10.1016/j.ajog.2015.08.041. 26428503 PMC4782598

[111] Kim MJ, Romero R, Kim CJ, Tarca AL, Chhauy S, LaJeunesse C, Lee D-C, Draghici S, Gotsch F, Kusanovic JP, Hassan SS, Kim J-S . 2009. Villitis of unknown etiology is associated with a distinct pattern of chemokine up-regulation in the feto-maternal and placental compartments: implications for conjoint maternal allograft rejection and maternal anti-fetal graft-versus-host disease. J Immunol 182:3919–3927. doi:10.4049/jimmunol.0803834. 19265171 PMC2754231

[112] Kliman HJ, Nestler JE, Sermasi E, Sanger JM, Strauss JF . 1986. Purification, characterization, and *in vitro* differentiation of cytotrophoblasts from human term placentae. Endocrinology 118:1567–1582. doi:10.1210/endo-118-4-1567. 3512258

[113] Phelps CC, Vadia S, Arnett E, Tan Y, Zhang X, Pathak-Sharma S, Gavrilin MA, Seveau S . 2018. Relative roles of listeriolysin O, InlA, and InlB in *L. monocytogenes* uptake by host cells. Infect Immun 86:e0555-18. doi:10.1128/IAI.00555-18. PMC6204736 30061379

[114] Zierdt CH, Swan JC . 1981. Generation time and growth rate of the human intestinal parasite Blastocystis hominis. J Protozool 28:483–485. doi:10.1111/j.1550-7408.1981.tb05324.x. 7198685

[115] Robbins JR, Skrzypczynska KM, Zeldovich VB, Kapidzic M, Bakardjiev AI . 2010. Placental syncytiotrophoblast constitutes a major barrier to vertical transmission of *Listeria monocytogenes*. PLoS Pathog 6:e1000732. doi:10.1371/journal.ppat.1000732. 20107601 PMC2809766

[116] Ganesan LP, Kim J, Wu Y, Mohanty S, Phillips GS, Birmingham DJ, Robinson JM, Anderson CL . 2012. FcγRIIb on liver sinusoidal endothelium clears small immune complexes. J Immunol 189:4981–4988. doi:10.4049/jimmunol.1202017. 23053513 PMC4381350

[117] Kim D, Langmead B, Salzberg SL . 2015. HISAT: a fast spliced aligner with low memory requirements. Nat Methods 12:357–360. doi:10.1038/nmeth.3317. 25751142 PMC4655817

[118] Liao Y, Smyth GK, Shi W . 2014. featureCounts: an efficient general purpose program for assigning sequence reads to genomic features. Bioinformatics 30:923–930. doi:10.1093/bioinformatics/btt656. 24227677

[119] Robinson MD, McCarthy DJ, Smyth GK . 2010. edgeR: a Bioconductor package for differential expression analysis of digital gene expression data. Bioinformatics 26:139–140. doi:10.1093/bioinformatics/btp616. 19910308 PMC2796818

[120] Huang DW, Sherman BT, Lempicki RA . 2009. Bioinformatics enrichment tools: paths toward the comprehensive functional analysis of large gene lists. Nucleic Acids Res 37:1–13. doi:10.1093/nar/gkn923. 19033363 PMC2615629

[121] Huang DW, Sherman BT, Lempicki RA . 2009. Systematic and integrative analysis of large gene lists using DAVID bioinformatics resources. Nat Protoc 4:44–57. doi:10.1038/nprot.2008.211. 19131956

[122] Johnson L, Azari S, Webb A, Zhang X, Gavrilin MA, Marshall J, Rood K, Seveau SM . 2021. Human placental trophoblasts infected by *Listeria monocytogenes* undergo a pro-inflammatory switch associated with poor pregnancy outcomes. Front Immunol 12:2926. doi:10.3389/fimmu.2021.709466. PMC8346206 34367171

