# Supplementary figures and images for: Hofbauer Cells Spread Listeria monocytogenes among Placental Cells and Undergo Pro-Inflammatory Reprogramming while Retaining Production of Tolerogenic Factors
Source: mBio. 2021 Aug 17;12(4):e01849-21. doi: 10.1128/mBio.01849-21 (PMC8406333; doi:10.1128/mBio.01849-21)

A

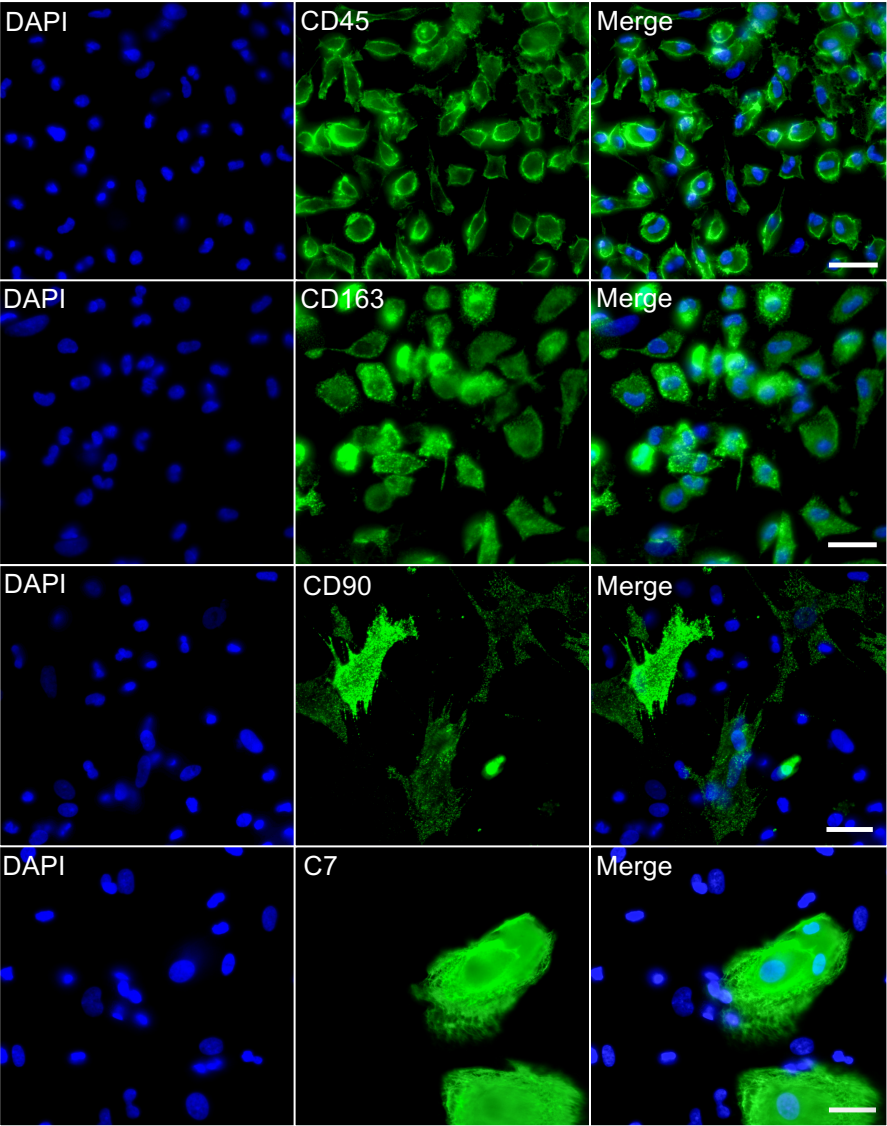

B

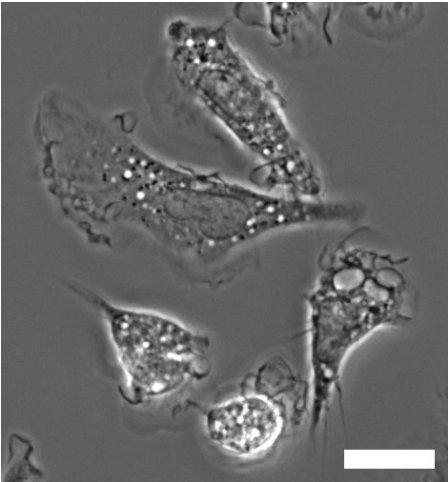

C

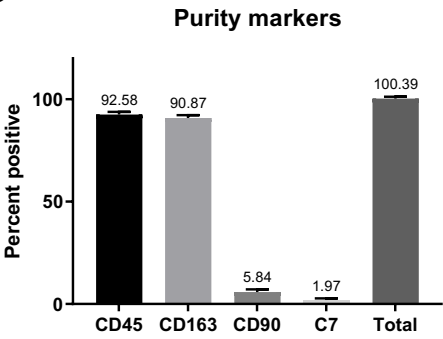

D

| Markers      | % positive  |
|--------------|-------------|
| CD45         | 100         |
| CD163 (M2)   | 98±0.83     |
| CD14 (M1/M2) | 94.56±2.19  |
| CD206 (M2)   | 91.54±1.83  |
| CD209 (M2)   | 1.40±0.21   |
| CD80 (M1)    | ND          |
| CD86 (M1/M2) | 75.43±11.92 |

Supplement: FIG S1 [file mbio.01849-21-sf001.pdf]

A

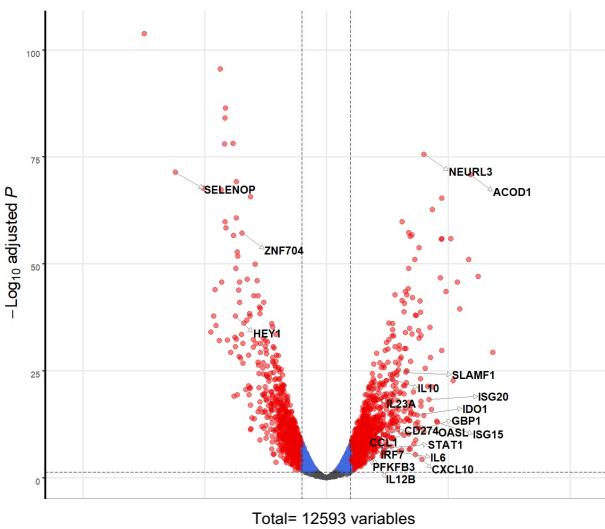

B

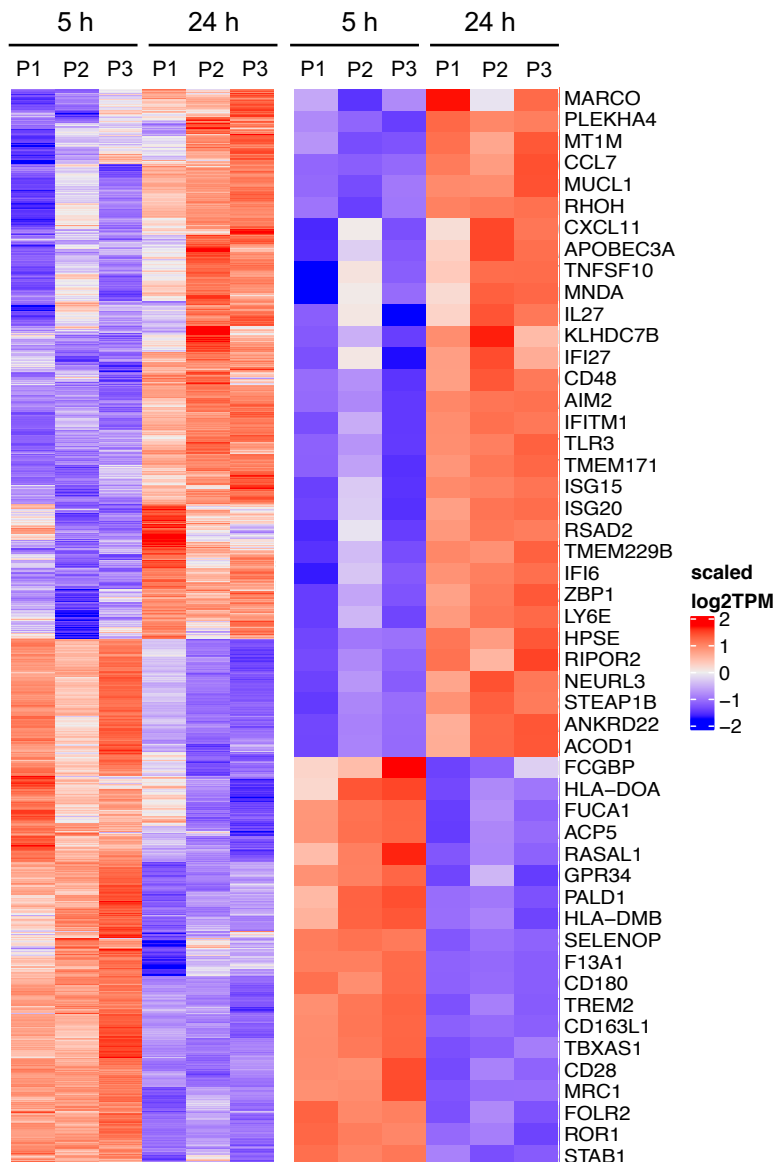

Supplement: FIG S3 [file mbio.01849-21-sf003.pdf]

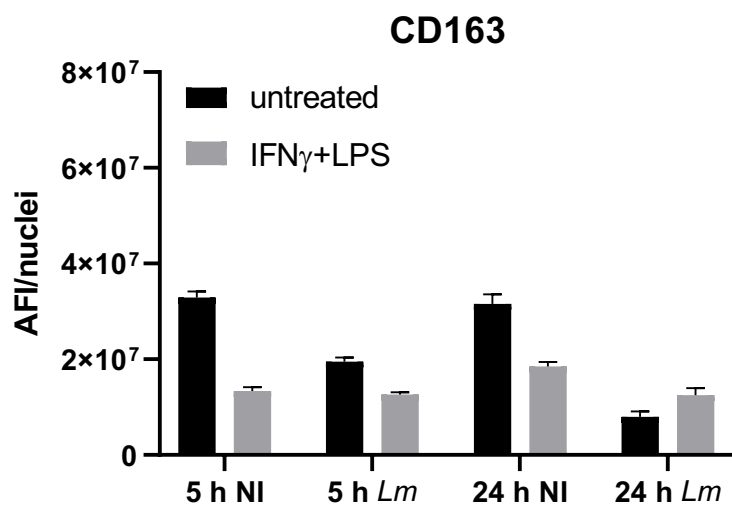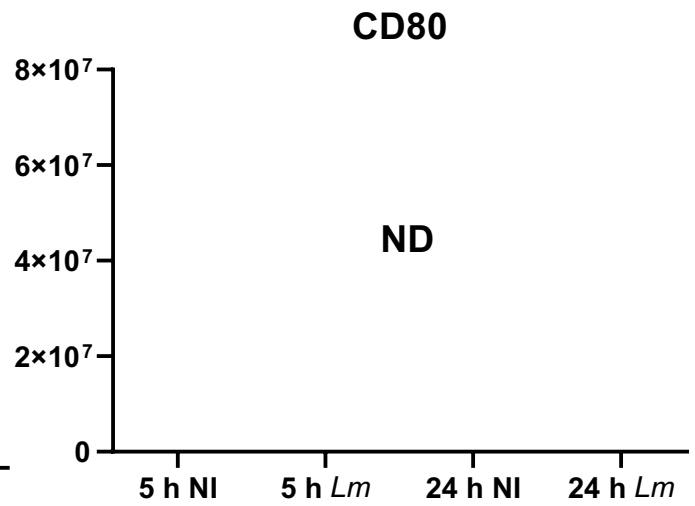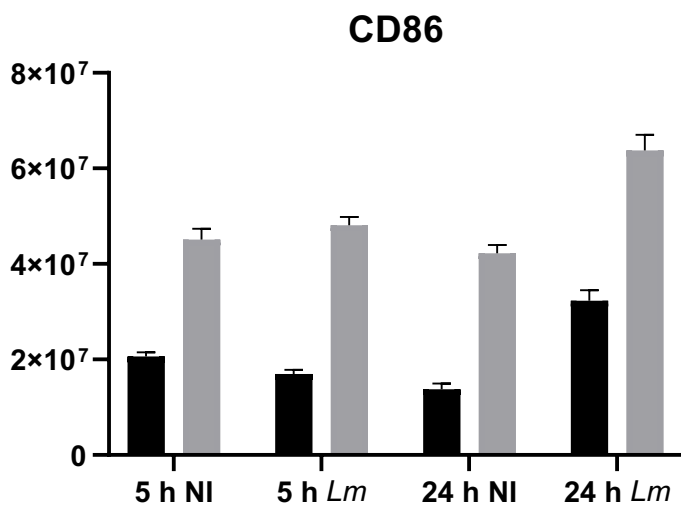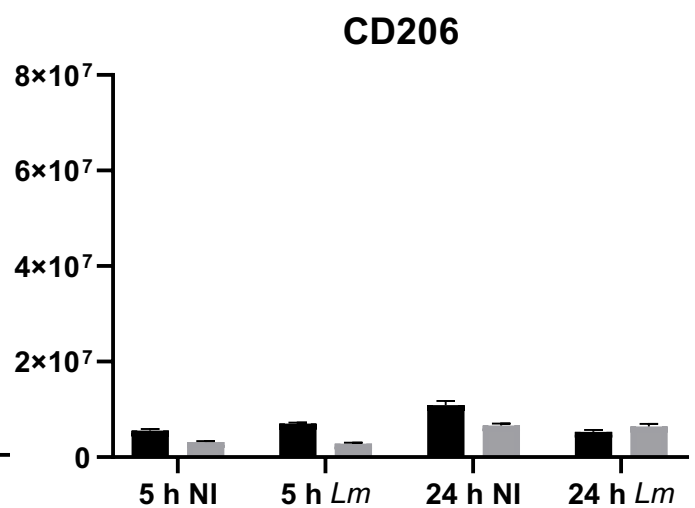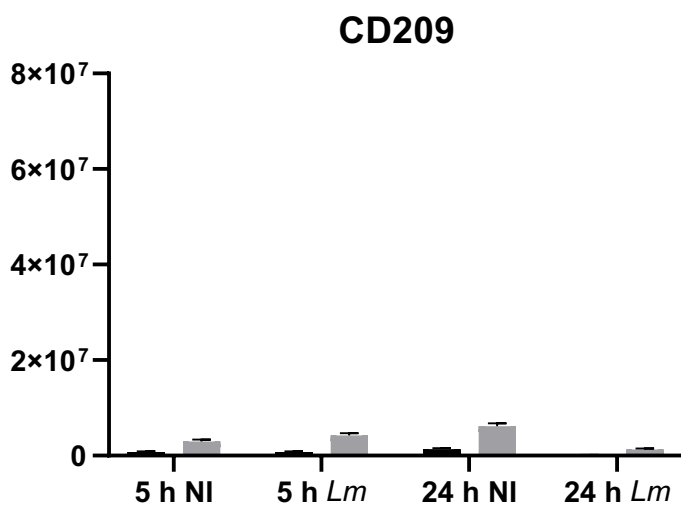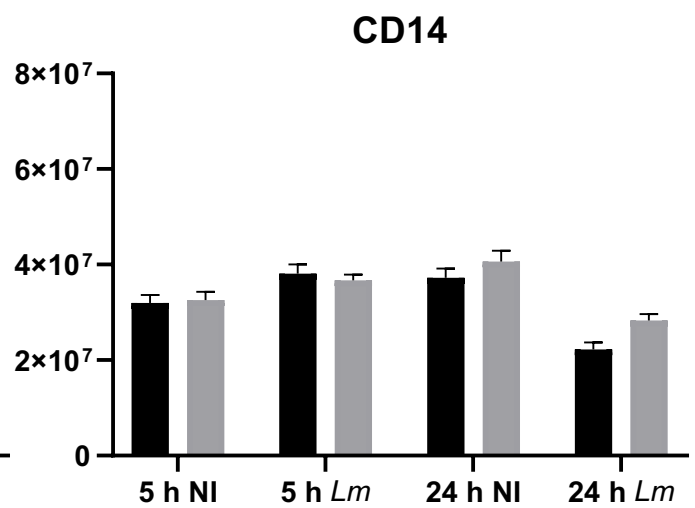

Supplement: FIG S4 [file mbio.01849-21-sf004.pdf]
