# Supplementary material for: Hofbauer Cells Spread Listeria monocytogenes among Placental Cells and Undergo Pro-Inflammatory Reprogramming while Retaining Production of Tolerogenic Factors
Source: mBio. 2021 Aug 17;12(4):e01849-21. doi: 10.1128/mBio.01849-21 (PMC8406333; doi:10.1128/mBio.01849-21)
Supplement: FIG S2 [file mbio.01849-21-sf002.pdf]

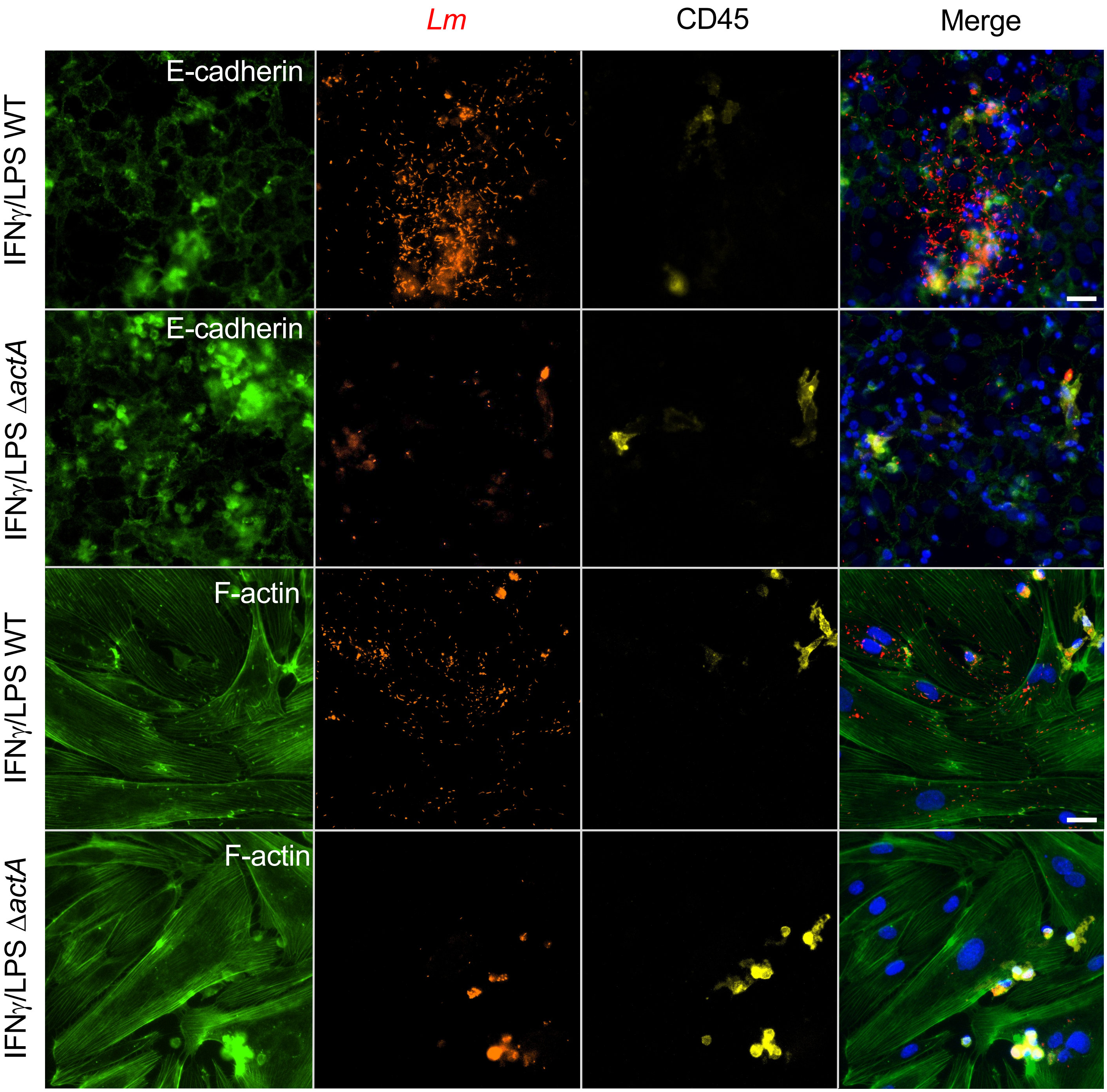

Lm
CD45
Merge
E-cadherin
E-cadherin
F-actin
F-actin
IFNγ/LPS WT
IFNγ/LPS ΔactA
IFNγ/LPS WT
IFNγ/LPS ΔactA
