# Supplementary material for: Hofbauer Cells Spread Listeria monocytogenes among Placental Cells and Undergo Pro-Inflammatory Reprogramming while Retaining Production of Tolerogenic Factors
Source: mBio. 2021 Aug 17;12(4):e01849-21. doi: 10.1128/mBio.01849-21 (PMC8406333; doi:10.1128/mBio.01849-21)
Supplement: TABLE S1 [file mbio.01849-21-st001.docx]

**Table S1.** **Infection by L. monocytogenes upregulates the expression of chemokine-coding genes**. HBCs were stimulated, or not, with IFN-γ/LPS for 24 h prior to Lm infection. Control untreated HBCs (UT), HBCs treated with IFN-γ/LPS, and untreated Lm-infected HBCs were incubated for the indicated time points (h). Data show the fold change expression of chemokine-coding genes (RNA-seq). Upregulated (Log2FC $\geq$ 1) and downregulated (Log2FC $\leq$ -1) genes are highlighted in green and yellow, respectively, and significant FDR values are highlighted in pink. FC (fold change), FDR (false discovery rate), and vs (versus).

| **Chemokines** | **IFNγ/LPS (5 h) vs UT (5 h)** | | ***Lm* (5 h) vs UT (5 h)** | | ***Lm* (24 h) vs *Lm* (5 h)** | |
| --- | --- | --- | --- | --- | --- | --- |
|  | **Log2FC** | **FDR** | **Log2FC** | **FDR** | **Log2FC** | **FDR** |
| CXCL1 | 3.24 | 3.02E-55 | 5.86 | 2.78E-210 | 0.98 | 1.96E-02 |
| CXCL10 | 7.00 | 4.10E-82 | 4.29 | 1.06E-12 | 3.44 | 1.39E-07 |
| CXCL11 | 7.71 | 8.46E-66 | 2.70 | 1.91E-05 | 5.20 | 2.08E-23 |
| CXCL12 | 0.82 | 4.91E-03 | -0.82 | 1.50E-05 | 1.39 | 1.99E-10 |
| CXCL16 | 0.40 | 1.32E-01 | -0.26 | 1.41E-01 | 0.35 | 2.61E-01 |
| CXCL2 | 0.45 | 8.38E-03 | 3.37 | 3.24E-72 | 0.21 | 5.14E-01 |
| CXCL3 | 1.14 | 1.78E-10 | 3.92 | 1.36E-108 | 0.50 | 1.86E-01 |
| CXCL5 | 4.39 | 1.74E-36 | 2.60 | 6.47E-28 | 2.44 | 4.31E-09 |
| CXCL6 | 3.25 | 1.08E-22 | 4.62 | 1.76E-90 | 1.40 | 1.49E-10 |
| CXCL8 | 3.32 | 6.70E-32 | 4.51 | 2.24E-155 | 0.84 | 1.70E-01 |
| CXCL9 | 8.62 | 2.60E-138 | 0.46 | 1.90E-03 | 1.24 | 3.72E-03 |
| CCL1 | 3.75 | 3.93E-13 | 3.23 | 3.50E-18 | 2.12 | 4.05E-11 |
| CCL13 | -2.28 | 7.08E-12 | 0.94 | 6.01E-09 | -1.49 | 3.27E-11 |
| CCL2 | 3.48 | 1.25E-24 | 3.72 | 1.27E-73 | 1.55 | 4.29E-04 |
| CCL20 | 0.70 | 1.14E-01 | 6.16 | 5.93E-278 | 0.26 | 4.09E-01 |
| CCL22 | 1.62 | 1.63E-06 | 1.45 | 1.33E-16 | 0.78 | 8.35E-03 |
| CCL24 | -2.48 | 2.42E-06 | 2.69 | 5.91E-39 | -3.11 | 8.42E-22 |
| CCL3 | 2.59 | 3.91E-16 | 5.91 | 7.18E-129 | 1.08 | 1.65E-02 |
| CCL3L1 | 1.68 | 2.96E-07 | 5.74 | 1.47E-184 | 1.29 | 8.60E-04 |
| CCL4 | 2.37 | 5.52E-21 | 5.86 | 2.28E-149 | 0.28 | 5.51E-01 |
| CCL4L2 | 1.18 | 1.19E-04 | 5.85 | 1.62E-128 | 0.54 | 1.86E-01 |
| CCL5 | 6.23 | 2.97E-69 | 4.16 | 9.39E-121 | 3.00 | 8.16E-31 |
| CCL7 | 6.00 | 8.26E-49 | 3.38 | 1.24E-54 | 5.10 | 1.38E-56 |
| CCL8 | 5.68 | 1.15E-33 | 4.76 | 3.32E-53 | 3.66 | 1.44E-09 |
