# Supplementary material for: Hofbauer Cells Spread Listeria monocytogenes among Placental Cells and Undergo Pro-Inflammatory Reprogramming while Retaining Production of Tolerogenic Factors
Source: mBio. 2021 Aug 17;12(4):e01849-21. doi: 10.1128/mBio.01849-21 (PMC8406333; doi:10.1128/mBio.01849-21)
Supplement: TABLE S2 [file mbio.01849-21-st002.docx]

**Table S2.** **Complete list of the Gene Ontology (GO) terms for the biological processes (BP) based on GOTERM_BP_DIRECT analysis using DAVID 6.8**. The EASE score was set to 0.05 and the processes with an FDR $<$ 0.05 were selected. The terms in bold were used in Fig 9.

| **GO term – induced – *Lm* 5 h versus uninfected 5 h** | **FDR** | **Count** |
| --- | --- | --- |
| **GO:0007165~signal transduction** | 1.88E-07 | 103 |
| **GO:0007267~cell-cell signaling** | 2.17E-06 | 36 |
| **GO:0007166~cell surface receptor signaling pathway** | 3.22E-03 | 30 |
| **GO:0043547~positive regulation of GTPase activity** | 7.69E-03 | 48 |
| **GO:0070374~positive regulation of ERK1 and ERK2 cascade** | 2.22E-06 | 29 |
| **GO:0000165~MAPK cascade** | 1.66E-03 | 30 |
| **GO:0051897~positive regulation of protein kinase B signaling** | 3.97E-05 | 18 |
| GO:0043491~protein kinase B signaling | 3.01E-02 | 8 |
| **GO:0007259~JAK-STAT cascade** | 5.54E-03 | 9 |
| **GO:0035666~TRIF-dependent toll-like receptor signaling pathway** | 2.36E-03 | 9 |
| GO:0046627~negative regulation of insulin receptor signaling pathway | 4.52E-04 | 10 |
| **GO:0051092~positive regulation of NF-kappaB transcription factor activity** | 1.39E-07 | 27 |
| **GO:0043123~positive regulation of I-kappaB kinase/NF-kappaB signaling** | 7.03E-05 | 25 |
| GO:0007249~I-kappaB kinase/NF-kappaB signaling | 3.46E-04 | 14 |
| GO:0042346~positive regulation of NF-kappaB import into nucleus | 2.39E-03 | 8 |
| GO:0009612~response to mechanical stimulus | 1.41E-03 | 13 |
| GO:0071407~cellular response to organic cyclic compound | 5.34E-03 | 12 |
| GO:0070301~cellular response to hydrogen peroxide | 1.54E-02 | 11 |
| GO:0051928~positive regulation of calcium ion transport | 4.08E-02 | 7 |
| GO:1903874~ferrous iron transmembrane transport | 3.59E-02 | 4 |
| GO:1903898~negative regulation of PERK-mediated unfolded protein response | 3.59E-02 | 4 |
| GO:0031668~cellular response to extracellular stimulus | 4.24E-02 | 6 |
| GO:0042493~response to drug | 4.72E-02 | 28 |
| **GO:0001934~positive regulation of protein phosphorylation** | 3.71E-02 | 16 |
| GO:0018108~peptidyl-tyrosine phosphorylation | 3.59E-02 | 18 |
| GO:0045860~positive regulation of protein kinase activity | 4.97E-02 | 9 |
| **GO:0050731~positive regulation of peptidyl-tyrosine phosphorylation** | 2.15E-03 | 15 |
| **GO:0033138~positive regulation of peptidyl-serine phosphorylation** | 1.91E-02 | 12 |
| **GO:0032020~ISG15-protein conjugation** | 4.26E-03 | 5 |
| **GO:0000209~protein polyubiquitination** | 6.25E-04 | 25 |
| GO:0051091~positive regulation of sequence-specific DNA binding transcription factor activity | 1.93E-02 | 15 |
| **GO:0006366~transcription from RNA polymerase II promoter** | 1.95E-02 | 43 |
| **GO:0045944~positive regulation of transcription from RNA polymerase II promoter** | 1.25E-07 | 92 |
| GO:0010628~positive regulation of gene expression | 1.66E-03 | 30 |
| **GO:0032755~positive regulation of interleukin-6 production** | 8.81E-05 | 13 |
| **GO:0032760~positive regulation of tumor necrosis factor production** | 3.67E-03 | 11 |
| **GO:0032729~positive regulation of interferon-gamma production** | 3.19E-03 | 11 |
| GO:0032728~positive regulation of interferon-beta production | 4.72E-02 | 7 |
| **GO:0032481~positive regulation of type I interferon production** | 6.49E-03 | 11 |
| **GO:0050718~positive regulation of interleukin-1 beta secretion** | 1.91E-02 | 7 |
| GO:0050715~positive regulation of cytokine secretion | 6.49E-03 | 8 |
| **GO:0032735~positive regulation of interleukin-12 production** | 6.49E-03 | 8 |
| GO:0045080~positive regulation of chemokine biosynthetic process | 3.22E-03 | 6 |
| **GO:0032722~positive regulation of chemokine production** | 5.33E-03 | 7 |
| GO:0032725~positive regulation of granulocyte macrophage colony-stimulating factor production | 3.21E-02 | 5 |
| GO:0034340~response to type I interferon | 8.06E-03 | 5 |
| GO:0035455~response to interferon-alpha | 3.21E-02 | 5 |
| GO:0035456~response to interferon-beta | 2.11E-02 | 5 |
| **GO:0060337~type I interferon signaling pathway** | 1.73E-16 | 28 |
| GO:0034341~response to interferon-gamma | 8.03E-04 | 9 |
| **GO:0071346~cellular response to interferon-gamma** | 1.93E-08 | 19 |
| GO:0033209~tumor necrosis factor-mediated signaling pathway | 2.01E-02 | 16 |
| **GO:0060333~interferon-gamma-mediated signaling pathway** | 3.84E-11 | 24 |
| **GO:0071356~cellular response to tumor necrosis factor** | 1.40E-08 | 26 |
| **GO:0071347~cellular response to interleukin-1** | 3.84E-11 | 24 |
| GO:0019221~cytokine-mediated signaling pathway | 9.81E-05 | 22 |
| **GO:0070098~chemokine-mediated signaling pathway** | 3.31E-10 | 23 |
| GO:0060326~cell chemotaxis | 1.68E-04 | 15 |
| **GO:0006935~chemotaxis** | 3.26E-05 | 22 |
| GO:0030593~neutrophil chemotaxis | 1.95E-07 | 19 |
| GO:0002548~monocyte chemotaxis | 6.78E-07 | 15 |
| **GO:0034097~response to cytokine** | 1.13E-05 | 15 |
| GO:0048247~lymphocyte chemotaxis | 2.36E-03 | 9 |
| **GO:0030335~positive regulation of cell migration** | 1.66E-03 | 24 |
| GO:0090023~positive regulation of neutrophil chemotaxis | 1.91E-02 | 7 |
| GO:0048245~eosinophil chemotaxis | 2.11E-02 | 5 |
| GO:0002690~positive regulation of leukocyte chemotaxis | 4.24E-02 | 6 |
| GO:0014911~positive regulation of smooth muscle cell migration | 1.23E-02 | 7 |
| GO:0048661~positive regulation of smooth muscle cell proliferation | 1.13E-05 | 16 |
| GO:0008285~negative regulation of cell proliferation | 4.41E-07 | 49 |
| GO:0030890~positive regulation of B cell proliferation | 1.91E-02 | 9 |
| GO:0044344~cellular response to fibroblast growth factor stimulus | 1.91E-02 | 8 |
| GO:0042104~positive regulation of activated T cell proliferation | 4.72E-02 | 7 |
| GO:0008284~positive regulation of cell proliferation | 3.41E-02 | 39 |
| GO:0008283~cell proliferation | 4.73E-02 | 32 |
| GO:0042127~regulation of cell proliferation | 2.44E-05 | 28 |
| **GO:0042102~positive regulation of T cell proliferation** | 6.06E-03 | 12 |
| GO:0045444~fat cell differentiation | 2.51E-02 | 12 |
| GO:0001541~ovarian follicle development | 2.78E-02 | 9 |
| GO:0008637~apoptotic mitochondrial changes | 1.19E-04 | 9 |
| **GO:0006915~apoptotic process** | 7.32E-08 | 64 |
| **GO:0043066~negative regulation of apoptotic process** | 2.71E-04 | 46 |
| **GO:0043065~positive regulation of apoptotic process** | 1.41E-03 | 33 |
| GO:2001240~negative regulation of extrinsic apoptotic signaling pathway in absence of ligand | 3.01E-03 | 10 |
| GO:0001836~release of cytochrome c from mitochondria | 4.15E-03 | 8 |
| GO:0070059~intrinsic apoptotic signaling pathway in response to endoplasmic reticulum stress | 6.49E-03 | 9 |
| GO:0006919~activation of cysteine-type endopeptidase activity involved in apoptotic process | 7.40E-03 | 14 |
| **GO:0042981~regulation of apoptotic process** | 1.08E-02 | 24 |
| GO:0008630~intrinsic apoptotic signaling pathway in response to DNA damage | 1.54E-02 | 10 |
| GO:0070234~positive regulation of T cell apoptotic process | 2.11E-02 | 5 |
| GO:2001243~negative regulation of intrinsic apoptotic signaling pathway | 1.91E-02 | 7 |
| GO:0097192~extrinsic apoptotic signaling pathway in absence of ligand | 3.48E-02 | 8 |
| GO:0050728~negative regulation of inflammatory response | 1.61E-02 | 13 |
| **GO:0006954~inflammatory response** | 3.30E-29 | 84 |
| GO:0050727~regulation of inflammatory response | 3.56E-06 | 17 |
| **GO:0045087~innate immune response** | 3.11E-07 | 52 |
| **GO:0006955~immune response** | 2.36E-16 | 69 |
| **GO:0050729~positive regulation of inflammatory response** | 2.85E-08 | 21 |
| GO:0045824~negative regulation of innate immune response | 4.39E-02 | 5 |
| GO:0006959~humoral immune response | 4.69E-02 | 10 |
| GO:0006952~defense response | 1.13E-03 | 14 |
| GO:0002230~positive regulation of defense response to virus by host | 1.91E-02 | 7 |
| **GO:0051607~defense response to virus** | 7.83E-22 | 49 |
| **GO:0009615~response to virus** | 5.69E-17 | 36 |
| GO:0045071~negative regulation of viral genome replication | 4.19E-09 | 17 |

| **GO term – induced – *Lm* 5 h versus *Lm* 24 h** | **FDR** | **Count** |
| --- | --- | --- |
| GO:0007165~signal transduction | 4.17E-03 | 85 |
| GO:0007267~cell-cell signaling | 5.49E-03 | 28 |
| GO:0043547~positive regulation of GTPase activity | 5.20E-05 | 23 |
| GO:0070374~positive regulation of ERK1 and ERK2 cascade | 8.16E-04 | 24 |
| GO:0007259~JAK-STAT cascade | 2.56E-04 | 24 |
| GO:0051092~positive regulation of NF-kappaB transcription factor activity | 1.77E-04 | 9 |
| GO:0043123~positive regulation of I-kappaB kinase/NF-kappaB signaling | 1.15E-03 | 10 |
| GO:0071407~cellular response to organic cyclic compound | 4.73E-03 | 10 |
| GO:0051384~response to glucocorticoid | 2.36E-02 | 11 |
| GO:0071294~cellular response to zinc ion | 3.96E-02 | 45 |
| GO:0071276~cellular response to cadmium ion | 4.82E-02 | 11 |
| GO:0042517~positive regulation of tyrosine phosphorylation of Stat3 protein | 4.82E-02 | 6 |
| GO:0051091~positive regulation of sequence-specific DNA binding transcription factor activity | 8.61E-03 | 16 |
| GO:0032760~positive regulation of tumor necrosis factor production | 1.86E-04 | 13 |
| GO:0032480~negative regulation of type I interferon production | 4.83E-03 | 9 |
| GO:0032731~positive regulation of interleukin-1 beta production | 1.84E-02 | 5 |
| GO:0032693~negative regulation of interleukin-10 production | 2.07E-25 | 35 |
| GO:0035455~response to interferon-alpha | 1.35E-04 | 10 |
| GO:0035456~response to interferon-beta | 2.56E-04 | 14 |
| GO:0034612~response to tumor necrosis factor | 3.90E-02 | 11 |
| GO:0035458~cellular response to interferon-beta | 4.97E-03 | 7 |
| GO:0034341~response to interferon-gamma | 1.35E-04 | 7 |
| GO:0071346~cellular response to interferon-gamma | 8.30E-03 | 13 |
| GO:0071347~cellular response to interleukin-1 | 5.29E-04 | 15 |
| GO:0060337~type I interferon signaling pathway | 2.53E-14 | 27 |
| GO:0060333~interferon-gamma-mediated signaling pathway | 1.61E-05 | 8 |
| GO:0019221~cytokine-mediated signaling pathway | 2.56E-02 | 17 |
| GO:0070098~chemokine-mediated signaling pathway | 9.78E-03 | 6 |
| GO:0060326~cell chemotaxis | 1.31E-02 | 12 |
| GO:0006935~chemotaxis | 4.85E-04 | 20 |
| GO:0002548~monocyte chemotaxis | 8.64E-03 | 10 |
| GO:0030335~positive regulation of cell migration | 1.04E-02 | 22 |
| GO:0045926~negative regulation of growth | 1.80E-05 | 10 |
| GO:0008284~positive regulation of cell proliferation | 7.00E-04 | 16 |
| GO:0008285~negative regulation of cell proliferation | 6.99E-03 | 42 |
| GO:0042102~positive regulation of T cell proliferation | 4.16E-04 | 14 |
| GO:0043065~positive regulation of apoptotic process | 1.04E-02 | 5 |
| GO:0006919~activation of cysteine-type endopeptidase activity involved in apoptotic process | 4.82E-02 | 7 |
| GO:0006954~inflammatory response | 5.38E-13 | 59 |
| GO:0050776~regulation of immune response | 7.68E-03 | 22 |
| GO:0045087~innate immune response | 1.98E-17 | 71 |
| GO:0006955~immune response | 5.06E-11 | 59 |
| GO:0050729~positive regulation of inflammatory response | 7.00E-04 | 15 |
| GO:0006952~defense response | 2.89E-04 | 15 |
| GO:0002230~positive regulation of defense response to virus by host | 3.42E-07 | 12 |
| GO:0051607~defense response to virus | 7.57E-29 | 56 |
| GO:0009615~response to virus | 1.03E-13 | 32 |
| GO:0045071~negative regulation of viral genome replication | 2.35E-22 | 27 |
| **GO:0019885~antigen processing and presentation of endogenous peptide via MHC class I** | 8.64E-03 | 37 |
| **GO:0002479~antigen processing and presentation of exogenous peptide via MHC class I, TAP-dependent** | 1.27E-02 | 30 |
| GO:0002474~antigen processing and presentation of peptide via MHC class I | 1.29E-04 | 11 |
| GO:0002480~antigen processing and presentation of exogenous peptide via MHC class I, TAP-independent | 1.35E-04 | 7 |
| GO:0002250~adaptive immune response | 2.55E-05 | 25 |
| GO:0001916~positive regulation of T cell mediated cytotoxicity | 1.36E-03 | 7 |

| **GO term – repressed – *Lm* 5 h versus *Lm* 24 h** | **FDR** | **Count** |
| --- | --- | --- |
| DNA replication-dependent nucleosome assembly (GO:0006335) | 6.03E-08 | 38 |
| rRNA processing (GO:0006364) | 6.03E-08 | 12 |
| telomere capping (GO:0016233) | 9.88E-05 | 20 |
| rRNA transcription (GO:0009303) | 1.72E-04 | 60 |
| positive regulation of gene expression, epigenetic (GO:0045815) | 2.75E-04 | 27 |
| DNA replication-independent nucleosome assembly (GO:0006336) | 7.81E-04 | 15 |
| CENP-A containing nucleosome assembly (GO:0034080) | 3.92E-02 | 8 |
| T cell costimulation (GO:0031295) | 4.25E-02 | 16 |
| platelet degranulation (GO:0002576) | 4.25E-02 | 7 |
| negative regulation of megakaryocyte differentiation (GO:0045653) | 4.25E-02 | 10 |
| carbohydrate metabolic process (GO:0005975) | 4.25E-02 | 6 |
| oxidation-reduction process (GO:0055114) | 4.43E-02 | 8 |
| protein heterotetramerization (GO:0051290) | 4.43E-02 | 10 |
| antigen processing and presentation of exogenous peptide antigen via MHC class II (GO:0019886) | 4.25E-02 | 14 |
| antigen processing and presentation of peptide or polysaccharide antigen via MHC class II (GO:0002504) | 4.25E-02 | 9 |
