# Supplementary material for: Hofbauer Cells Spread Listeria monocytogenes among Placental Cells and Undergo Pro-Inflammatory Reprogramming while Retaining Production of Tolerogenic Factors
Source: mBio. 2021 Aug 17;12(4):e01849-21. doi: 10.1128/mBio.01849-21 (PMC8406333; doi:10.1128/mBio.01849-21)
Supplement: TABLE S3 [file mbio.01849-21-st003.docx]

**Table S3.** **Expression of M1- and M2-signature genes by HBCs**. HBCs were stimulated, or not, with IFN-γ/LPS for 24 h prior to Lm infection. Control untreated HBCs (UT), HBCs treated with IFN-γ/LPS, and untreated Lm-infected HBCs were incubated for the indicated time points (h). Data show the fold change expression of M1- and M2-signature genes (RNA-seq). Upregulated (Log2FC $\geq$ 1) and downregulated (Log2FC $\leq$ -1) genes are highlighted in green and yellow, respectively, and significant FDR values are highlighted in pink. FC (fold change), FDR (false discovery rate), vs (versus), and ND (not detected).

| **M1** | **IFNγ/LPS (5) vs UT (5)** | | ***Lm* (5) vs UT (5)** | | ***Lm* (24) vs *Lm* (5)** | |
| --- | --- | --- | --- | --- | --- | --- |
|  | **Log2FC** | **FDR** | **Log2FC** | **Log2FC** | **FDR** | **Log2FC** |
| NFKB1 | -0.49 | 1.67E-02 | 3.19 | 1.42E-89 | -1.17 | 3.74E-04 |
| NFKB2 | 1.13 | 2.97E-09 | 3.24 | 2.96E-69 | -0.11 | 7.98E-01 |
| NFKBIA | 1.57 | 3.12E-13 | 2.67 | 1.47E-61 | 0.23 | 5.76E-01 |
| STAT1 | 3.71 | 6.49E-54 | 1.53 | 1.82E-08 | 2.21 | 1.43E-07 |
| IRF1 | 4.20 | 2.12E-62 | 2.05 | 9.64E-25 | 0.34 | 1.98E-01 |
| IRF5 | 0.79 | 3.19E-05 | -0.10 | 5.67E-01 | 0.68 | 3.48E-04 |
| HIF1A | -1.78 | 4.77E-12 | -0.36 | 1.86E-02 | -0.31 | 4.12E-01 |
| KLF6 | 1.13 | 2.47E-06 | 1.76 | 5.90E-19 | 0.85 | 1.44E-02 |
| TNF | 2.36 | 2.00E-47 | 7.25 | 3.14E-228 | -0.24 | 5.85E-01 |
| IL1B | 2.92 | 2.73E-10 | 6.69 | 9.06E-195 | 0.65 | 2.53E-01 |
| IL6 | 1.77 | 7.91E-06 | 5.62 | 7.58E-143 | 2.29 | 2.43E-07 |
| IL12B | 0.15 | 8.46E-01 | 7.10 | 5.26E-96 | 1.91 | 1.19E-05 |
| IL23A | 0.15 | 7.57E-01 | 4.62 | 6.91E-166 | 1.74 | 4.90E-22 |
| CXCL9 | 8.62 | 2.60E-138 | 0.46 | 1.90E-03 | 1.24 | 3.72E-03 |
| CXCL10 | 7.00 | 4.10E-82 | 4.29 | 1.06E-12 | 3.44 | 1.39E-07 |
| CXCL11 | 7.71 | 8.46E-66 | 2.70 | 1.91E-05 | 5.20 | 2.08E-23 |
| CYBB | 0.59 | 7.59E-02 | 0.46 | 2.60E-03 | -1.11 | 1.96E-03 |
| CYBA | 0.56 | 1.62E-02 | 0.14 | 4.11E-01 | -0.11 | 7.09E-01 |
| FTL | -0.75 | 1.15E-02 | -0.28 | 6.93E-02 | -0.68 | 1.63E-01 |
| FTH1 | 0.62 | 6.48E-02 | 0.40 | 8.61E-03 | -0.05 | 9.32E-01 |
| SLC11A2 | 0.14 | 5.85E-01 | 1.29 | 6.70E-20 | 0.47 | 5.38E-02 |
| SLC11A1 | 0.55 | 2.64E-02 | -0.58 | 5.13E-05 | 0.81 | 2.07E-03 |
| CD80 | 3.67 | 4.27E-63 | 4.23 | 4.13E-115 | 2.01 | 1.62E-18 |
| CD86 | 0.17 | 4.83E-01 | -0.82 | 5.54E-09 | 0.13 | 6.62E-01 |
| TLR2 | 1.97 | 5.68E-19 | 2.01 | 9.76E-42 | 0.09 | 8.17E-01 |
| TLR4 | -0.32 | 2.33E-01 | -0.83 | 2.86E-07 | -0.80 | 2.21E-04 |
| CSF2 | 0.57 | 2.35E-01 | 5.03 | 4.57E-54 | 1.57 | 2.14E-15 |
| ITGAX | -0.42 | 7.94E-02 | 1.07 | 2.15E-13 | -1.32 | 3.45E-08 |
| CD40 | 3.29 | 5.89E-49 | 3.77 | 1.53E-47 | 0.75 | 3.02E-02 |
| STAT2 | 1.81 | 1.63E-20 | 1.27 | 4.84E-08 | 2.04 | 5.10E-09 |
| STAT5A | 0.44 | 4.03E-02 | 0.45 | 2.64E-03 | -0.48 | 6.96E-02 |
| STAT5B | -0.41 | 1.90E-02 | -0.21 | 1.12E-01 | -0.47 | 1.08E-02 |
| IRF3 | 0.55 | 7.25E-04 | -0.57 | 1.32E-05 | 0.57 | 3.45E-03 |
| JUN | -0.04 | 8.91E-01 | 1.37 | 1.20E-20 | 0.06 | 8.59E-01 |
| MAPK14 | 0.16 | 4.86E-01 | -0.54 | 2.17E-05 | 0.10 | 6.78E-01 |
| CCR7 | 3.17 | 2.12E-08 | 5.97 | 8.80E-61 | 1.53 | 2.08E-04 |
| HLA-DMA | -0.18 | 6.11E-01 | -0.52 | 7.06E-05 | -3.61 | 1.40E-44 |
| HLA-DMB | -0.24 | 5.57E-01 | -1.26 | 1.63E-21 | -4.53 | 2.76E-36 |
| HLA-DOA | -0.21 | 7.24E-01 | -0.33 | 2.35E-02 | -5.05 | 1.95E-68 |
| HLA-DPA1 | 0.87 | 1.33E-02 | 0.09 | 6.02E-01 | -1.79 | 5.16E-18 |
| HLA-DPB1 | -0.23 | 5.95E-01 | -0.03 | 8.30E-01 | -2.21 | 5.71E-36 |
| HLA-DQA1 | 2.08 | 7.19E-04 | 0.70 | 5.75E-07 | -2.16 | 8.11E-12 |
| HLA-DQA2 | 2.07 | 8.02E-04 | 0.57 | 7.87E-03 | -1.61 | 5.88E-07 |
| HLA-DQB1 | 0.47 | 1.91E-01 | 0.32 | 2.82E-02 | -3.20 | 3.50E-22 |
| HLA-DQB2 | 0.35 | 5.90E-01 | 0.09 | 7.54E-01 | -3.18 | 4.05E-20 |
| HLA-DRA | 0.82 | 1.17E-02 | 0.38 | 1.28E-02 | -2.63 | 4.66E-16 |
| HLA-DRB1 | 0.65 | 4.31E-02 | 0.22 | 1.43E-01 | -2.26 | 1.44E-14 |
| HLA-DRB5 | 0.52 | 5.70E-02 | 0.16 | 2.82E-01 | -2.00 | 4.27E-18 |
| HLA-DRB6 | 0.64 | 1.08E-01 | 0.25 | 2.17E-01 | -3.16 | 1.72E-21 |
| NOTCH1 | 0.21 | 4.58E-01 | -0.73 | 6.81E-07 | -0.09 | 7.20E-01 |
| NOTCH2 | 0.11 | 7.16E-01 | 0.70 | 3.16E-06 | -0.53 | 1.01E-01 |
| NOTCH2NL | -0.57 | 1.67E-03 | 0.40 | 5.17E-03 | -0.61 | 2.69E-03 |
| NOTCH3 | -1.55 | 2.90E-12 | 1.14 | 2.14E-13 | -3.15 | 4.27E-39 |
| EPSTI1 | 3.14 | 2.54E-67 | 2.35 | 1.80E-19 | 2.19 | 3.95E-12 |
| BCL2A1 | 3.61 | 1.64E-54 | 5.75 | 4.58E-147 | 0.63 | 1.76E-02 |
| MYD88 | 1.26 | 1.69E-13 | 1.41 | 4.29E-10 | 1.01 | 6.02E-04 |
| TRAF6 | 0.16 | 4.48E-01 | 0.31 | 2.65E-02 | 0.21 | 3.73E-01 |
| IRAK1 | 0.05 | 8.33E-01 | 0.01 | 9.44E-01 | 0.16 | 5.13E-01 |
| IRAK2 | 1.86 | 1.50E-15 | 4.07 | 2.88E-152 | 0.14 | 6.46E-01 |
| RELA | 1.01 | 5.82E-09 | 1.50 | 1.16E-23 | 0.59 | 8.40E-03 |
| IL1A | 1.69 | 1.91E-07 | 6.96 | 1.10E-154 | -0.74 | 4.51E-02 |
| CXCL1 | 3.24 | 3.02E-55 | 5.86 | 2.78E-210 | 0.98 | 1.96E-02 |
| CCL20 | 0.70 | 1.14E-01 | 6.16 | 5.93E-278 | 0.26 | 4.09E-01 |
| CCL3 | 2.59 | 3.91E-16 | 5.91 | 7.18E-129 | 1.08 | 1.65E-02 |
| CCL4 | 2.37 | 5.52E-21 | 5.86 | 2.28E-149 | 0.28 | 5.51E-01 |
| CCL5 | 6.23 | 2.97E-69 | 4.16 | 9.39E-121 | 3.00 | 8.16E-31 |
| CXCL8 | 3.32 | 6.70E-32 | 4.51 | 2.24E-155 | 0.84 | 1.70E-01 |
| GBP5 | 8.45 | 1.05E-154 | 1.62 | 4.85E-17 | 3.02 | 2.02E-28 |
| GBP1 | 6.72 | 1.86E-96 | 2.98 | 2.37E-13 | 2.65 | 7.45E-10 |
| CIITA | 0.97 | 1.96E-03 | -3.97 | 9.94E-108 | -1.18 | 2.25E-07 |
| HLA-DOB | ND | ND | ND | ND | ND | ND |
| NOS1 | ND | ND | ND | ND | ND | ND |
| NOS2 | ND | ND | ND | ND | ND | ND |
| NOS3 | ND | ND | ND | ND | ND | ND |
| TANK | 1.28 | 5.29E-14 | 1.79 | 2.56E-43 | 0.39 | 1.19E-01 |
| **M2** |  |  |  |  |  |  |
| STAT3 | 0.81 | 1.24E-04 | 0.21 | 1.84E-01 | 1.01 | 1.42E-04 |
| STAT6 | 0.73 | 7.19E-05 | 0.43 | 2.71E-03 | 0.91 | 1.05E-05 |
| IRF4 | 1.57 | 2.84E-07 | 0.98 | 3.92E-05 | 2.09 | 3.88E-11 |
| KLF4 | -0.24 | 3.37E-01 | -0.01 | 9.62E-01 | 2.05 | 9.51E-30 |
| PPARG | -1.05 | 1.15E-07 | 0.26 | 7.96E-02 | -2.09 | 6.40E-19 |
| MAF | -3.35 | 7.98E-27 | -3.05 | 1.50E-72 | -0.78 | 3.30E-04 |
| MYC | -1.44 | 4.49E-09 | 1.98 | 1.18E-13 | -1.33 | 8.82E-10 |
| IL10 | -3.43 | 3.04E-11 | -0.13 | 5.61E-01 | 2.67 | 7.39E-24 |
| TGFB1 | 0.29 | 1.89E-01 | 0.09 | 5.96E-01 | -0.05 | 8.77E-01 |
| TGFB2 | -1.35 | 7.49E-07 | -0.26 | 6.81E-02 | -0.42 | 9.31E-02 |
| TGFB3 | -1.43 | 5.02E-05 | 0.08 | 7.09E-01 | -0.68 | 4.79E-02 |
| TGFBR1 | -2.20 | 1.08E-26 | -0.70 | 3.07E-05 | -1.23 | 4.74E-07 |
| TGFBR2 | -1.28 | 8.91E-10 | -1.75 | 2.19E-25 | -1.27 | 4.29E-11 |
| TGFBR3 | -1.56 | 3.30E-14 | -0.12 | 4.46E-01 | -0.87 | 1.39E-05 |
| CCL17 | ND | ND | ND | ND | ND | ND |
| CCL18 | ND | ND | ND | ND | ND | ND |
| CCL22 | 1.62 | 1.63E-06 | 1.45 | 1.33E-16 | 0.78 | 8.35E-03 |
| ARG1 | ND | ND | ND | ND | ND | ND |
| ARG2 | 0.13 | 6.01E-01 | -0.56 | 3.03E-04 | 0.92 | 2.79E-06 |
| ODC1 | 0.01 | 9.62E-01 | 0.19 | 1.84E-01 | 0.02 | 9.34E-01 |
| SMO | -0.70 | 4.91E-03 | -0.78 | 1.20E-06 | -1.37 | 1.58E-05 |
| HMOX1 | -0.29 | 3.87E-01 | -1.07 | 5.45E-13 | -1.83 | 1.45E-10 |
| HMOX2 | -0.27 | 2.11E-01 | -0.09 | 5.28E-01 | -0.54 | 1.51E-02 |
| SLC40A1 | -2.67 | 4.15E-33 | -1.07 | 6.58E-17 | -2.57 | 1.11E-41 |
| TFRC | -2.06 | 1.31E-23 | 1.26 | 1.07E-13 | 0.55 | 2.07E-01 |
| MRC1 | -4.97 | 2.32E-47 | -0.74 | 1.55E-06 | -4.31 | 2.03E-46 |
| CD163 | -0.42 | 3.08E-01 | -0.66 | 7.72E-06 | -0.20 | 6.21E-01 |
| CD209 | -0.27 | 3.27E-01 | 0.05 | 7.65E-01 | 0.28 | 2.34E-01 |
| CD301 | ND | ND | ND | ND | ND | ND |
| RETNLB | ND | ND | ND | ND | ND | ND |
| CHI3L1 | 4.33 | 3.25E-33 | 0.90 | 7.17E-13 | 2.72 | 2.63E-35 |
| CHI3L2 | 3.13 | 6.37E-28 | 0.55 | 6.83E-02 | 2.45 | 2.83E-20 |
| FOLR2 | -4.12 | 1.43E-40 | -0.32 | 2.97E-02 | -3.81 | 2.19E-57 |
| VEGFA | -1.82 | 3.29E-13 | 0.68 | 1.41E-05 | -0.39 | 2.52E-01 |
| VEGFB | -1.34 | 2.51E-16 | -0.89 | 2.65E-13 | -1.15 | 1.47E-08 |
| VEGFC | -0.22 | 3.49E-01 | 0.43 | 2.35E-03 | 1.10 | 3.30E-09 |
| SMAD2 | 0.01 | 9.84E-01 | 0.57 | 5.83E-05 | -0.53 | 7.49E-03 |
| SMAD3 | -1.50 | 1.71E-15 | 0.68 | 8.09E-06 | -0.59 | 2.90E-02 |
| SMAD1 | 0.34 | 2.26E-01 | -0.18 | 4.86E-01 | 1.11 | 3.53E-05 |
| SMAD5 | -0.67 | 3.78E-05 | -0.32 | 1.34E-02 | -0.55 | 6.47E-03 |
| CSF1 | 2.91 | 2.75E-41 | 3.15 | 1.15E-44 | -0.60 | 1.29E-02 |
| CSF1R | -1.44 | 2.02E-09 | -0.94 | 3.01E-10 | -0.78 | 1.03E-02 |
| HAVCR2 | -0.03 | 9.13E-01 | -1.36 | 1.92E-19 | 1.00 | 1.57E-07 |
| HLA-G | 1.15 | 1.19E-03 | 0.43 | 1.25E-01 | 1.31 | 2.93E-07 |
| JAK3 | 2.28 | 1.60E-26 | 0.77 | 1.50E-08 | 2.13 | 7.39E-15 |
| KDM6B | 0.17 | 4.13E-01 | 3.11 | 3.95E-87 | -1.33 | 2.90E-08 |
| PIK3C2B | -2.12 | 5.05E-19 | -2.92 | 1.83E-76 | -1.16 | 1.61E-03 |
| PIK3IP1 | -1.92 | 7.79E-33 | -2.66 | 4.65E-67 | -0.36 | 1.70E-01 |
| DAB2 | -1.98 | 2.16E-16 | -1.24 | 3.30E-15 | -0.90 | 5.65E-04 |
| AP2A2 | -1.10 | 7.70E-09 | -1.48 | 6.36E-28 | -0.06 | 8.07E-01 |
| MSR1 | -0.94 | 6.16E-05 | -0.87 | 2.99E-09 | 1.09 | 3.16E-04 |
| AP1B1 | -1.88 | 1.07E-15 | -1.48 | 2.61E-23 | -1.19 | 6.03E-07 |
| ITSN1 | -0.71 | 1.44E-04 | -1.52 | 1.53E-31 | 0.92 | 3.03E-07 |
| MERTK | -3.27 | 6.67E-62 | -3.56 | 3.06E-106 | -1.62 | 2.07E-10 |
| THBS1 | -1.86 | 2.84E-10 | 0.22 | 1.66E-01 | -0.51 | 2.03E-01 |
| EPHB3 | -3.38 | 4.90E-17 | -4.12 | 1.20E-120 | 0.86 | 6.86E-02 |
| RHOB | -0.61 | 4.16E-03 | -0.46 | 6.52E-03 | 0.27 | 1.53E-01 |
| ZFP36L1 | -0.76 | 1.43E-04 | -1.01 | 6.13E-12 | -0.03 | 9.03E-01 |
| SGPL1 | -1.27 | 8.82E-09 | 0.04 | 8.40E-01 | -1.48 | 2.83E-14 |
| CELSR1 | -2.35 | 8.83E-20 | -0.72 | 1.10E-07 | -1.73 | 1.56E-18 |
| HRH1 | 0.16 | 5.25E-01 | -0.08 | 6.57E-01 | 1.89 | 4.98E-25 |
| ITGA9 | -0.84 | 1.56E-05 | -0.68 | 2.15E-06 | -1.22 | 2.72E-08 |
| MAP3K1 | -0.38 | 4.19E-02 | -1.15 | 7.67E-19 | 0.67 | 4.90E-03 |
| PLTP | -2.21 | 6.85E-20 | -0.04 | 8.51E-01 | -1.91 | 2.82E-08 |
| NISCH | -0.66 | 1.30E-03 | -1.48 | 8.13E-26 | 0.43 | 2.55E-02 |
| MGAT4A | -1.66 | 8.15E-13 | -2.00 | 7.19E-46 | 0.21 | 3.32E-01 |
| XYLT1 | -1.41 | 8.15E-17 | -0.14 | 3.27E-01 | 0.21 | 3.49E-01 |
| B3GNT2 | 0.06 | 8.15E-01 | -0.04 | 7.97E-01 | 1.17 | 7.73E-09 |
| MGAT5 | -1.17 | 1.01E-08 | -0.22 | 1.52E-01 | -1.78 | 2.21E-21 |
| ID3 | -1.33 | 3.35E-06 | -1.53 | 2.75E-18 | -0.47 | 8.70E-02 |
| KLF2 | -1.44 | 2.57E-06 | -0.36 | 3.19E-02 | -0.47 | 3.19E-02 |
| IL27RA | 1.25 | 2.60E-12 | 1.57 | 1.36E-29 | -2.59 | 2.58E-33 |
| IGF1 | -4.57 | 5.43E-75 | -2.22 | 2.89E-70 | -1.59 | 9.65E-05 |
| CLEC4A | -0.77 | 7.98E-02 | 0.36 | 1.00E-01 | -1.18 | 1.03E-04 |
| STAB1 | -4.78 | 1.08E-44 | -0.67 | 6.41E-06 | -4.74 | 9.98E-35 |
| F13A1 | -5.83 | 9.69E-53 | -0.73 | 1.74E-06 | -7.48 | 1.45E-104 |
| SPHK1 | 0.74 | 1.39E-04 | 1.18 | 1.49E-14 | -0.02 | 9.46E-01 |
| SCARB1 | -2.05 | 1.12E-29 | -1.51 | 7.07E-22 | -1.48 | 5.35E-09 |
| FCER2 | 4.13 | 4.26E-28 | 0.80 | 2.39E-02 | 1.07 | 4.84E-03 |
| CCL1 | 3.75 | 3.93E-13 | 3.23 | 3.50E-18 | 2.12 | 4.05E-11 |
