# Supplementary material for: Hofbauer Cells Spread Listeria monocytogenes among Placental Cells and Undergo Pro-Inflammatory Reprogramming while Retaining Production of Tolerogenic Factors
Source: mBio. 2021 Aug 17;12(4):e01849-21. doi: 10.1128/mBio.01849-21 (PMC8406333; doi:10.1128/mBio.01849-21)
Supplement: TABLE S4 [file mbio.01849-21-st004.docx]

**Table S4.** **Effect of** ***L. monocytogenes* infection and IFNγ/LPS stimulation on M1 and M2 metabolic genes**. Control untreated HBCs (UT), HBCs treated with IFN-γ/LPS, and untreated *Lm-*infected HBCs (*Lm*) were incubated for the indicated times. Data show the fold change expression of a collection of metabolic genes (RNA-seq). DE upregulated (Log2FC $\geq$ 1) genes are highlighted in green, downregulated genes (Log2FC$\leq$ 1) are highlighted in yellow, and significant FDR values are highlighted in pink. FC (fold change), FDR (false discovery rate), and vs (versus).

| **Genes** | ***Lm* (5 h) vs UT (5 h)** | | ***Lm* (24 h) vs *Lm* (5 h)** | | **IFNγ/LPS (5 h) vs UT (5 h)** | |
| --- | --- | --- | --- | --- | --- | --- |
|  | **Log_2_FC** | **FDR** | **Log_2_FC** | **FDR** | **Log_2_FC** | **FDR** |
| PFKFB3 | 1.24 | 1.19E-14 | 1.80 | 7.67E-07 | 0.58 | 2.18E-02 |
| ACOD1 | 3.11 | 2.49E-38 | 5.91 | 1.50E-71 | 6.28 | 6.80E-134 |
| FASN | 0.86 | 4.85E-07 | -1.93 | 1.12E-10 | -0.88 | 1.96E-05 |
| HADH | -1.65 | 3.41E-34 | -1.95 | 3.22E-15 | -1.71 | 6.20E-13 |
| PC | -1.29 | 2.75E-23 | -2.02 | 5.47E-09 | -1.68 | 8.53E-08 |
| LDHD | -1.52 | 3.09E-16 | -1.04 | 3.76E-04 | -0.64 | 1.65E-02 |
| ME1 | 0.18 | 2.41E-01 | -3.69 | 1.95E-61 | -0.46 | 2.88E-02 |
